# Effect of Sunlight-Induced Isomerisation on the Biotransformation of 4′-Hydroxychalcones by *Yarrowia lipolytica* KCh 71

**DOI:** 10.3390/ijms26189027

**Published:** 2025-09-16

**Authors:** Paweł Chlipała, Tomasz Janeczko, Marcelina Mazur

**Affiliations:** Department of Food Chemistry and Biocatalysis, Wrocław University of Environmental and Life Sciences, Norwida 25, 50-375 Wrocław, Poland

**Keywords:** biotransformation, *Yarrowia lipolytica*, dihydrochalcones, natural products, hydrogenation, chalcone

## Abstract

This study investigates the impact of light exposure on the biotransformation of chalcones in yeast cultures. 4′-Hydroxychalcones, with a hydroxyl group in the A-ring, are characteristic substrates efficiently converted into 4′-hydroxydihydrochalcones—compounds naturally occurring in medicinal plants such as *Glycyrrhiza glabra* (licorice), *Stevia rebaudiana*, and *Angelica keiskei* (ashitaba). These compounds are valued for their bioactivity and are relevant to natural product research. In this research, we present the outcomes of the selective microbial reduction of chalcones to dihydrochalcones using the yeast *Yarrowia lipolytica* KCh 71, cultivated under both light and dark conditions. The aim was to determine whether light exposure affects the efficiency or selectivity of the transformation. Furthermore, the effect of substrate photoisomerisation induced by light was investigated, as the *trans–cis* isomerisation of chalcones may affect their availability and affinity toward enzymatic systems. The resulting metabolites were analysed using chromatographic and spectroscopic methods. No significant differences in transformation efficiency were observed between light and dark conditions. In all tested conditions, the 4′-hydroxydihydrochalcones were obtained with high yield, typically exceeding 90% conversion. Additionally, the selective bioreduction of the α,β-unsaturated bond in selected 4′-hydroxychalcones by the studied yeast culture is an exceptionally efficient process. The primary factor influencing the reaction rate is the structure of the substrate, particularly the number and distribution of methoxyl groups on the B-ring. In addition, we establish biocatalytic access to three target dimethoxy dihydrochalcones—4′-hydroxy-2,4-dimethoxydihydrochalcone (**5a**), 4′-hydroxy-2,5-dimethoxydihydrochalcone (**6a**), and 4′-hydroxy-3,5-dimethoxydihydrochalcone (**7a**)—under mild conditions using *Yarrowia lipolytica* KCh 71. Under preparative-scale conditions (7-day incubation), a minor additional product (≤10%) was detected only for the 4′-hydroxy-2,5-dimethoxydihydrochalcone transformation and identified as 4′,5-dihydroxy-2-methoxydihydrochalcone (**6b**); no such side reaction was observed in short-term experiments.

## 1. Introduction

Chalcones, a class of flavonoid precursors, are known for their distinctive three-carbon α,β-unsaturated carbonyl structure linking two aromatic rings. Dihydrochalcones form a structurally related subclass, resulting from the hydrogenation of the carbon–carbon double bond in the α,β-unsaturated ketone system. Many naturally occurring chalcones and dihydrochalcones exhibit a broad spectrum of valuable biological properties such as antioxidant, anti-inflammatory, anticancer, and antidiabetic activities [1,2,3,4]. The pharmacological relevance of these compounds is reflected in the traditional medicinal use of dragon’s blood (a dark red resin), within which several 4′-hydroxychalcones have recently been identified [5,6].

In addition to their diverse biological activities, chalcones are also known to undergo an interesting photoisomerisation process. As a result, the more stable *trans* isomers typically formed via chemical synthesis are converted to their *cis*-diastereoisomers upon light exposure. In our previous studies, we also observed this process during biotransformation in yeast cultures [7], which prompted interest in its possible influence on the biotransformation rate. Moreover, sunlight may directly impact the biocatalyst, exerting either beneficial or detrimental influence on various metabolic processes.

Some organisms, referred to as photophiles, have the ability to utilise light through the development of radiation-dependent systems, such as photosynthesis, vision, and circadian rhythms, enabling them not only to optimise vital functions, but also to protect themselves from light-induced damage. In contrast, yeasts, including *Saccharomyces cerevisiae*, are classified as skotophiles, which do not use light as a source of energy or environmental information [8]. For such organisms, light functions as a stress factor, triggering the development of defense mechanisms, including the activation of oxidative stress pathways. From an application perspective, the selective bioconversion of chalcones to dihydrochalcones may offer a viable and sustainable alternative to chemical synthesis in the production of nutraceuticals and functional food ingredients [9,10]. Yeast-based biotransformations are particularly attractive due to their mild reaction conditions, high selectivity, and compatibility with food-grade standards [11,12,13]. In the context of chalcone biotransformation in yeast cultures, light exposure can therefore affect both the metabolic activity of microorganisms and the chemical transformation of substrates through the generation of reactive oxygen species and modulation of enzymatic activity. The effect of light on the metabolism of filamentous fungi has been described in the literature. Fungi exhibit diverse responses to light exposure, with significant adjustments occurring in their metabolic pathways either during growth under illumination or following a brief light pulse. Light-induced changes have been observed in the metabolism of carotenoids, carbohydrates, polysaccharides, fatty acids, nucleotides, nucleosides, as well as in the regulation of secondary metabolite production [14]. There are also known cases of light affecting yeast metabolism, described mainly in studies on *Saccharomyces cerevisiae*. Robertson et al. pointed out that visible light alters the metabolic rhythm of yeast by inhibiting respiration [15], whereas Shu et al. reported that light wavelength and intensity significantly affect cell growth and ethanol formation in *S. cerevisiae* [16]. While *Saccharomyces cerevisiae* remains the most commonly utilised yeast in both scientific research and food production, the authorisation of *Yarrowia lipolytica* biomass as a novel food in the EU confirms its safety and highlights its potential for broader application in biocatalytic processes [17,18].

This study aimed to determine whether the biotransformation proceeds similarly for the *cis* and *trans* diastereoisomers of selected 4′-hydroxychalcones and to assess whether light exposure influences both substrate photoisomerisation and the metabolic performance of the biocatalyst—*Yarrowia lipolytica* KCh 71—during selective hydrogenation. A practical objective was to obtain the corresponding 4′-hydroxydihydrochalcones with high yield and selectivity, including access to 4′-hydroxy-2,4-dimethoxydihydrochalcone (**5a**), 4′-hydroxy-2,5-dimethoxydihydrochalcone (**6a**), and 4′-hydroxy-3,5-dimethoxydihydrochalcone (**7a**), given their biological relevance and occurrence in medicinal plants. Preparative-scale experiments with prolonged incubation were conducted to verify the stability of the hydrogenation products.

## 2. Results and Discussion

The biotransformation of 4′-hydroxychalcones **1**–**7** by *Yarrowia lipolytica* KCh 71 was investigated under different light conditions. The chemical structures of the substrates and the corresponding hydrogenated products are presented in Table 1. In all cases, the yeast selectively reduced the α,β-unsaturated double bond, affording the respective dihydrochalcones. Significant product formation was detected after 30 min, and in most cases ≥90% conversion was reached within 3 h. Preparative-scale experiments were intentionally extended to seven days to secure sufficient quantities for NMR analysis, assess product stability, and reveal potential minor secondary transformations.

In addition, the research aimed to evaluate the influence of light both on the course of the biotransformation itself and on the isomerisation of the substrate, in connection with the overall biocatalytic process. Understanding these effects could provide valuable insights for optimising reaction conditions and improving overall process efficiency.

**Figure 1:**
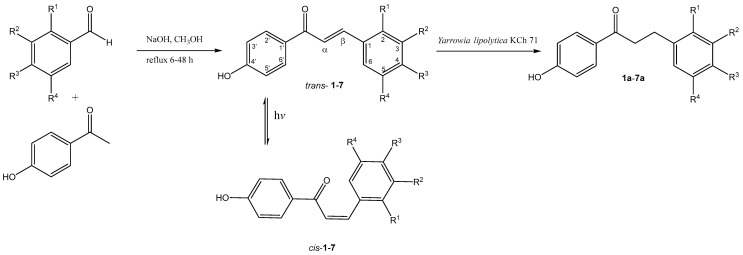
Synthesis of 4′-hydroxychalcones, light-induced isomerisation of chalcones solutions, and their subsequent transformations to 4′-hydroxydihydrochalcones using *Yarrowia lipolytica* KCh 71.

The substrates of the biotransformation were seven 4′-hydroxychalcones, one without additional methoxyl groups, three with one methoxyl group located in the *ortho*, *meta*, or *para* position of the B-ring of the chalcone, and three containing two methoxyl groups. **Table 1** and **Figure 1** show the exact position of the substituents in the B-rings of the biotransformation substrates.

All 4′-hydroxychalcones were obtained via Claisen–Schmidt condensation of 4-hydroxyacetophenone with benzaldehyde, methoxybenzaldehyde, or dimethoxybenzaldehyde featuring different substitution patterns (**Figure 1**). The structures of the resulting compounds were determined by in-depth NMR analysis including COSY, HSQC, and HMBC correlation spectra. For all synthesised chalcone derivatives, a notable downfield shift of the olefinic protons H-α and H-β was observed. These signals are present as two distinct doublets, characterised by a coupling constant of approximately 15.7 Hz, as seen in the ^1^H NMR spectra. This high coupling constant is indicative of a *trans* configuration of the double bond in the products structures. Furthermore, the presence of a C=C double bond in the product molecule corroborates the chemical shift of carbon atoms C-α and C-β towards the low field (δ above 119 ppm). The obtained structures were consistent with existing literature data. According to our literature search, dihydrochalcones **6a**, **6b**, and **7a** have not been previously reported.

Chalcones can undergo a wide array of microbial biotransformations, enhancing their structural diversity and potential bioactivity. Those transformations can include hydroxylation and demethylation, mediated by cytochrome P450 enzymes in fungal cultures (*Aspergillus alliaceus*), leading to multiple flavonoid derivatives, including flavanones [19]. Glycosylation and O-methylglycosylation are also common, often by filamentous fungi like *Beauveria*, *Isaria*, *Penicillium*, *Absidia*, or *Rhizopus*, producing new glycosides of chalcones or dihydrochalcones [20]. Various types of reduction processes have also been reported in the scientific literature. The formation of dihydrochalcones is most commonly associated with the selective reduction of the α,β-unsaturated double bond (C=C) in the chalcone scaffold. However, reductions targeting the carbonyl group (C=O) of chalcones have also been described, leading to the formation of corresponding alcohol derivatives. These reduction reactions can be catalysed by microbial strains or isolated enzymes and are often highly regio- and stereoselective, offering efficient and environmentally friendly routes to structurally diverse chalcone analogues.

**Figure 2:**
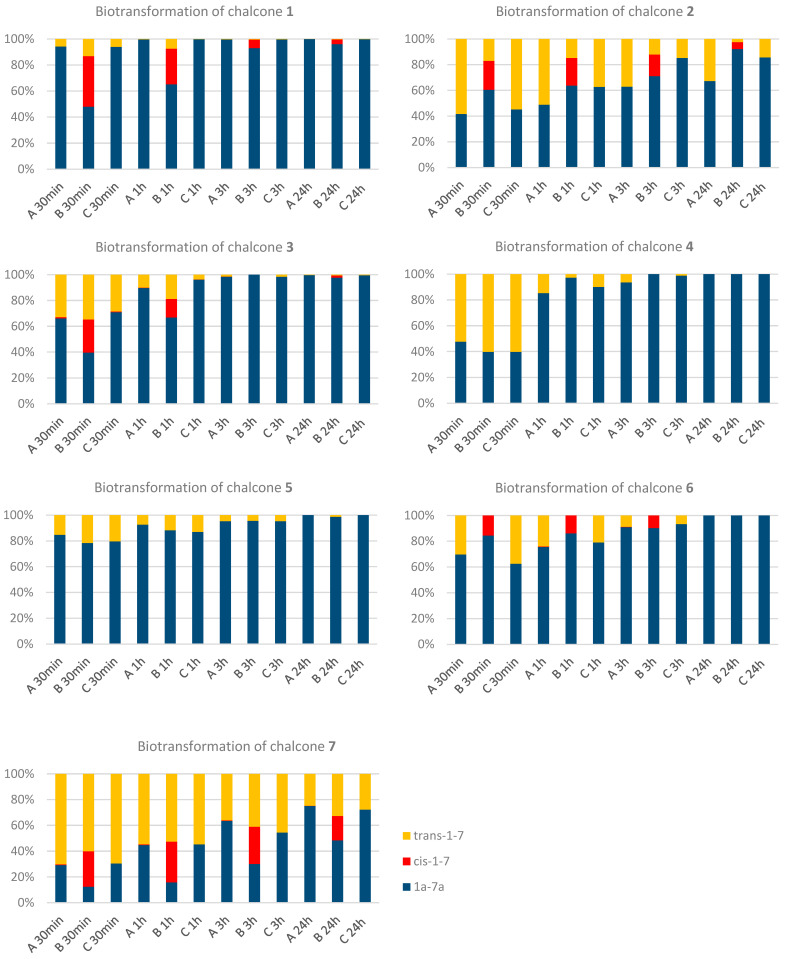
Percentage composition of the mixture during the biotransformation of 4′-hydroxychalcone **1**–**7** in different conditions: Method A—standard biotransformation, Method B—isomerisation of the substrate, Method C—biotransformation without access to light.

Biotransformations were performed under three distinct experimental conditions to evaluate the influence of light exposure on the process (**Figure 2**). In Method A, freshly prepared solutions of *trans*-4′-hydroxychalcones were aseptically added to the *Yarrowia lipolytica* KCh 71 culture. The biotransformation was conducted in a well-illuminated laboratory environment where cultures were exposed to natural sunlight following the day–night cycle, receiving daylight during daytime hours and remaining without artificial illumination at night. (Method A). In Method B, *trans*-chalcones were initially dissolved in DMSO and subjected to photochemical isomerisation under direct sunlight. The resulting mixture of *trans*- and *cis*-chalcones (**Table 2**) was then introduced into the *Yarrowia lipolytica* KCh 71 culture and the biotransformation was carried out in the same brightly lit conditions with natural sunlight exposure. In Method C, to assess the effects of light exclusion, freshly dissolved in DMSO *trans*-chalcones were added to the yeast culture and the entire biotransformation process was performed in complete darkness, with culture flasks additionally wrapped in aluminium foil to prevent any light penetration.

**Figure 3:**
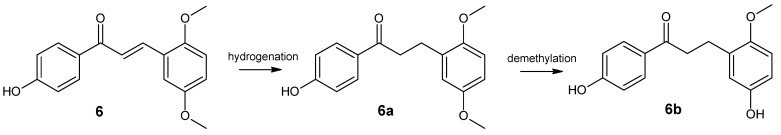
The minor product observed for the 2,5-dimethoxy substrate under preparative conditions was detected only after 7 days and is therefore not reflected in the 24 h datasets shown.

The results of the experiments are shown as **Figure 2** and confirm previous findings that the yeast strain used is an effective biocatalyst in the selective hydrogenation of carbon–carbon double bonds in chalcone structures [7,21,22]. Although significant amounts of products were already observed after 30 min, and in most cases conversions ≥90% were reached within 3 h, preparative runs were intentionally extended to seven days to secure NMR quantities, assess product stability, and reveal minor secondary transformations (**6b**). The primary distinction observed in proton magnetic resonance spectra is the absence of olefin protons. Instead of the characteristic doublets, there are multiplets from methylene protons CH_2_-α, and CH_2_-β significantly shifted towards the higher field (around 3 ppm). A similar change is observed in the ^13^C NMR spectra of biotransformation products. The carbon atoms of the methylene groups appear as signals at a notably higher field, approximately 40 ppm for C-α and 30 ppm for C-β, whereas the presence of a carbonyl carbon atom is confirmed by signals above 197 ppm. Consistent with our previous work [7,9,22,23] and with the short-term experiments herein, no additional products were detected for the majority of substrates. An exception was observed only under preparative conditions for the 4′-hydroxy-2,5-dimethoxydihydrochalcone: when the reaction was extended to seven days, a minor component (≤10% of the mixture) was isolated alongside **6a** and identified as 4′,5-dihydroxy-2-methoxydihydrochalcone (**6b**) (**Figure 3**). The assignment follows directly from the NMR data: in ^1^H NMR, two phenolic OH singlets are present (δ_H_ = 9.16 and 7.77) and only one methoxy singlet is observed (δ_H_ = 3.76); HSQC shows the expected correlation of this methoxy signal with the methoxy carbon at δ_C_ = 56.12. The ^13^C NMR spectrum displays oxygenated aromatic carbons at δ_C_ ≈ 151.8 and 151.9 (assigned to C-5 and C-2), along with a carbonyl at δ_C_ ≈ 198.0 and aliphatic side-chain carbons at δ_C_ ≈ 38.9 (C-α) and 26.3 (C-β), all consistent with a dihydrochalcone framework bearing a single methoxy group. By contrast, **6a** shows two methoxy singlets in ^1^H (δ_H_ = 3.79 and 3.71) and two methoxy carbons in ^13^C NMR (δ_C_ = 56.09 and 55.68). Taken together, these features substantiate selective demethylation under prolonged incubation and support the structure of **6b**.

The biotransformation rate of individual substrates was primarily influenced by the position and number of methoxy substituents. The most rapid transformation was observed for 4′-hydroxychalcone **1**, which lacks additional methoxy groups on ring B. Most of the processes were extremely fast, with over 90% conversion of substrates to dihydrochalcones observed after only 3 h, regardless of the process conditions used. Compared to similar biotransformations described in the literature, the conversion rates observed with the *Yarrowia lipolytica* KCh 71 strain are remarkably high. Previous studies using other biocatalysts often report yields between 60–80% under comparable conditions, while *Y. lipolytica* KCh 71 has demonstrated conversion rates exceeding 98–99% within just a few hours for various chalcone derivatives [23,24,25,26,27]. This high efficiency highlights the superior enzymatic activity and potential of this strain for selective biotransformations, making it a promising biocatalyst for sustainable and industrial-scale production of dihydrochalcones [7,9,28]. Notably, only 4′-hydroxychalcone **2** and **7** showed significantly lower conversions, which is particularly evident for 4′-hydroxychalcone **7**. Across the dimethoxychalcones, the corresponding targets were obtained in each case: 4′-hydroxy-2,4-dimethoxychalcone → 4′-hydroxy-2,4-dimethoxydihydrochalcone (**5a**), 4′-hydroxy-2,5-dimethoxychalcone → 4′-hydroxy-2,5-dimethoxydihydrochalcone (**6a**), and 4′-hydroxy-3,5-dimethoxychalcone → 4′-hydroxy-3,5-dimethoxydihydrochalcone (**7a**). Although the 4′-hydroxy-3,5-dimethoxychalcone (**7**) displayed the slowest conversion within 24 h, the desired product **7a** was nevertheless isolated and fully characterised. Complete NMR datasets for **5a**, **6a**, **6b**, and **7a** are provided in the Supplementary Materials (Figures S1–S97).

During the biotransformation of *trans*-4′-hydroxychalcones, despite the experiments being conducted in a well-lit environment with exposure to sunlight (Method A), no significant isomerisation of the substrates to *cis*-diastereoisomers was observed. This lack of isomerisation may be attributed to the rapid conversion of the substrate to dihydrochalcone and the substantial growth of biomass, which could have constrained the isomerisation process.

Notably, no significant differences were observed in substrate conversion between sunlight-exposed conditions (Method A) and those conducted in the absence of light (Method C). This finding suggests that light does not influence the enzymatic hydrogenation reaction in the yeast culture under investigation, nor does it affect the metabolic pathways that govern the transformation of the introduced xenobiotics.

Building upon our previous findings that *trans*-4′-hydroxychalcones exhibit isomerisation when exposed to light, we aimed to examine the impact of this phenomenon on the biotransformation process. *Trans*-*cis* photoisomerisation is a photochemical process in which a molecule containing a double bond—such as a C=C or N=N bond—undergoes a geometric transformation from the *trans* to the *cis* isomer upon absorption of light. This transformation involves the disruption of the planarity around the double bond, allowing rotation and rearrangement of the substituents into the *cis* configuration [29]. The scientific literature provides substantial evidence of the photochemical behaviour of chalcones. The dynamics of chalcone isomerisation under varying lighting conditions include significant changes in the ratio of isomers and, for appropriately structured substrates, the potential formation of cyclisation products at different wavelengths, have been documented [30,31,32]. Researchers have observed that exposure to sunlight results in a notable increase in the proportion of *cis* isomers, underscoring the role of light in modulating chalcone isomerisation. An interesting aspect associated with this phenomenon is the investigation of light-induced chalcone isomerisation within the framework of photodynamic therapy. The researchers demonstrated that the capacity to regulate the *trans*-to-*cis*-isomer ratio through light exposure could enhance the therapeutic efficacy of chalcone-based photosensitisers [33,34]. This underscores the potential of light modulation in optimising the biological applications of chalcones.

Given the different biological activity of diastereoisomers, we also aimed to determine whether this affects yeast as a biocatalyst and whether a change in the geometry of the isomer will affect the biotransformation rate. As a result, the substrates underwent isomerisation under light conditions in DMSO, leading to the formation of mixtures of diastereoisomers. The isomerisation of substrates was carried out until the equilibrium state specific to a given compound was reached. The composition of the diastereoisomers obtained through this method is presented in Table 2. The presence of *cis*-chalcones was corroborated through the analysis of spectroscopic data. The primary distinction observed in the ^1^H NMR of *cis*-analogues, as opposed to *trans*-chalcones, is the upfield shift of signals from olefin protons and a notable reduction in the coupling constant to approximately 13 Hz. Because the photoisomerisation did not reach 100% conversion, the NMR spectra of samples annotated as *cis*-chalcones also show residual signals of the *trans*-isomers.

The efficacy of the chalcone isomerisation process varies according to the number and position of methoxy groups on the B-ring. The process was most effective in the case of unsubstituted 4′-hydroxychalcone (**1**) and 4′-hydroxy-3-methoxychalcone (**3**). The lowest isomerisation efficiency was observed for chalcones with an additional methoxy group at carbon atom C-4 in ring B (chalcone 4 and 5).

The substrate mixtures prepared in this way were then subjected to biotransformation under light conditions (Method B). For 4′-hydroxychalcones 4 and 5, where no significant isomerisation of the substrates was observed, the biotransformation process proceeds at a rate comparable to that observed for method A and C. Significant variations in conversion rates were observed for chalcone lacking substitution in ring B (1), as well as for chalcones with 3-methoxy (3) and 3,5-dimethoxy substituents (7). In the majority of the tested mixtures, these differences became less pronounced after 24 h of the process when compared to the standard biotransformation (Method A). Notably, only the isomerised 3,5-dimethoxychalcones (3) exhibited a conversion rate that did not exceed 50% after 24 h.

In general, 4′-hydroxydihydrochalcones obtained in this study are structurally similar to natural compounds with well-documented biological activities, including antioxidant, antidiabetic, and anti-inflammatory effects. Their presence in medicinal plants such as *Stevia rebaudiana* and *Glycyrrhiza glabra* further emphasises the potential practical relevance of the described biotransformation pathway [29,30,31,32].

## 3. Materials and Methods

### 3.1. Chemicals and Analysis

All reagents, including chemicals (benzaldehyde, 2-methoxybenzaldehyde, 3-methoxybenzaldehyde, 4-methoxybenzaldehyde, 2,4-dimethoxybenzaldehyde, 2,5-dimethoxybenzaldehyde, and 3,5-dimethoxybenzaldehyde, 4-hydroxyacetophenone) were purchased from Sigma-Aldrich (St. Louis, MO, USA) and Carbosynth (Compton, Berkshire, UK).

The progress of the reactions was monitored by gas chromatography (GC) using an Agilent Technologies 6890N instrument (Agilent Technologies, Warsaw, Poland) equipped with a DB-5MS capillary column (non-polar phenyl arylene polymer, 30 m × 0.32 mm × 0.25 µm). Hydrogen was used as the carrier gas. The GC conditions were as follows: injector temperature at 250 °C, flame ionisation detector (FID) at 250 °C, and a temperature program starting at 80 °C, increasing to 200 °C at a rate of 25 °C/min, then to 300 °C at 30 °C/min, with a final hold at 300 °C for 3 min.

Product purification was performed using a PuriFlash XS520Plus system with a 30 µm silica gel column (Interchim, Montluçon, France).

NMR spectra were recorded using a JEOL 400 MHz Year Hold Magnet spectrometer (JEOL, Freising, Germany) and a Bruker Avance II 600 MHz spectrometer (Bruker, Rheinstetten, Germany). Measurements were carried out in DMSO-*d*_6_, CDCl_3_, or acetone-*d*_6_. Residual solvent signals were used as internal references.

### 3.2. Chemical Synthesis

The chalcone derivatives were synthesised via Claisen–Schmidt condensation between benzaldehyde or its methoxy-substituted analogues (2-methoxybenzaldehyde, 3-methoxybenzaldehyde, 4-methoxybenzaldehyde, 2,4-dimethoxybenzaldehyde, 2,5-dimethoxybenzaldehyde, and 3,5-dimethoxybenzaldehyde) with 4-hydroxyacetophenone, following the procedure described earlier [7,9,33]. The structures of the compounds obtained by chemical synthesis and biotransformation are shown in **Figure 1**. The UV spectrum and NMR analyses are presented in the Supplementary Materials Section (Figures S1–S97). The spectral data for these compounds are consistent with the literature [9,33,34,35]:

*trans*-4′-hydroxychalcone (*trans*-**1**)

^1^H NMR (600 MHz; DMSO-*d*_6_) δ (ppm): 10.44 (s, 1H, C-4′-OH), 8.05–8.10 (m, 2H, H-2′, and H-6′), 7.92 (d, 1H, *J* = 15.6 Hz, H-α), 7.85–7.89 (m, 2H, H-2, and H-6), 7.68 (d, 1H, *J* = 15.6 Hz, H-β), 7.42–7.47 (m, 3H, H-3, H-4, and H-5), 6.88–6.92 (m, 2H, H-3′, and H-5′). ^13^C NMR (151 MHz, DMSO-*d*_6_) δ (ppm): 187.14 (C=O), 162.24 (C-4′), 142.76 (C-β), 134.91 (C-1), 131.24 (C-2′ and C-6′), 130.35 (C-4), 129.12 (C-1′), 128.92 (C-2 and C-6), 128.74 (C-3 and C-5), 122.11 (C-α), 115.41 (C-3′ and C-5′).

*cis*-4′-hydroxychalcone (*cis*-**1**)

^1^H NMR (600 MHz; DMSO-*d*_6_) δ (ppm): 10.48 (s, 1H, C-4′-OH), 7.81–7.83 (m, 2H, H-2′, and H-6′), 7.36–7.32 (m, 2H, H-2, and H-6), 7.23–7.28 (m, 3H, H-3, H-4, and H-5), 6.93 (d, 1H, *J* = 13.0 Hz, H-β), 6.80–6.84 (m, 2H, H-3′, and H-5′), 6.71 (d, 1H, *J* = 13.0 Hz, H-α). ^13^C NMR (151 MHz, DMSO-*d*_6_) δ (ppm):192.98 (C=O), 162.55 (C-4′), 136.65 (C-β), 135.44 (C-1), 131.43 (C-2′ and C-6′), 129.05 (C-2 and C-6), 128.57 (C-4), 128.36 (C-3 and C-5), 128.28 (C-1′), 128.11 (C-α), 115,56 (C-3′ and C-5′).

4′-hydroxydihydrochalcone (**1a**)

^1^H NMR (400 MHz; DMSO-*d*_6_) δ (ppm): 10.37 (s, 1H, C-4′-OH), 7.84–7.87 (m, 2H, H-2′, and H-6′), 7.24–7.28 (m, 4H, H-2, H-3, H-5, and H-6), 7.14–7.19 (m, 1H, H-4), 6.81–6.86 (m, 2H, H-3′, and H-5′), 3.23 (t, 2H, *J* = 7.5 Hz, CH_2_-α), 2.90 (t, 2H, *J* = 7.5 Hz, CH_2_-β). ^13^C NMR (100 MHz, DMSO-*d*_6_) δ (ppm): 197.41 (C=O), 162.07 (C-4′), 141.53 (C-1), 130.59 (C-2′ and C-6′), 128.48 and 128.36 (C-1′, C-2, C-3, C-5, and C-6), 125.92 (C-4), 115.31 (C-3′ and C-5′), 39.04 (C-α), 29.48 (C-β).

*trans*-4′-hydroxy-2-methoxychalcone (*trans*-**2**)

^1^H NMR (600 MHz; DMSO-*d*_6_) δ (ppm): 10.42 (s, 1H, C-4′-OH), 8.02–8.06 (m, 2H, H-2′, and H-6′), 7.99 (d, 1H, *J* = 15.8 Hz, H-β), 7.94 (dd, 1H, *J* = 7.7 and 1.6 Hz, H-6), 7.85 (d, 1H, *J* = 15.8 Hz, H-α), 7.43 (ddd, 1H, *J* = 8.2, 7.5 and 1.6 Hz, H-4), 7.10 (d, 1H, *J* = 8.2 Hz, H-3), 7.02 (t, 1H, *J* = 7.5 Hz, H-5), 6.87–6.92 (m, 2H, H-3′, and H-5′), 3.89 (s, 3H, C-2-OC*H*_3_),. ^13^C NMR (151 MHz, DMSO-*d*_6_) δ (ppm): 187.31 (C=O), 162.14 (C-4′), 158.14 (C-2), 137.29 (C-β), 132.01 (C-4), 131.01 (C-2′ and C-6′), 129.26 (C-6), 128.37 (C-1′), 123.16 (C-1), 121.91 (C-α), 120.71 (C-5), 115.42 (C-3′ and C-5′), 111.78 (C-3), 55.72 (C-2-O*C*H_3_).

*cis*-4′-hydroxy-2-methoxychalcone (*cis*-**2**)

^1^H NMR (600 MHz; DMSO-*d*_6_) δ (ppm): 10.41 (s, 1H, C-4′-O*H*), 7.75–7.78 (m, 2H, H-2′, and H-6′), 7.22 (ddd, 1H, *J* = 7.8, 7.5, 1.6 Hz, H-4), 7.15 (dd, 1H, *J* = 7.5, 1.6 Hz, H-6), 7.04 (d, 1H, *J* = 12.8 Hz, H-β), 6.94 (d, 1H, *J* = 7.8 Hz, H-3), 6.77–6.80 (m, 2H, H-3′, and H-5′), 6.76 (t, 1H, *J* = 7.5, 0.6 Hz, H-5), 6.70 (d, 1H, *J* = 12.8 Hz, H-α), 3.71 (s, 3H, C-2-OC*H*_3_). ^13^C NMR (151 MHz, DMSO-*d*_6_) δ (ppm): 192.32 (C=O), 162.20 (C-4′), 132.89 (C-β), 131.18 (C-2′ and C-6′), 130.09 (C-4), 129.81 (C-6), 128.57 (C-1′), 127.74 (C-α), 124.38 (C-1),119.99 (C-5), 115.36 (C-3′ and C-5′), 111.05 (C-3),156.72 (C-2), 55.32 (C-2-O*C*H_3_).

4′-hydroxy-2-methoxydihydrochalcone (**2a**)

^1^H NMR (600 MHz; DMSO-*d*_6_) δ (ppm): 10.33 (s, 1H, C-4′-O*H*), 7.83–7.86 (m, 2H, H-2′, and H-6′), 7.18 (td, 1H, *J* = 7.4, 1.7 Hz, H-4), 7.17 (dd, 1H, *J* = 7.4, 0.7 Hz, H-6), 6.94 (dd, 1H, *J* = 8.6, 0.8 Hz, H-3), 6.85 (td, 1H, *J* = 7.4, 1.0 Hz, H-5), 6.81–6.84 (m, 2H, H-3′, and H-5′), 3.78 (s, 3H, C-2-OC*H*_3_), 3.12–3.16 (m, 2H, CH_2_-α), 2.83–2.87 (m, 2H, CH_2_-β). ^13^C NMR (151 MHz, DMSO-*d*_6_) δ (ppm): 197.56 (C=O), 161.95 (C-4′), 157.11 (C-2), 130.45 (C-2′ and C-6′), 129.61 (C-4), 129.01 (C-1′), 128.21 (C-1), 127.36 (C-6), 120.24 (C-5), 115.22 (C-3′ and C-5′), 110.55 (C-3), 55.25 (C-2-O*C*H_3_), 37.59 (C-α), 24.94 (C-β).

*trans*-4′-hydroxy-3-methoxychalkone (*trans*-**3**)

^1^H NMR (600 MHz; DMSO-*d*_6_) δ (ppm): 10.43 (s, 1H, C-4′-O*H*), 8.04–8.12 (m, 2H, H-2′, and H-6′), 7.92 (d, 1H, *J* = 15.6 Hz, H-α), 7.65 (d, 1H, *J* = 15.6 Hz, H-β), 7.46 (t, 1H, *J* = 1.9 Hz, H-2), 7.41 (d, 1H, *J* = 7.7 Hz, H-6), 7.36 (t, 1H, *J* = 7.8 Hz, H-5), 7.01 (ddd, 1H, *J* = 8.0, 2.4, 0.8 Hz, H-4), 6.88–6.92 (m, 2H, H-3′, and H-5′), 3.83 (s, 3H, C-3-OC*H*_3_). ^13^C NMR (151 MHz, DMSO-*d*_6_) δ (ppm): 187.15 (C=O), 162.58 (C-4′), 159.00 (C-3), 136.74 (C-1), 136.05 (C-β), 131.42 (C-2′ and C-6′), 129.45 (C-5), 129.10 (C-1′), 128.25 (C-α), 121.55 (C-6), 116.45 (C-4), 115.40 (C-3′ and C-5′), 113.20 (C-2), 55.31 (C-3-O*C*H_3_).

*cis*-4′-hydroxy-3-methoxychalcone (*cis*-**3**)

^1^H NMR (600 MHz; DMSO-*d*_6_) δ (ppm): 10.49 (s. 1H, C-4′-OH), 7.80–7.83 (m, 2H, H-2′, and H-6′), 7.17 (t, 1H, *J* = 7.9 Hz, H-5), 7.46 (dd, 1H, *J* = 2.2, 1.8 Hz, H-2), 6.89–6.91 (m, 1H, H-6). 6.89 (d, 1H, *J* = 13.0 Hz, H-β), 6.81–6.84 (m, 2H, H-3′, and H-5′), 6.81 (ddd, 1H, *J* = 8.1, 2.6, 0.7 Hz, H-4). 6.68 (d, 1H, *J* = 13.0 Hz, H-α). 3.63 (s, 3H, C-3-OC*H*_3_). ^13^C NMR (151 MHz, DMSO-*d*_6_) δ (ppm): 193.28 (C=O), 162.58 (C-4′), 159.00 (C-3), 136.74 (C-1), 136.05 (C-β), 131.42 (C-2′ and C-6′), 129.45 (C-5), 128.52 (C-α), 128.24 (C-1′), 121.55 (C-6), 115.58 (C-3′ and C-5′), 114.29 (C-2), 114.20 (C-4), 54.98 (C-3-O*C*H_3_).

4′-hydroxy-3-methoxydihydrochalcone (**3a**)

^1^H NMR (600 MHz; CDCl_3_) δ (ppm): 7.87–7.91 (m, 2H, H-2′, and H-6′). 7.21 (t, 1H, *J* = 7.9 Hz, H-5). 7.04 (s, 1H, C-4′-OH). 6.87–6.90 (m, 2H, H-3′, and H-5′). 6.83 (t, 1H, *J* = 7.6 Hz, H-6). 7.46 (dd, 1H, *J* = 2.1, 1.6 Hz, H-2). 6.75 (dd, 1H, *J* = 8.2, 2.5 Hz, H-4), 3.79 (s, 3H, C-3-OC*H*_3_). 3.22–3.28 (m, 2H, CH_2_-α), 3.00–3.06 (m, 2H, CH_2_-β). ^13^C NMR (151 MHz, CDCl_3_) δ: 199.20 (C=O), 161.04 (C-4′), 159.77 (C-3), 142.98 (C-1), 130.95 (C-2′ and C-6′), 129.67 (C-5), 129.58 (C-1′), 120.94 (C-6), 115.63 (C-3′ and C-5′), 114.35 (C-2), 111.58 (C-4), 55.33 (C-3-O*C*H_3_), 40.14 (C-α), 30.62 (C-β).

*trans*-4′-hydroxy-4-methoxychalcone (*trans*-**4**)

1H NMR (600 MHz; DMSO-*d*_6_) δ (ppm): 10.41 (s, 1H, C-4′-OH), 8.03–8.06 (m, 2H, H-2′, and H-6′), 7.79–7.83 (m, 2H, H-2, and H-6), 7.76 (d, 1H, *J* = 15.5 Hz, H-α), 7.65 (d, 1H. *J* = 15.5 Hz, H-β), 6.98–7.02 (m, 2H, H-3, and H-5), 6.87–6.91 (m, 2H, H-3′, and H-5′), 3.81 (s, 3H, C-4-OC*H*_3_). ^13^C NMR (151 MHz, DMSO-*d*_6_) δ (ppm): 187.18 (C=O), 162.07 (C-4′), 161.20 (C-4), 142.80 (C-β), 131.12 (C-2′ and C-6′), 130.64 (C-2 and C-6), 129.41 (C-1′), 127.58 (C-1), 119.64 (C-α), 114.46 (C-3′ and C-5′), 115.41 (C-5 and C-3), 55.43 (C-4-O*C*H_3_).

*cis*-4′-hydroxy-4-methoxychalcone (*cis*-**4**)

1H NMR (600 MHz; DMSO-*d*_6_) δ (ppm): 10.42 (s, 1H, C-4′-OH), 7.82–7.85 (m, 2H, H-2′, and H-6′), 7.42–7.45 (m, 2H, H-2, and H-6), 6.86 (d, 1H, *J* = 12.8 Hz. H-β), 6.83–6.86 (m, 2H, H-3, and H-5), 6.81–6.84 (m, 2H, H-3′, and H-5′), 6.61 (d, 1H, *J* = 12.8 Hz, H-α). 3.72 (s, 3H, C-4-OC*H*_3_). ^13^C NMR (151 MHz, DMSO-*d*_6_) δ (ppm): 192.39 (C=O), 162.38 (C-4′), 159.72 (C-4), 137.42 (C-β), 131.30 (C-2′ and C-6′), 131.24 (C-2 and C-6), 128.72 (C-1′), 127.91 (C-1), 124.93 (C-α), 115.50 (C-3′ and C-5′), 113.72 (C-5 and C-3), 55.22 (C-4-O*C*H_3_).

4′-hydroxy-4-methoxydihydrochalcone (**4a**)

^1^H NMR (600 MHz; CDCl_3_) δ (ppm): 7.87–7.91 (m, 2H, H-2′, and H-6′), 7.13–7.17 (m, 2H, H-2, and H-6), 7.00 (s, 1H, C-4′-OH), 6.87–6.91 (m, 2H, H-3′, and H-5′), 6.82–6.85 (m, 2H, H-3, and H-5), 3.78 (s, 3H, C-4-OC*H*_3_), 3.20–3.24 (m, 2H, CH_2_-α), 2.92–3.02 (m, 2H, CH_2_-β). ^13^C NMR (151 MHz, CDCl_3_) δ (ppm): 199.46 (C=O), 161.01 (C-4′), 158.03 (C-4), 133.40 (C-1), 130.97 (C-1′), 130.96 (C-2′ and C-6′), 129.47 (C-2 and C-6),115.63 (C-3′ and C-5′), 114.10 (C-5 and C-3), 55.45 (C-4-O*C*H_3_), 40.50 (C-α), 29.78 (C-β).

*trans*-4′-hydroxy-2,4-dimethoxychalcone (*trans*-**5**)

^1^H NMR (600 MHz; DMSO-*d*_6_) δ (ppm): 10.36 (s, 1H, C-4′ -OH), 7.98–8.04 (m, 2H, H-2′, and H-6′), 7.93 (d, 1H, *J* = 15.7 Hz, H-β), 7.89 (d, 1H, *J* = 8.5 Hz, H-6), 7.73 (d, 1H, *J* = 15.7 Hz, H-α), 6.86–6.91 (m, 2H, H-3′, and H-5′), 6.63 (d, 1H, *J* = 2.3 Hz, H-3), 6.61 (dd, 1H, *J* = 8.6, 2.3 Hz, H-5), 3.89 (s, 3H, C-2-OC*H*_3_), 3.83 (s, 3H, C-4-OC*H*_3_). ^13^C NMR (151 MHz, DMSO-*d*_6_) δ (ppm): 187.25 (C=O), 162.85 (C-4), 161.90 (C-4′), 159.80 (C-2), 137.52 (C-β), 130.93 (C-2′ and C-6′), 129.93 (C-6), 129.54 (C-1′), 119.14 (C-α), 116.13 (C-1), 115.34 (C-3′ and C-5′), 106.28 (C-5), 98.32 (C-3), 55.83 (C-2-O*C*H_3_), 55.54 (C-4-O*C*H_3_).

*cis*-4′-hydroxy-2,4-dimethoxychalcone (*cis*-**5**)

^1^H NMR (600 MHz; DMSO-*d*_6_) δ (ppm): 10.35 (s, 1H, C-4′-O*H*), 7.77–7.80 (m, 2H, H-2′, and H-6′), 7.28 (d, 1H, *J* = 8.6 Hz, H-6), 7.00 (d, 1H, *J* = 12.8 Hz, H-β), 6.78–6.82 (m, 2H, H-3′, and H-5′), 6.59 (d, 1H, *J* = 12.8 Hz, H-α), 6.50 (d, 1H, *J* = 2.4 Hz, H-3), 6.38 (dd, 1H, *J* = 8.6, 2.4 Hz, H-5), 3.74 (s, 3H, C-2-OC*H*_3_), 3.73 (s, 3H, C-4-OC*H*_3_). ^13^C NMR (151 MHz, DMSO-*d*_6_) δ (ppm): 192.10 (C=O), 162.07 (C-4′), 161.33 (C-4), 158.27 (C-2), 132.86 (C-β), 131.11 (C-2′ and C-6′), 130.99 (C-6), 128.88 (C-1′), 124.99 (C-α), 116.96 (C-1), 115.34 (C-3′ and C-5′), 104.89 (C-5), 98.00 (C-3), 55.34 (C-2-O*C*H_3_), 55.47 (C-5-O*C*H_3_).

4′-hydroxy-2,4-dimethoxydihydrochalcone (**5a**)

^1^H NMR (600 MHz; Acetone-*d*_6_) δ (ppm): 9.15 (s, 1H, -OH) 7.89–7.93 (m, 2H, H-2′ and H-6′), 7.09 (d, 1H, *J* = 8.2 Hz, H-6), 6.90–6.93 (m, 2H, H-3′, and H-5′), 6.52 (d, 1H, *J* = 2.4 Hz, H-3), 6.43 (dd, 1H, *J* = 8.2 and 2.4 Hz, H-5), 3.82 (s, 3H, C-2-OC*H*_3_), 3.76 (s, 3H, C-4-OC*H*_3_), 3.11–3.16 (m, 2H, CH_2_-α), 2.86–2.91 (m, 2H, CH_2_-β). ^13^C NMR (151 MHz, Acetone-*d*_6_) δ (ppm): 198.26 (C=O), 162.54 (C-4′), 160.57 (C-4), 159.31(C-2), 131.30 (C-2′ and C-6′), 130.99 (C-6), 130.29 (C-1′), 122.57 (C-1), 116.00 (C-3′ and C-5′), 105.07 (C-5), 99.11 (C-3), 55.66 (C-2-OC*H*_3_), 55.53 (C-4-OC*H*_3_), 39.20 (C-α) 25.80 (C-β).

*trans*-4′-hydroxy-2,5-dimethoxychalcone (*trans*-**6**)

^1^H NMR (600 MHz; DMSO-*d*_6_) δ (ppm): 10.42 (s, 1H, C-4′ -OH), 8.04–8.09 (m, 2H, H-2′, and H-6′), 7.98 (d, 1H, *J* = 15.7 Hz, H-β), 7.87 (d, 1H, *J* = 15.7 Hz, H-α), 7.53 (d, 1H, *J* = 2.5 Hz, H-6), 7.04 (d, 1H, *J* = 9.0 Hz, H-3), 7.04 (dd, 1H, *J* = 9.0, 2.5 Hz, H-4), 6.87–6.92 (m, 2H, H-3′, and H-5′), 3.83 (s, 3H, C-2-OC*H*_3_), 3.79 (s, 3H, C-5-OC*H*_3_). ^13^C NMR (151 MHz, DMSO-*d*_6_) δ (ppm): 187.26 (C=O), 162.16 (C-4′), 153.28 (C-5), 152.59 (C-2), 137.03 (C-β), 131.20 (C-2′ and C-6′), 129.23 (C-1′), 123.72 (C-1), 122.09 (C-α), 117.82 (C-4), 115.38 (C-3′ and C-5′), 113.01 (C-3), 112.47 (C-6), 56.15 (C-2-O*C*H_3_), 55.70 (C-5-O*C*H_3_).

*cis*-4′-hydroxy-2,5-dimethoxychalcone (*cis*-**6**)

^1^H NMR (600 MHz; DMSO-*d*_6_) δ (ppm): 10.44 (s, 1H, C-4′-O*H*), 7.75–7.79 (m, 2H, H-2′, and H-6′), 7.12 (d, 1H, *J* = 12.8 Hz, H-β), 6.89 (d, 1H, *J* = 8.9 Hz, H-3), 6.78–6.82 (m, 3H, H-4, H-3′, and H-5′), 6.77 (d, 1H, *J* = 3.0 Hz, H-6), 6.69 (d, 1H, *J* = 12.9 Hz, H-α), 3.66 (s, 3H, C-2-*O*C*H*_3_), 3.53 (s, 3H, C-5-*O*C*H*_3_). ^13^C NMR (151 MHz, DMSO-*d*_6_) δ (ppm): 192.56 (C=O), 162.26 (C-4′), 152.39 (C-5), 151.32 (C-2), 132.15 (C-β), 131.24 (C-2′ and C-6′), 131.19 (C-3′ and C-5′), 128.52 (C-1′), 128.16 (C-α), 124.93 (C-1), 115.29 (C-6), 114.83 (C-4), 112.12 (C-3), 55.75 (C-2-O*C*H_3_), 55.24 (C-5-O*C*H_3_).

4′-hydroxy-2,5-dimethoxydihydrochalcone (**6a**)

^1^H NMR (600 MHz; Acetone-*d*_6_) δ (ppm): 9.19 (s, 1H, C-4-O*H*), 7.91–7.95 (m, 2H, H-2′, and H-6′), 6.90–6.94 (m, 2H, H-3′, and H-5′), 6.86 (d, 1H, *J* = 8.8 Hz, H-3), 6.83 (d, 1H, *J* = 3.1 Hz, H-6), 6.72 (dd, 1H, *J* = 8.8, 3.1 Hz, H-4), 3.79 (s, 3H, C-2-OC*H*_3_), 3.71 (s, 3H, C-5-OC*H*_3_), 3.15–3.20 (m, 2H, CH_2_-α), 2.90–2.96 (m, 2H, CH_2_-β). ^13^C NMR (151 MHz, Acetone-*d*_6_) δ (ppm): 198.02 (C=O), 162.58 (C-4′), 154.50 (C-5), 152.58 (C-2), 131.62 (C-1′), 131.26 (C-2′ and C-6′), 130.14 (C-1), 115.96 (C-3′ and C-5′), 117.13 (C-6), 112.03 (C-3), 111.95 (C-4), 56.09 (C-2-O*C*H_3_), 55.68 (C-5-O*C*H_3_), 38.84 (C-α) 26.36 (C-β).

4′,5-dihydroxy-2-methoxydihydrochalcone (**6b**)

^1^H NMR (600 MHz; Acetone-*d*_6_) δ (ppm): 9.16 (s, 1H, C-4-O*H*), 7.89–7.96 (m, 2H, H-2′, and H-6′), 7.77 (s, 1H, C-2′-O*H*), 6.88–6.94 (m, 2H, H-3′, and H-5′), 6.77 (d, 1H, *J* = 8.7 Hz, H-3), 6.72 (d, 1H, *J* = 3.0 Hz, H-6), 6.63 (dd, 1H, *J* = 8.7, 3.0 Hz, H-4), 3.76 (s, 3H, C-2-OC*H*_3_), 3.13–3.18 (m, 2H, H-α), 2.86–2.91 (m, 2H, H-β). ^13^C NMR (151 MHz, Acetone-*d*_6_) δ (ppm): 198.03 (C=O), 162.56 (C-4′), 151.90 (C-2), 151.76 (C-5), 131.52 (C-1), 131.26 (C-2′ and C-6′),130.17 (C-1′), 117.91 (C-6), 115.97 (C-3′ and C-5′), 113.76 (C-4), 112.35 (C-3), 56.12 (C-2-O*C*H_3_), 38.92 (C-α) 26.33 (C-β).

*trans*-4′-hydroxy-3,5-dimethoxychalcone (*trans*-**7**)

^1^H NMR (600 MHz; DMSO-*d*_6_) δ (ppm): 10.45 (s, 1H, C-4′-OH), 8.07–8.11 (m, 2H, H-2′, and H-6′), 7.91 (d, 1H, *J* = 15.6 Hz, H-α), 7.60 (d, 1H, *J* = 15.6 Hz, H-β), 7.05 (d, 2H, *J* = 2.2 Hz, H-2, and H-6), 6.87–6.92 (m, 2H, H-3′, and H-5′), 6.57 (d, 1H, *J* = 2.2 Hz, H-4), 3.80 (s, 6H, C3-OC*H*_3_, and C-5-OC*H*_3_). ^13^C NMR (151 MHz, DMSO-*d*_6_) δ (ppm): 187.16 (C=O), 162.27 (C-4′), 160.73 (C-3 and C-5), 142.90 (C-β), 136.86 (C-1), 131.31 (C-2′ and C-6′), 129.08 (C-1′), 122.58 (C-α), 115.39 (C-3′ and C-5′), 106.64 (C-6), 106.58 (C-2), 102.64 (C-4), 55.46 (C-3-O*C*H_3_ and C-5-O*C*H_3_).

*cis*-4′-hydroxy-3,5-dimethoxychalcone (*cis*-**7**)

^1^H NMR (600 MHz; DMSO-*d*_6_) δ (ppm): 10.48 (s, 1H, C-4′-O*H*), 7.79–7.83 (m, 2H, H-2′, and H-6′), 6.84 (d, 1H, *J* = 13.0 Hz, H-β), 6.80–6.85 (m, 2H, H-3′, and H-5′), 6.65 (d, 1H, *J* = 13.0 Hz, H-α), 6.50 (d, 2H, *J* = 2.2 Hz, H-2, and H-6), 6.37 (d, 1H, *J* = 2.2 Hz, H-4), 3.61 (s, 6H, C-3-OC*H*_3_, and C-5-OC*H*_3_). ^13^C NMR (151 MHz, DMSO-*d*_6_) δ (ppm): 193.55 (C=O), 162.58 (C-4′), 160.17 (C-3 and C-5), 137.21 (C-1), 135.63 (C-β), 131.39 (C-2′ and C-6′), 128.88 (C-α), 128.21 (C-1′), 115.58 (C-3′ and C-5′), 107.07 (C-2 and C-6), 100.43 (C-4), 55.12 (C-3-O*C*H_3_ and C-5-O*C*H_3_).

4′-hydroxy-3,5-dimethoxydihydrochalcone (**7a**)

^1^H NMR (600 MHz; Acetone-*d*_6_) δ (ppm): 9.56 (s, 1H, C-4-O*H*), 7.90–7.93 (m, 2H, H-2′, and H-6′), 6.89–6.92 (m, 2H, H-3′, and H-5′), 6.46 (d, 2H, *J* = 2.2 Hz, H-2, and H-6), 6.30 (d, 1H, *J* = 2.2 Hz, H-4), 3.74 (s, 6H, C-3-OC*H*_3_, and C-5-OC*H*_3_) 3.23–3.27 (m, 1H, H-α), 2.90–2.94 (m, 1H, H-β). ^13^C NMR (151 MHz, Acetone-*d*_6_) δ (ppm): 197.82 (C=O), 162.82 (C-4′), 161.87 (C-5 and C-3), 145.02 (C-1), 131.29 (C-2′ and C-6′), 130.04 (C-1′), 116.01 (C-3′ and C-5′), 107.29 (C-6 and C-2), 98.58 (C-4), 55.43 (C-3-O*C*H_3_ and C-5-O*C*H_3_), 40.12 (C-α) 31.26 (C-β).

### 3.3. Microorganisms and Evaluation of Light Influence on the Biotransformation Process

The yeast strain *Yarrowia lipolytica* KCh 71 used in this study originates from the collection of the Department of Food Chemistry and Biocatalysis (KCh) at Wrocław University of Environmental and Life Sciences. The microorganisms were grown in 300 mL Erlenmeyer flasks containing 50 mL of medium (0.5% peptone K, 0.5% Aminobac, and 3% glucose in distilled water) at 25 °C, with continuous shaking at 140 rpm on a rotary shaker. Each experiment was performed in triplicate, and the results are expressed as mean values. The experiments were conducted in three variants:

**Method A**: The trans-chalcones, freshly dissolved in DMSO, were added to the strain culture (resulting in a concentration of 200 µg/mL in the growth medium), and the biotransformation was carried out under natural daylight during daytime hours. During nighttime hours, the cultures remained in the laboratory without any artificial illumination.

**Method B**: The trans-chalcones were dissolved in DMSO and isomerised in sunlight according to a previously described procedure [9]. After isomerisation, the mixture of trans-cis chalcones was added to the *Yarrowia lipolytica* KCh 71 culture to achieve a concentration of 200 µg/mL. The biotransformation was performed in a brightly lit room with access to sunlight.

**Method C**: The trans-chalcones, freshly dissolved in DMSO, were added to the strain culture (resulting in a concentration of 200 µg/mL in the growth medium), and the biotransformation was conducted in complete darkness, wrapped in aluminium foil to prevent light exposure.

At time intervals of 30 min, 1 h, 3 h, and 24 h, the reaction mixtures were extracted with ethyl acetate, dried over anhydrous magnesium sulfate, and analysed by gas chromatography.

### 3.4. Preparative Biotransformations

Preparative biotransformations were performed in 2 L Erlenmeyer flasks, each containing 500 mL of culture medium. The *Yarrowia lipolytica* KCh 71 culture was incubated for three days at 25 °C on a rotary shaker (140 rpm). Subsequently, 100 mg of the substrate dissolved in 2 mL of DMSO was added to the culture. Incubation continued for seven days under standard daylight laboratory conditions to assess the stability of the hydrogenation products and to verify whether any downstream transformations occurred during prolonged cultivation. Products were isolated by triple extraction with ethyl acetate (3 × 300 mL), dried over anhydrous magnesium sulfate, and concentrated in vacuo. Transformation products were separated by preparative TLC and analysed by TLC, GC, and NMR.

## 4. Conclusions

This study demonstrates that *Yarrowia lipolytica* KCh 71 rapidly and selectively hydrogenates the α,β-unsaturated bond of 4′-hydroxychalcones under mild conditions, efficiently converting both *trans* and *cis* isomers and typically reaching ≥90% conversion within 3 h. Light exposure did not measurably affect the course of bioreduction when comparing sunlight-exposed and dark conditions, indicating that the enzymatic step itself is insensitive to light under the tested settings.

The number and position of methoxy substituents on ring B modulated the rate of hydrogenation, with most substrates transforming extremely fast and only selected patterns showing slower conversion within 24 h (notably the 3,5-dimethoxychalcone **7**). Despite these kinetic differences, we established biocatalytic access to all three target dimethoxy dihydrochalcones: 4′-hydroxy-2,4-dimethoxydihydrochalcone (**5a**), 4′-hydroxy-2,5-dimethoxydihydrochalcone (**6a**), and 4′-hydroxy-3,5-dimethoxydihydrochalcone (**7a**), each fully characterised by NMR. To the best of our knowledge, the route to **6a**, **6b**, and **7a** has not been previously reported in a biocatalytic context.

Taking into account various aspects of the process, the approach indicates potential for scale-up, suggesting that larger-scale implementation could be feasible under appropriate conditions. *Yarrowia lipolytica* is an established industrial microorganism authorised as a novel food in the EU. In future work, fed-batch operation, immobilised-cell biocatalysis, or continuous-flow configurations could further increase space–time yields and improve process economics. Crucially, significant product formation was already observed after 30 min, and ≥90% conversion was typically reached within 3 h, underscoring the method’s potential preparative value under intensified conditions.

Under preparative-scale conditions with prolonged incubation (7 days), a minor additional product (≤10%) appeared only in the 2,5-dimethoxy series alongside **6a** and was identified, based on NMR data, as 4′,5-dihydroxy-2-methoxydihydrochalcone (**6b**). This side process was not detected in short-term experiments and can be avoided by appropriate control of reaction time. Collectively, these findings position *Y. lipolytica* KCh 71 as a practical and robust biocatalyst for the concise synthesis of 4′-hydroxydihydrochalcones, delivering fast, high-selectivity reductions across substitution patterns, access to structurally valuable targets **5a**, **6a**, **7a**, and tunability via reaction time control to suppress secondary transformations. Given the structural proximity of the obtained products to natural dihydrochalcones with recognised bioactivities, the workflow offers a sustainable alternative to plant extraction and multi-step chemical synthesis for producing candidates relevant to functional foods and nutraceutical development.

## Figures and Tables

**Table 1:** Substitution of methoxy groups in the B-rings of 4′-hydroxychalcones and dihydrochalcones. R^1^–R^4^ correspond to substituents at positions C-2, C-3, C-4, and C-5, respectively.

*trans*-chalcone *cis*-chalcone	dihydrochalcones	R^1^	R^2^	R^3^	R^4^
*trans*-**1**, *cis*-**1**	**1a**	-H	-H	-H	-H
*trans*-**2**, *cis*-**2**	**2a**	-OCH_3_	-H	-H	-H
*trans*-**3**, *cis*-**3**	**3a**	-H	-OCH_3_	-H	-H
*trans*-**4**, *cis*-**4**	**4a**	-H	-H	-OCH3	-H
*trans*-**5**, *cis*-**5**	**5a**	-OCH_3_	-H	-OCH_3_	-H
*trans*-**6**, *cis*-**6**	**6a**	-OCH_3_	-H	-H	-OCH_3_
*trans*-**7**, *cis*-**7**	**7a**	-H	-OCH_3_	-H	-OCH_3_

**Table 2:** Composition of the chalcone mixture after isomerisation under sunlight.

chalcone	1	2	3	4	5	6	7
*cis*	65.3	36.9	69.1	15.0	11.6	29.3	47.3
*trans*	34.7	63.1	30.9	85.0	88.4	70.7	52.7

## Data Availability

Data is contained within the article and Supplementary Material.

## Supplementary Materials

The following supporting information can be downloaded at: https://www.mdpi.com/article/10.3390/ijms26189027/s1.
