# Supplementary material for: Effect of Sunlight-Induced Isomerisation on the Biotransformation of 4′-Hydroxychalcones by Yarrowia lipolytica KCh 71
Source: Int J Mol Sci. 2025 Sep 16;26(18):9027. doi: 10.3390/ijms26189027 (PMC12470138; doi:10.3390/ijms26189027)
Supplement: Supplementary file 1 [file ijms-26-09027-s001.zip › ijms-3860940-supplementary.pdf]

Supplementary Data

Effect of Sunlight-Induced Isomerisation on the Biotransformation of 4'-Hydroxychalcones by *Yarrowia lipolytica* KCh 71

Paweł Chlipała, Tomasz Janeczko and Marcelina Mazur *

[1] Department of Food Chemistry and Biocatalysis, Wrocław University of Environmental and Life Sciences, Norwida 25, 50-375 Wrocław, Poland: pawel.chlipala@upwr.edu.pl (P.C.); tomasz.janeczko@upwr.edu.pl (T.J.)

* Correspondence: marcelina.mazur@upwr.edu.pl (MM); Tel.: +48-713205197

Contents:

Figure S1. UV spectrum of *trans*-4'-hydroxychalcone (*trans*-**1**) 3
Figure S2. ¹H NMR spectrum of *trans*-4'-hydroxychalcone (*trans*-**1**) 3
Figure S3. ¹³C NMR spectrum of *trans*-4'-hydroxychalcone (*trans*-**1**) 4
Figure S4. UV spectrum of *cis*-4'-hydroxychalcone (*cis*-**1**) 4
Figure S5. ¹H NMR spectrum of *cis*-4'-hydroxychalcone (*cis*-**1**) 5
Figure S6. ¹³C NMR spectrum of *cis*-4'-hydroxychalcone (*cis*-**1**) 5
Figure S7. UV spectrum of 4'-hydroxydihydrochalcone (**1a**) 6
Figure S8. ¹H NMR spectrum of 4'-hydroxydihydrochalcone (**1a**) 6
Figure S9. ¹³C NMR spectrum of 4'-hydroxydihydrochalcone (**1a**) 7
Figure S10. UV spectrum of *trans*-4'-hydroxy-2-methoxychalcone *(trans*-**2***)* 7
Figure S11. ¹H NMR spectrum of *trans*-4'-hydroxy-2-methoxychalcone *(trans*-**2***)* 8
Figure S12. ¹³C NMR spectrum of *trans*-4'-hydroxy-2-methoxychalcone *(trans*-**2***)* 8
Figure S13. UV spectrum of *cis*-4'-hydroxy-2-methoxychalcone *(cis*-**2***)* 9
Figure S14. ¹H NMR spectrum of *cis*-4'-hydroxy-2-methoxychalcone *(cis*-**2***)* 9
Figure S15. ¹³C NMR spectrum of *cis*-4'-hydroxy-2-methoxychalcone *(cis*-**2***)* 10
Figure S16 UV spectrum of 4'-hydroxy-2-methoxydihydrochalcone (**2a**) 10
Figure S17. ¹H NMR spectrum of 4'-hydroxy-2-methoxydihydrochalcone (**2a**) 11
Figure S18. ¹³C NMR spectrum of 4'-hydroxy-2-methoxydihydrochalcone (**2a**) 11
Figure S19. UV spectrum of *trans*-4'-hydroxy-3-methoxychalkone (*trans*-**3**) 12
Figure S20. ¹H NMR spectrum of *trans*-4'-hydroxy-3-methoxychalkone (*trans*-**3**) 12
Figure S21. ¹³C NMR spectrum of *trans*-4'-hydroxy-3-methoxychalkone (*trans*-**3**) 13
Figure S22. UV spectrum of *cis*-4'-hydroxy-3-methoxychalkone (*cis*-**3**) 13
Figure S23. ¹H NMR spectrum of *cis*-4'-hydroxy-3-methoxychalkone (*cis*-**3**) 14
Figure S24. ¹³C NMR spectrum of *cis*-4'-hydroxy-3-methoxychalkone (*cis*-**3**) 14
Figure S25. UV spectrum of 4'-hydroxy-3-methoxydihydrochalcone (**3a**) 15
Figure S26. ¹H NMR spectrum of 4'-hydroxy-3-methoxydihydrochalcone (**3a**) 15
Figure S27. ¹³C NMR spectrum of 4'-hydroxy-3-methoxydihydrochalcone (**3a**) 16
Figure S28. UV spectrum of *trans*-4'-hydroxy-4-methoxychalcone (*trans*-**4**) 16
Figure S29. ¹H NMR spectrum of *trans*-4'-hydroxy-4-methoxychalcone (*trans*-**4**) 17
Figure S30. ¹³C NMR spectrum of *trans*-4'-hydroxy-4-methoxychalcone (*trans*-**4**) 17
Figure S31. UV spectrum of *cis*-4'-hydroxy-4-methoxychalcone (*cis*-**4**) 18
Figure S32. ¹H NMR spectrum of *cis*-4'-hydroxy-4-methoxychalcone (*cis*-**4**) 18
Figure S33. ¹³C NMR spectrum of *cis*-4'-hydroxy-4-methoxychalcone (*cis*-**4**) 19
Figure S34. UV spectrum of 4'-hydroxy-4-methoxydihydrochalcone (**4a**) 19
Figure S35. ¹H NMR spectrum of 4'-hydroxy-4-methoxydihydrochalcone (**4a**) 20
Figure S36. ¹³C NMR spectrum of 4'-hydroxy-4-methoxydihydrochalcone (**4a**) 20
Figure S37. UV spectrum of *trans*-4′-hydroxy-2,4-dimethoxychalcone (*trans*-**5**) 21
Figure S38. ¹H NMR spectrum of *trans*-4′-hydroxy-2,4-dimethoxychalcone (*trans*-**5**) 21
Figure S39. Part of the ¹H NMR spectrum of *trans*-4′-hydroxy-2,4-dimethoxychalcone (*trans*-**5**) 22
Figure S40. ¹³C NMR spectrum of *trans*-4′-hydroxy-2,4-dimethoxychalcone (*trans*-**5**) 22
Figure S41. COSY NMR spectrum of *trans*-4′-hydroxy-2,4-dimethoxychalcone (*trans*-**5**) 23
Figure S42. HSQC NMR spectrum of *trans*-4′-hydroxy-2,4-dimethoxychalcone (*trans*-**5**) 23
Figure S43. HMBC NMR spectrum of *trans*-4′-hydroxy-2,4-dimethoxychalcone (*trans*-**5**) 24
Figure S44. UV spectrum of *cis*-4′-hydroxy-2,4-dimethoxychalcone (*cis*-**5**) 24

Figure S45. ^{1}H NMR spectrum of *cis*-4′-hydroxy-2,4-dimethoxychalcone (*cis*-**5**) 25
Figure S46. ^{13}C NMR spectrum of *cis*-4′-hydroxy-2,4-dimethoxychalcone (*cis*-**5**) 25
Figure S47. COSY spectrum of *cis*-4′-hydroxy-2,4-dimethoxychalcone (*cis*-**5**) 26
Figure S48. HSQC NMR spectrum of *cis*-4′-hydroxy-2,4-dimethoxychalcone (*cis*-**5**) 26
Figure S49. HMBC NMR spectrum of *cis*-4′-hydroxy-2,4-dimethoxychalcone (*cis*-**5**) 27
Figure S50. UV spectrum of 4’-hydroxy-2,4-dimetoxydihydrochalcone (**5a**) 27
Figure S51. ^{1}H NMR spectrum of 4’-hydroxy-2,4-dimetoxydihydrochalcone (**5a**) 28
Figure S52. ^{13}C NMR spectrum of 4’-hydroxy-2,4-dimetoxydihydrochalcone (**5a**) 28
Figure S53. COSY NMR spectrum of 4’-hydroxy-2,4-dimetoxydihydrochalcone (**5a**) 29
Figure S54. HSQC NMR spectrum of 4’-hydroxy-2,4-dimetoxydihydrochalcone (**5a**) 29
Figure S55. HMBC NMR spectrum of 4’-hydroxy-2,4-dimetoxydihydrochalcone (**5a**) 30
Figure S56. UV spectrum of *trans*-4′ -hydroxy-2,5-dimethoxychalcone (*trans*-**6**) 30
Figure S57. ^{1}H NMR spectrum of *trans*-4′ -hydroxy-2,5-dimethoxychalcone (*trans*-**6**) 31
Figure S58. ^{13}C NMR spectrum of *trans*-4′ -hydroxy-2,5-dimethoxychalcone (*trans*-**6**) 31
Figure S59. COSY NMR spectrum of *trans*-4′ -hydroxy-2,5-dimethoxychalcone (*trans*-**6**) 32
Figure S60. HSQC NMR spectrum of *trans*-4′ -hydroxy-2,5-dimethoxychalcone (*trans*-**6**) 32
Figure S61. HMBC NMR spectrum of *trans*-4′ -hydroxy-2,5-dimethoxychalcone (*trans*-**6**) 33
Figure S62. UV spectrum of *cis*-4′ -hydroxy-2,5-dimethoxychalcone (*cis*-**6**) 33
Figure S63. ^{1}H NMR spectrum of *cis*-4′ -hydroxy-2,5-dimethoxychalcone (*cis*-**6**) 34
Figure S64. ^{13}C NMR spectrum of *cis*-4′ -hydroxy-2,5-dimethoxychalcone (*cis*-**6**) 34
Figure S65. COSY NMR spectrum of *cis*-4′ -hydroxy-2,5-dimethoxychalcone (*cis*-**6**) 35
Figure S66. HSQC NMR spectrum of *cis*-4′ -hydroxy-2,5-dimethoxychalcone (*cis*-**6**) 35
Figure S67. HMBC NMR spectrum of *cis*-4′ -hydroxy-2,5-dimethoxychalcone (*cis*-**6**) 36
Figure S68. UV spectrum of 4’-hydroxy-2,5-dimethoxydihydrochalcone (**6a**) 36
Figure S69. ^{1}H NMR spectrum of 4’-hydroxy-2,5-dimethoxydihydrochalcone (**6a**) 37
Figure S70. ^{13}C NMR spectrum of 4’-hydroxy-2,5-dimethoxydihydrochalcone (**6a**) 37
Figure S71. COSY NMR spectrum of 4’-hydroxy-2,5-dimethoxydihydrochalcone (**6a**) 38
Figure S72. HSQC NMR spectrum of 4’-hydroxy-2,5-dimethoxydihydrochalcone (**6a**) 38
Figure S73. HMBC NMR spectrum of 4’-hydroxy-2,5-dimethoxydihydrochalcone (**6a**) 39
Figure S74. UV spectrum of 4’,5-dihydroxy-2-methoxydihydrochalcone (**6b**) 39
Figure S75. ^{1}H NMR spectrum of 4’,5-dihydroxy-2-methoxydihydrochalcone (**6b**) 40
Figure S76. ^{13}C NMR spectrum of 4’,5-dihydroxy-2-methoxydihydrochalcone (**6b**) 40
Figure S77. COSY NMR spectrum of 4’,5-dihydroxy-2-methoxydihydrochalcone (**6b**) 41
Figure S78. HMQC NMR spectrum of 4’,5-dihydroxy-2-methoxydihydrochalcone (**6b**) 41
Figure S79. HMBC NMR spectrum of 4’,5-dihydroxy-2-methoxydihydrochalcone (**6b**) 42
Figure S80 UV spectrum of *trans*-4′-hydroxy-3,5-dimethoxychalcone (*trans*-**7**) 42
Figure S81. ^{1}H NMR spectrum of *trans*-4′-hydroxy-3,5-dimethoxychalcone (*trans*-**7**) 43
Figure S82. ^{13}C NMR spectrum of *trans*-4′-hydroxy-3,5-dimethoxychalcone (*trans*-**7**) 43
Figure S83. COSY NMR spectrum of *trans*-4′-hydroxy-3,5-dimethoxychalcone (*trans*-**7**) 44
Figure S84. HSQC NMR spectrum of *trans*-4′-hydroxy-3,5-dimethoxychalcone (*trans*-**7**) 44
Figure S85. HMBC NMR spectrum of *trans*-4′-hydroxy-3,5-dimethoxychalcone (*trans*-**7**) 45
Figure S86. UV spectrum of *cis*-4′-hydroxy-3,5-dimethoxychalcone (*cis*-**7**) 45
Figure S87. ^{1}H NMR spectrum of *cis*-4′-hydroxy-3,5-dimethoxychalcone (*cis*-**7**) 46
Figure S88. ^{13}C NMR spectrum of *cis*-4′-hydroxy-3,5-dimethoxychalcone (*cis*-**7**) 46
Figure S89. COSY NMR spectrum of *cis*-4′-hydroxy-3,5-dimethoxychalcone (*cis*-**7**) 47
Figure S90. HSQC NMR spectrum of *cis*-4′-hydroxy-3,5-dimethoxychalcone (*cis*-**7**) 47
Figure S91. HMBC NMR spectrum of *cis*-4′-hydroxy-3,5-dimethoxychalcone (*cis*-**7**) 48
Figure S92. UV spectrum of 4’-hydroxy-3,5-dimethoxydihydrochalcone (**7a**) 48
Figure S93. ^{1}H NMR spectrum of 4’-hydroxy-3,5-dimethoxydihydrochalcone (**7a**) 49
Figure S94. ^{13}C NMR spectrum of 4’-hydroxy-3,5-dimethoxydihydrochalcone (**7a**) 49
Figure S95. COSY NMR spectrum of 4’-hydroxy-3,5-dimethoxydihydrochalcone (**7a**) 50
Figure S96. HSQC NMR spectrum of 4’-hydroxy-3,5-dimethoxydihydrochalcone (**7a**) 50
Figure S97. HMBC NMR spectrum of 4’-hydroxy-3,5-dimethoxydihydrochalcone (**7a**) 51

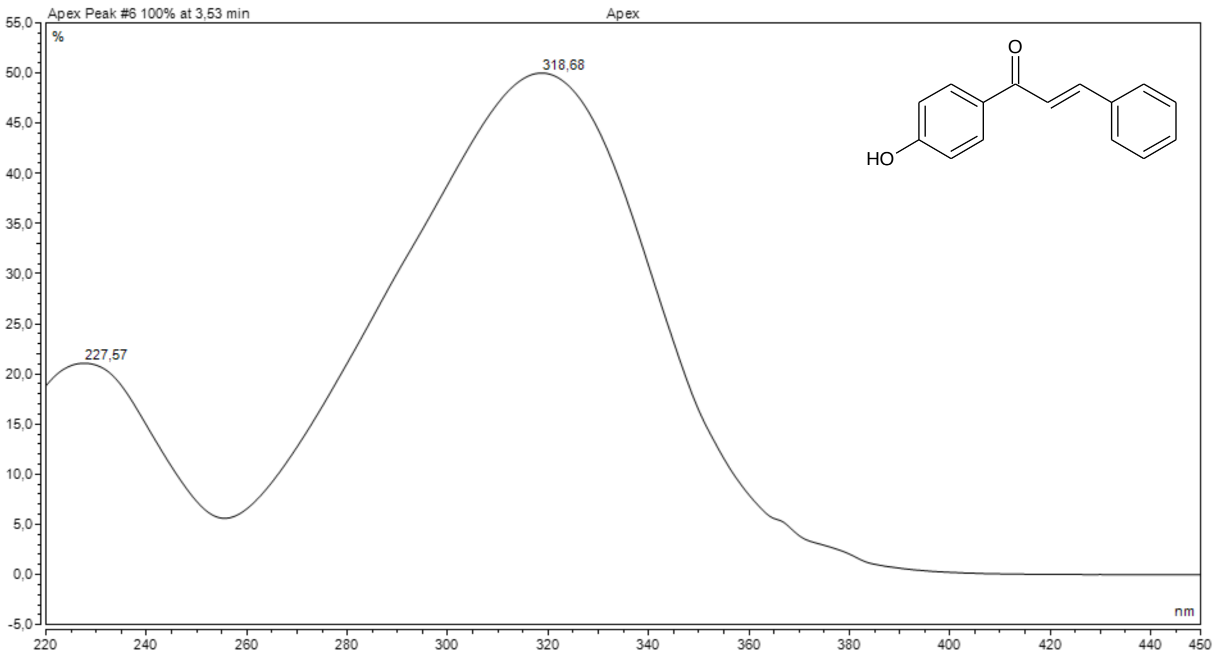

Figure S1. UV spectrum of *trans*-4′-hydroxychalcone (*trans*-**1**)

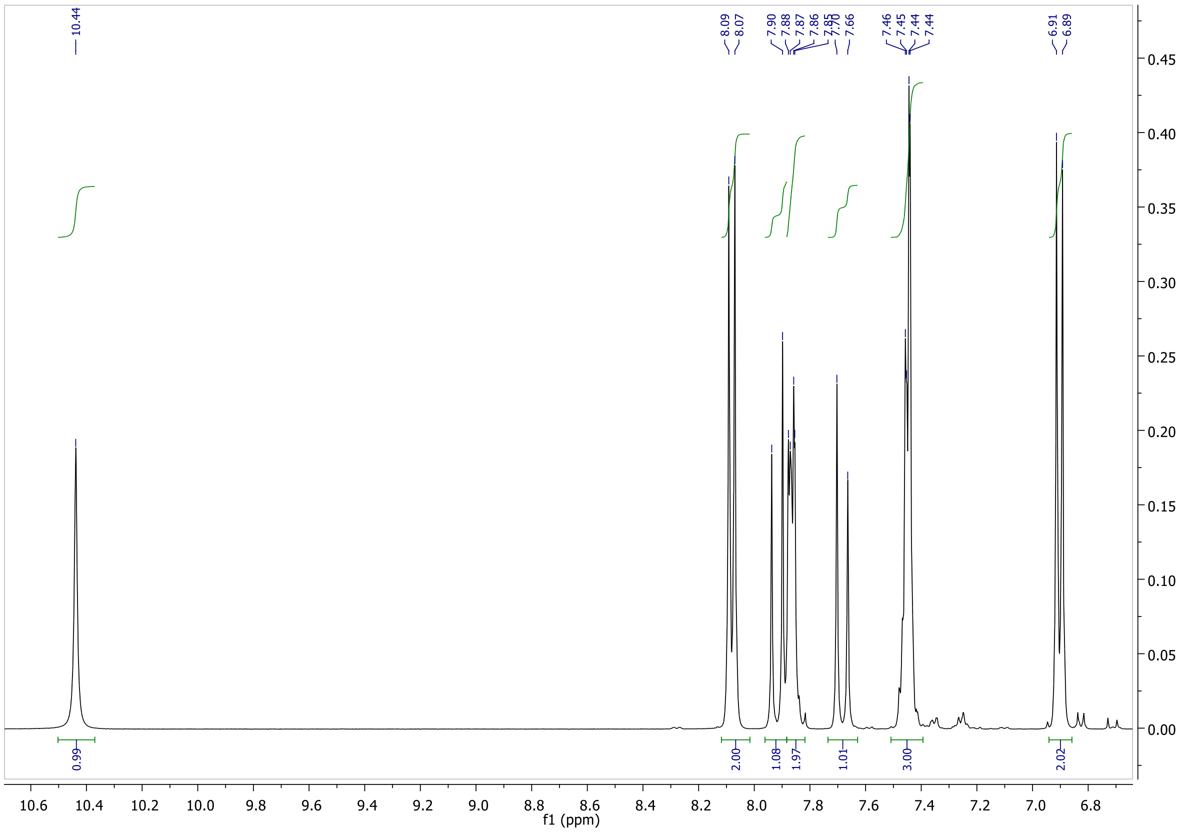

Figure S2. ^{1}H NMR spectrum of *trans*-4′-hydroxychalcone (*trans*-**1**)

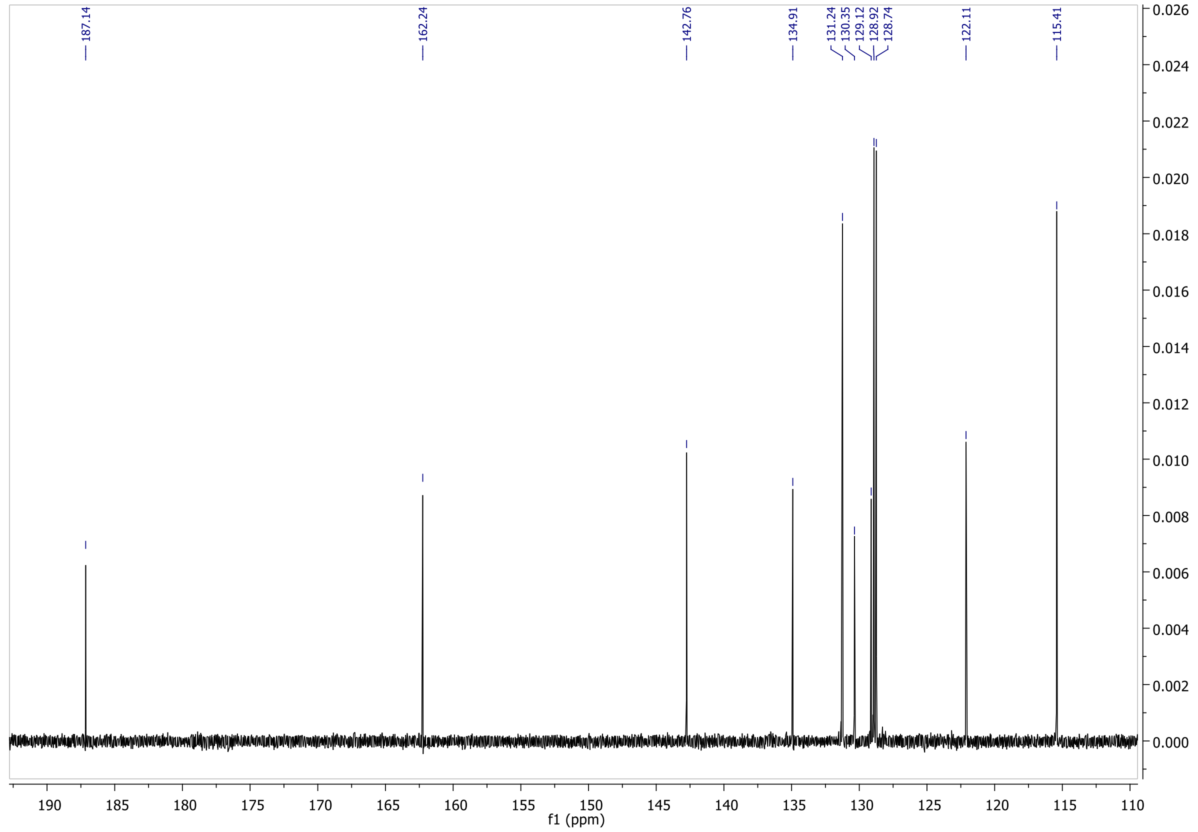

Figure S3. ^{13}C NMR spectrum of *trans*-4′-hydroxychalcone (*trans*-**1**)

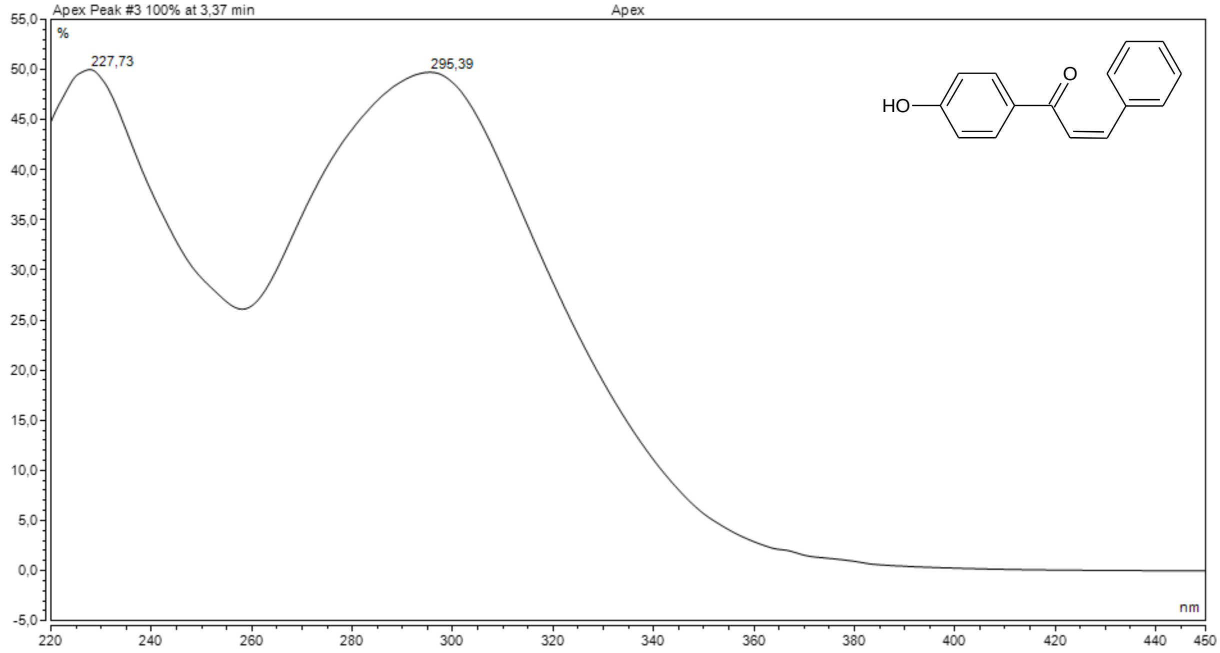

Figure S4. UV spectrum of *cis*-4′-hydroxychalcone (*cis*-**1**)

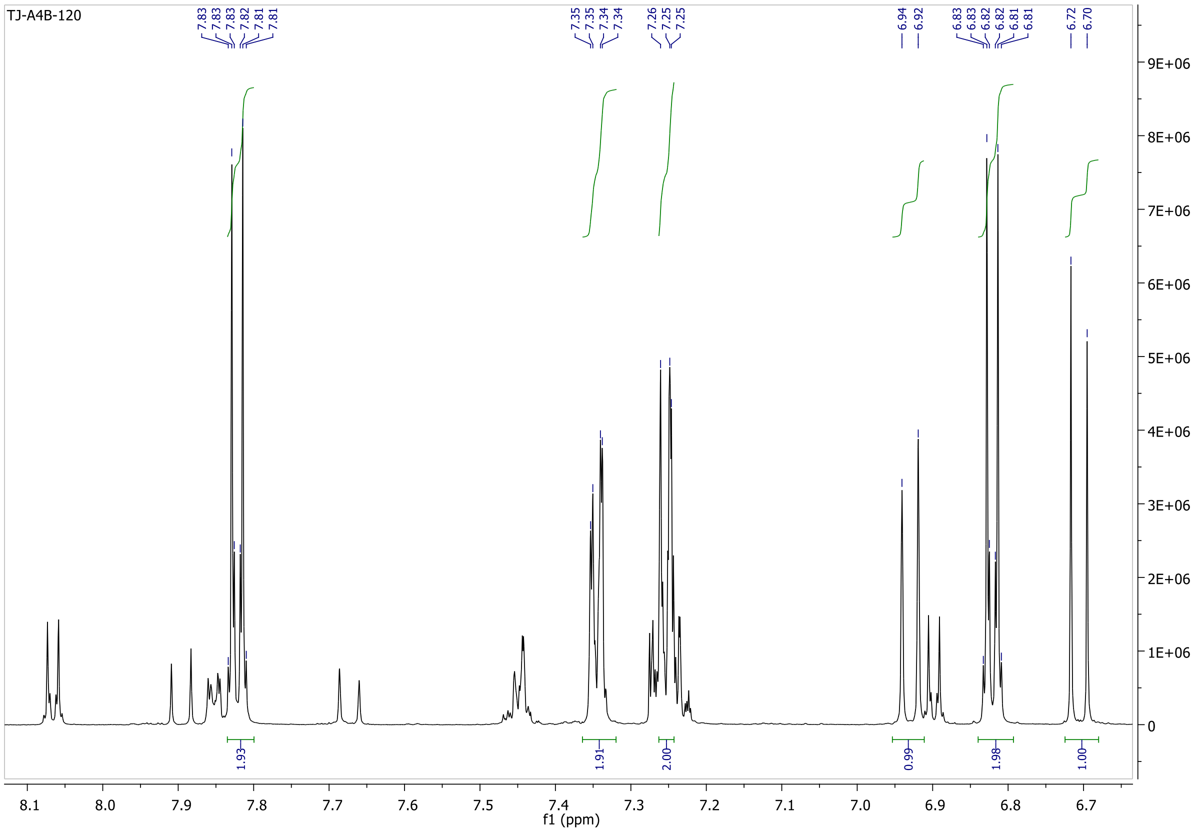

Figure S5. ^{1}H NMR spectrum of *cis*-4′-hydroxychalcone (*cis*-**1**)

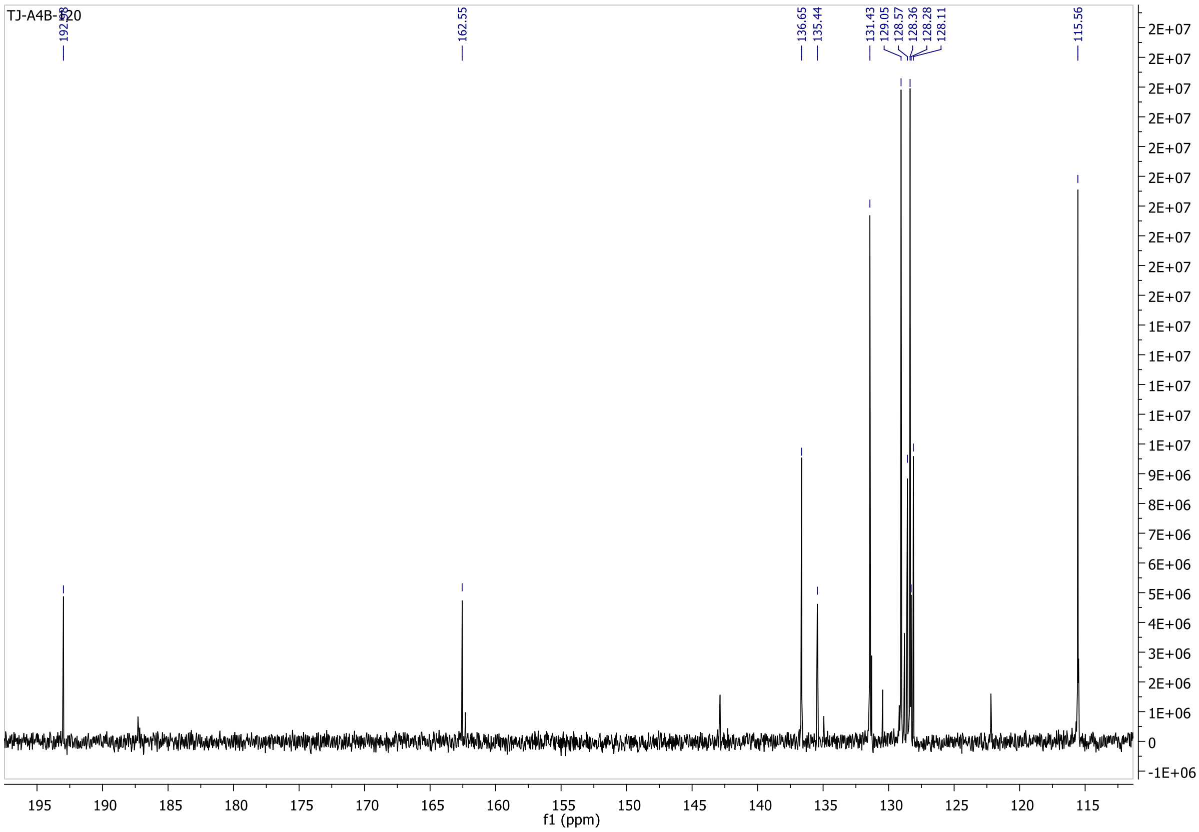

Figure S6. ^{13}C NMR spectrum of *cis*-4′-hydroxychalcone (*cis*-**1**)

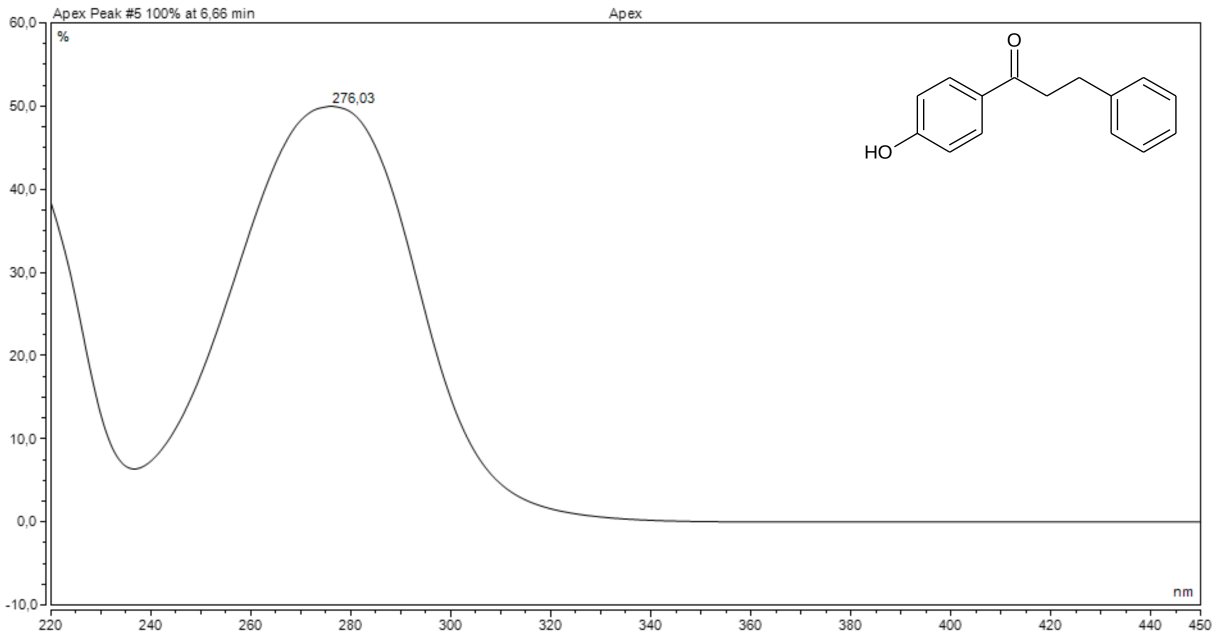

Figure S7. UV spectrum of 4′-hydroxydihydrochalcone (**1a**)

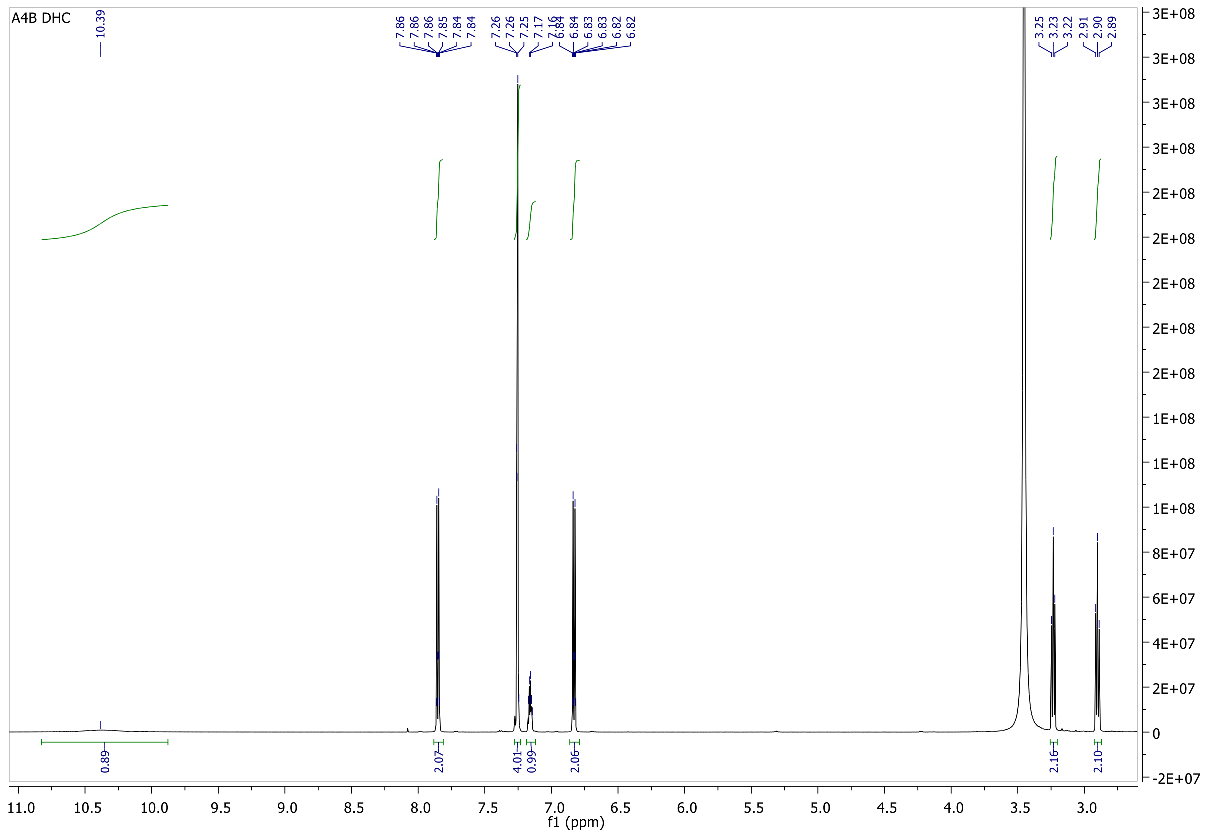

Figure S8. ^{1}H NMR spectrum of 4′-hydroxydihydrochalcone (**1a**)

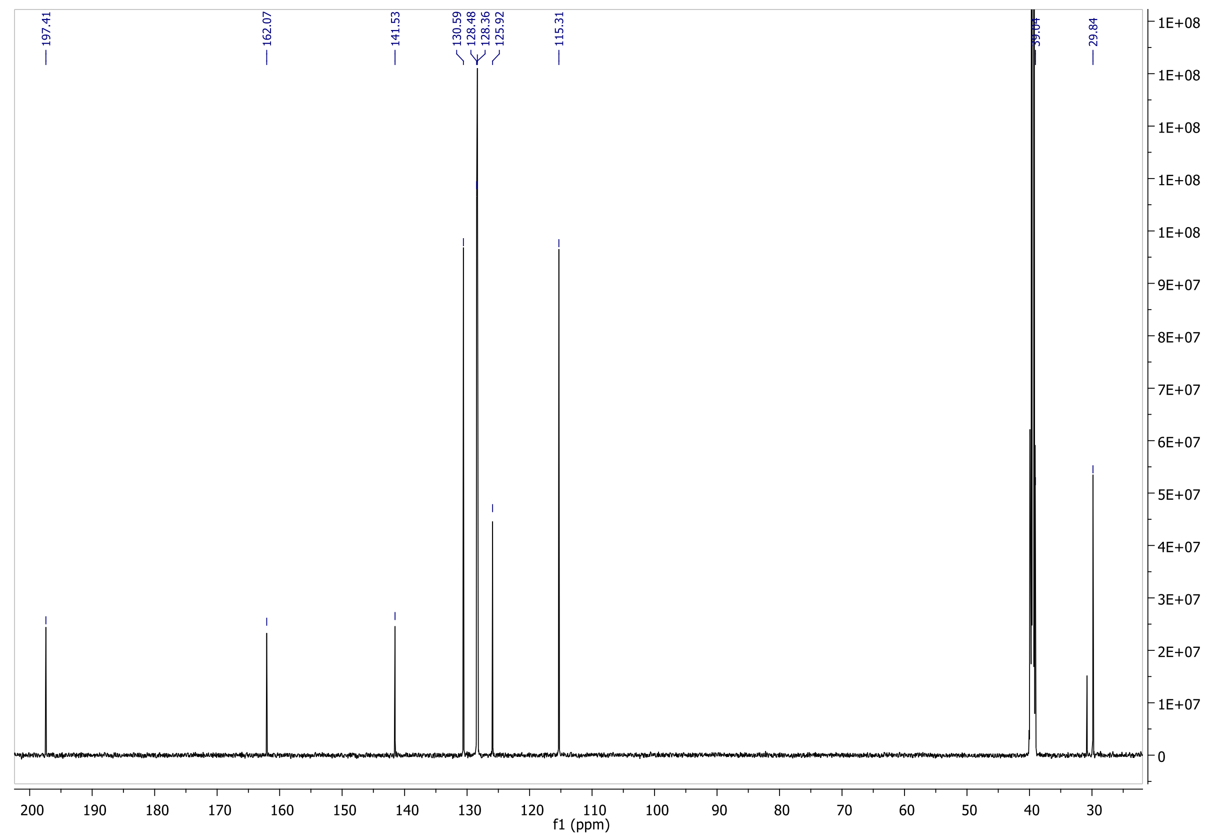

Figure S9. ^{13}C NMR spectrum of 4′-hydroxydihydrochalcone (**1a**)

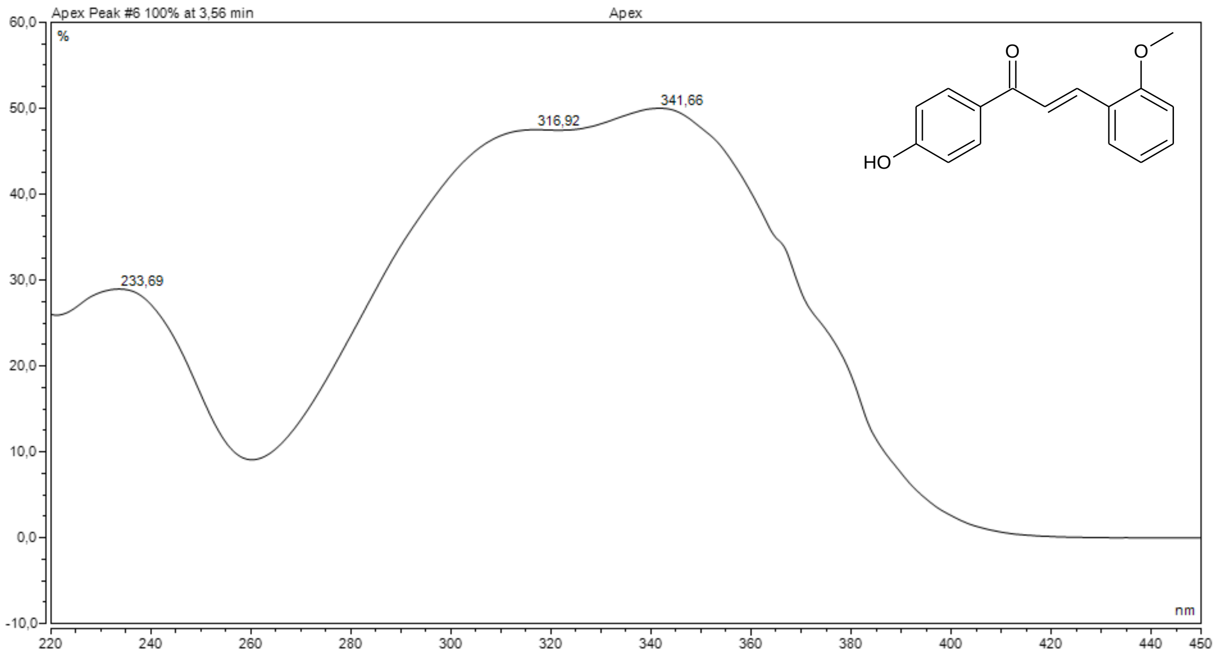

Figure S10. UV spectrum of *trans*-4′-hydroxy-2-methoxychalcone (*trans*-**2**)

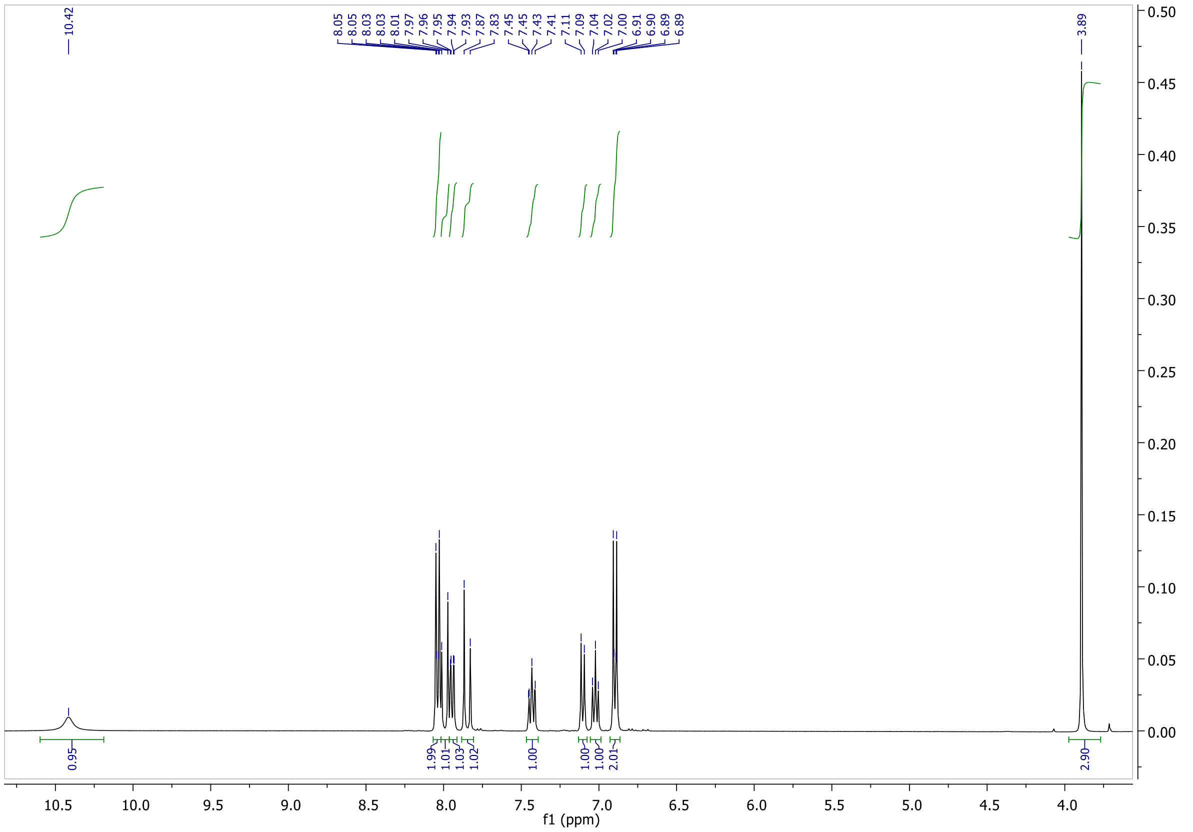

Figure S11. ^{1}H NMR spectrum of *trans*-4′-hydroxy-2-methoxychalcone (*trans*-**2**)

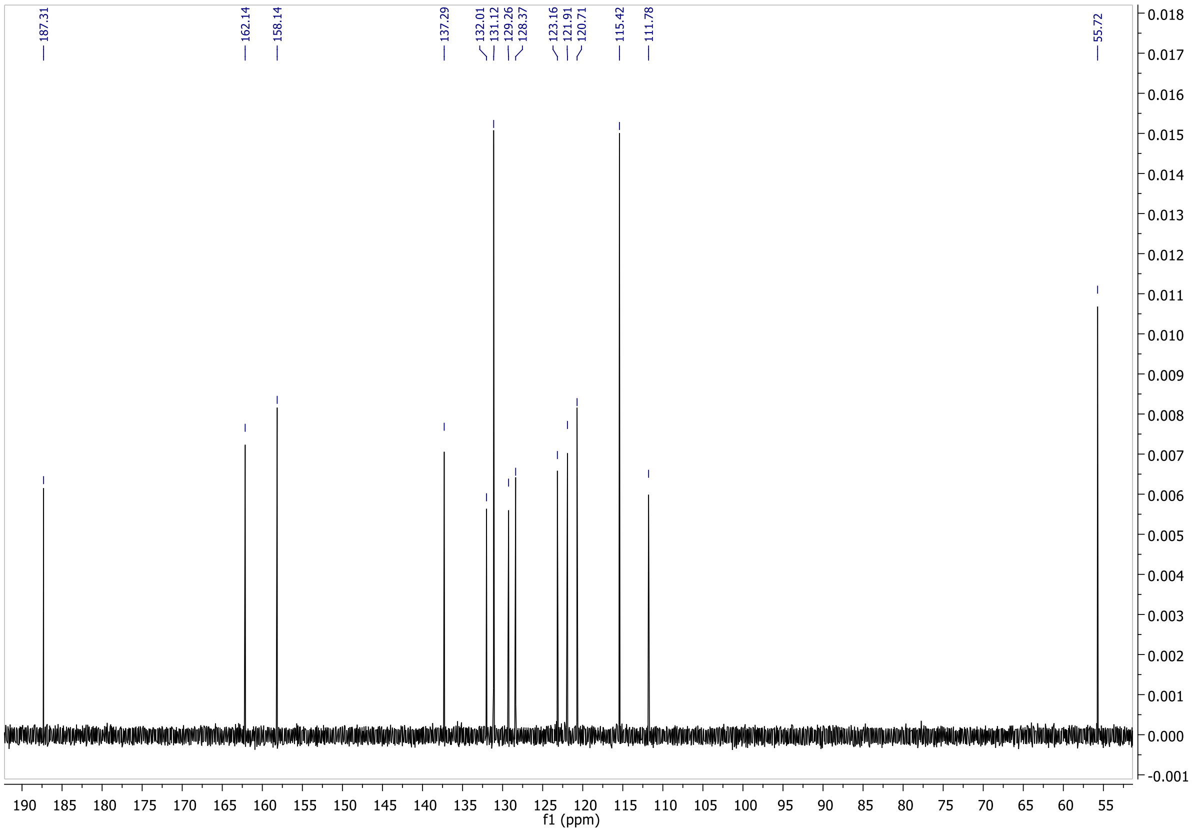

Figure S12. ^{13}C NMR spectrum of *trans*-4′-hydroxy-2-methoxychalcone (*trans*-**2**)

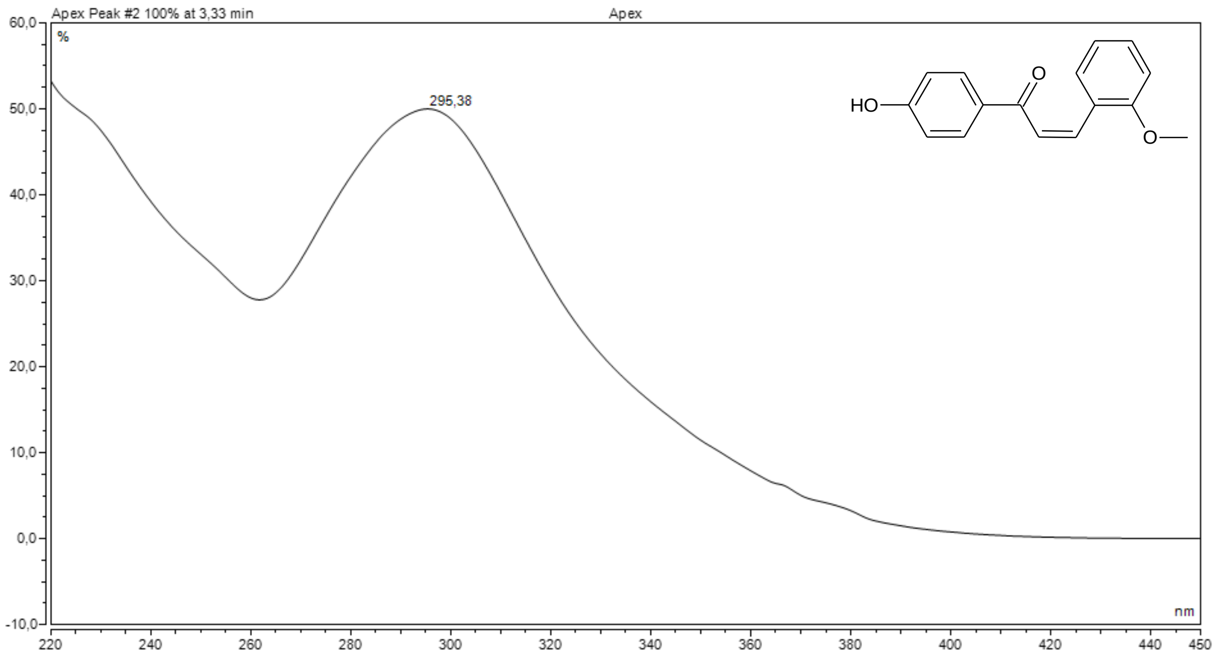

Figure S13. UV spectrum of *cis*-4′-hydroxy-2-methoxychalcone (*cis*-**2**)

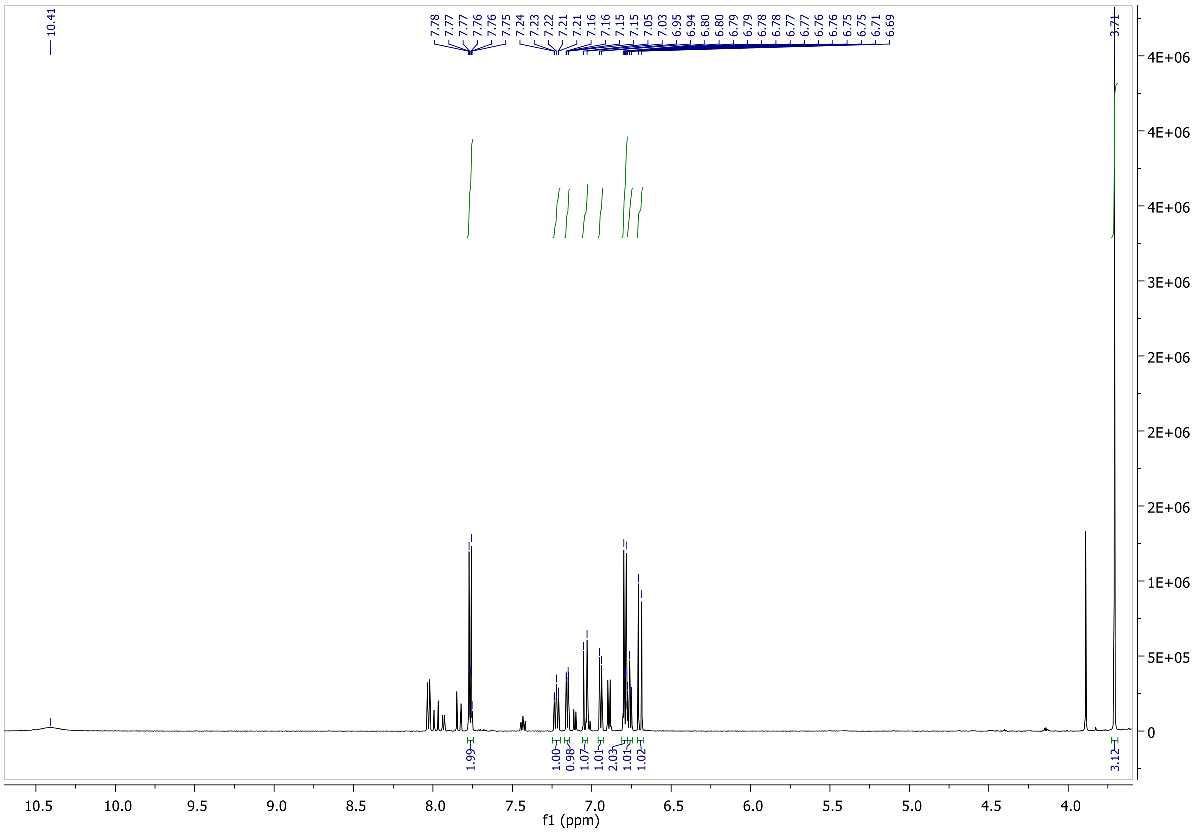

Figure S14. ^{1}H NMR spectrum of *cis*-4′-hydroxy-2-methoxychalcone (*cis*-**2**)

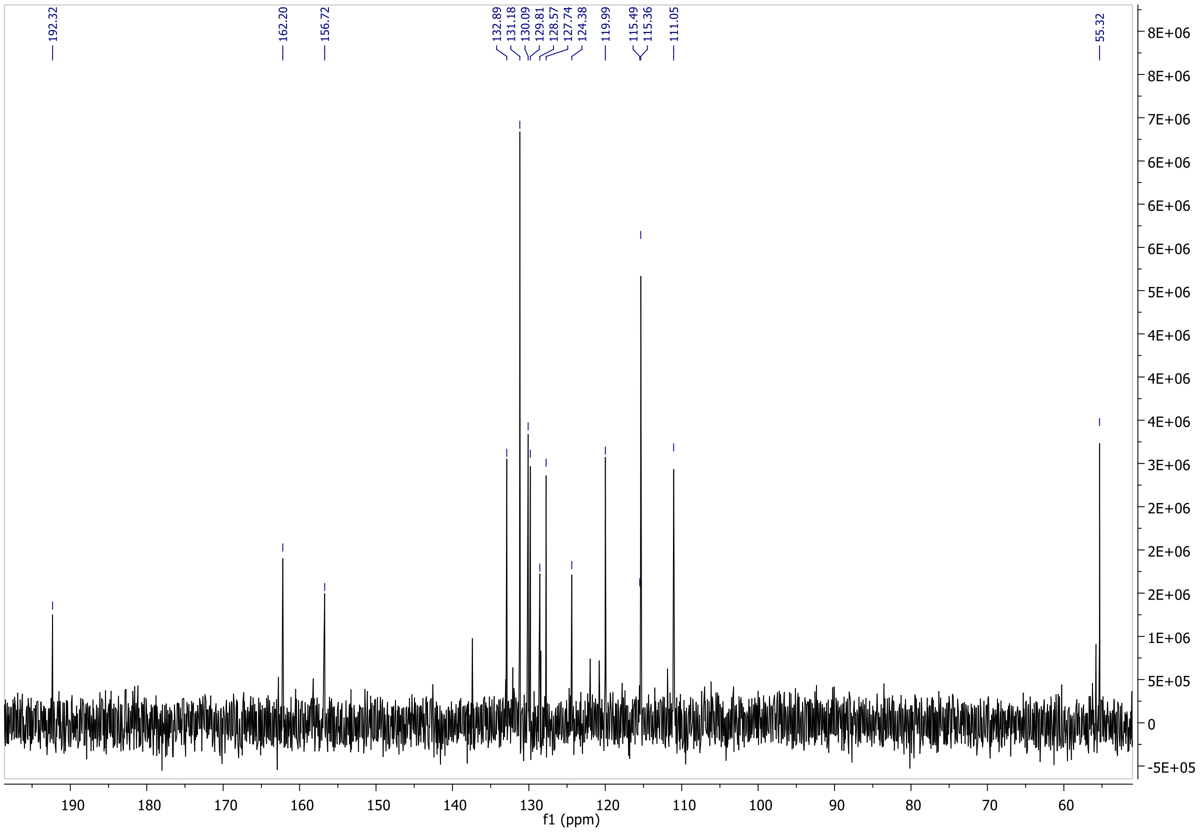

Figure S15. ^{13}C NMR spectrum of *cis*-4′-hydroxy-2-methoxychalcone *(cis*-**2***)*

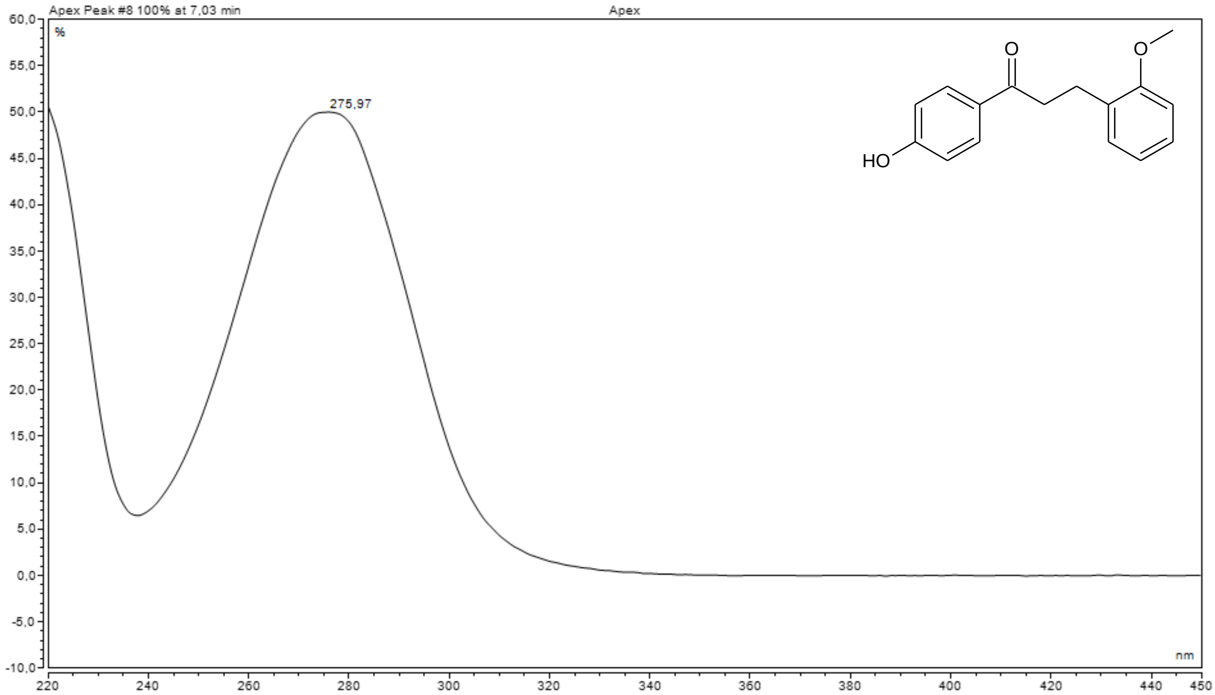

Figure S16 UV spectrum of 4′-hydroxy-2-methoxydihydrochalcone (**2a**)

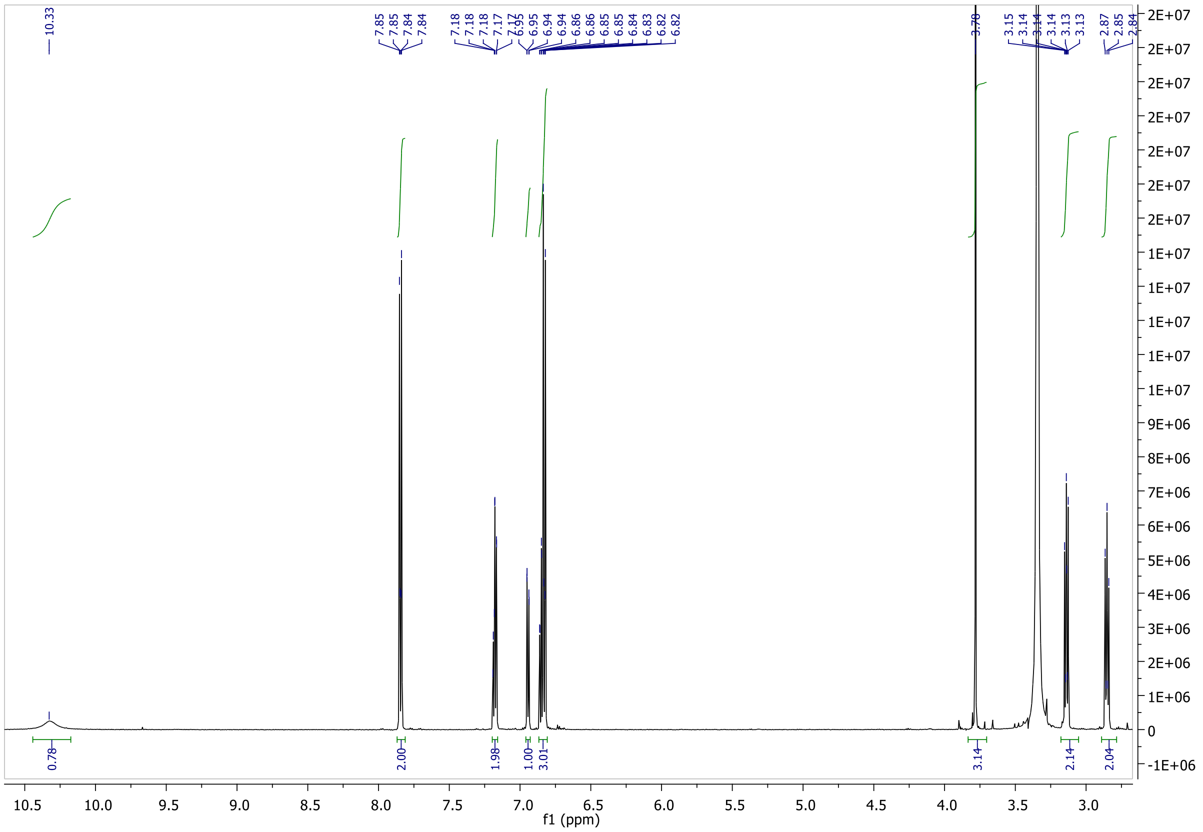

Figure S17. ^{1}H NMR spectrum of 4′-hydroxy-2-methoxydihydrochalcone (**2a**)

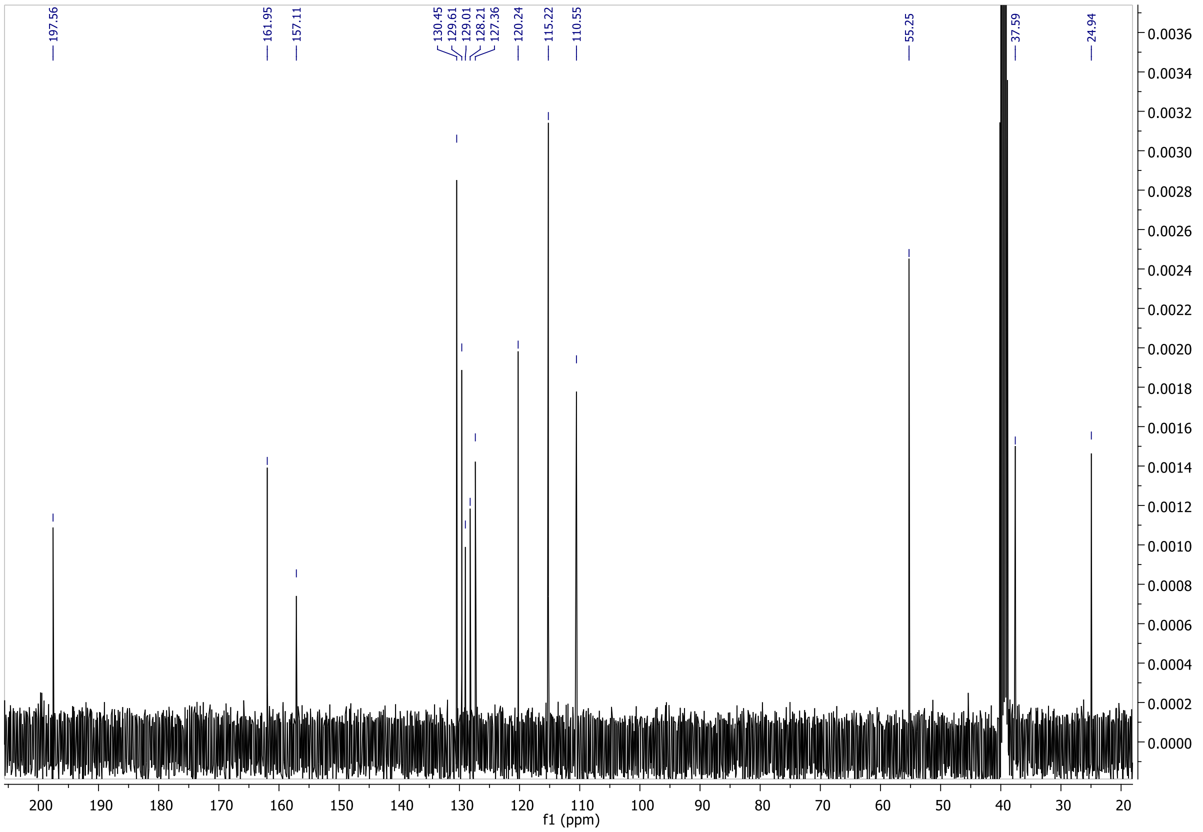

Figure S18. ^{13}C NMR spectrum of 4′-hydroxy-2-methoxydihydrochalcone (**2a**)

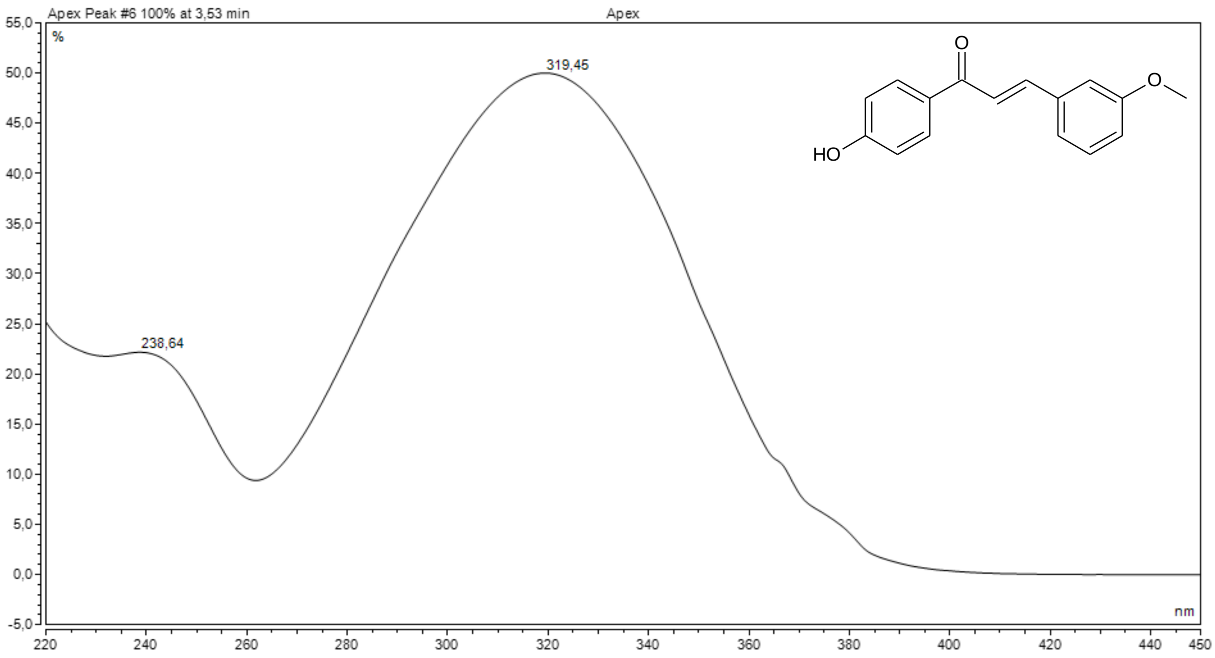

Figure S19. UV spectrum of *trans*-4′-hydroxy-3-methoxychalkone (*trans*-**3**)

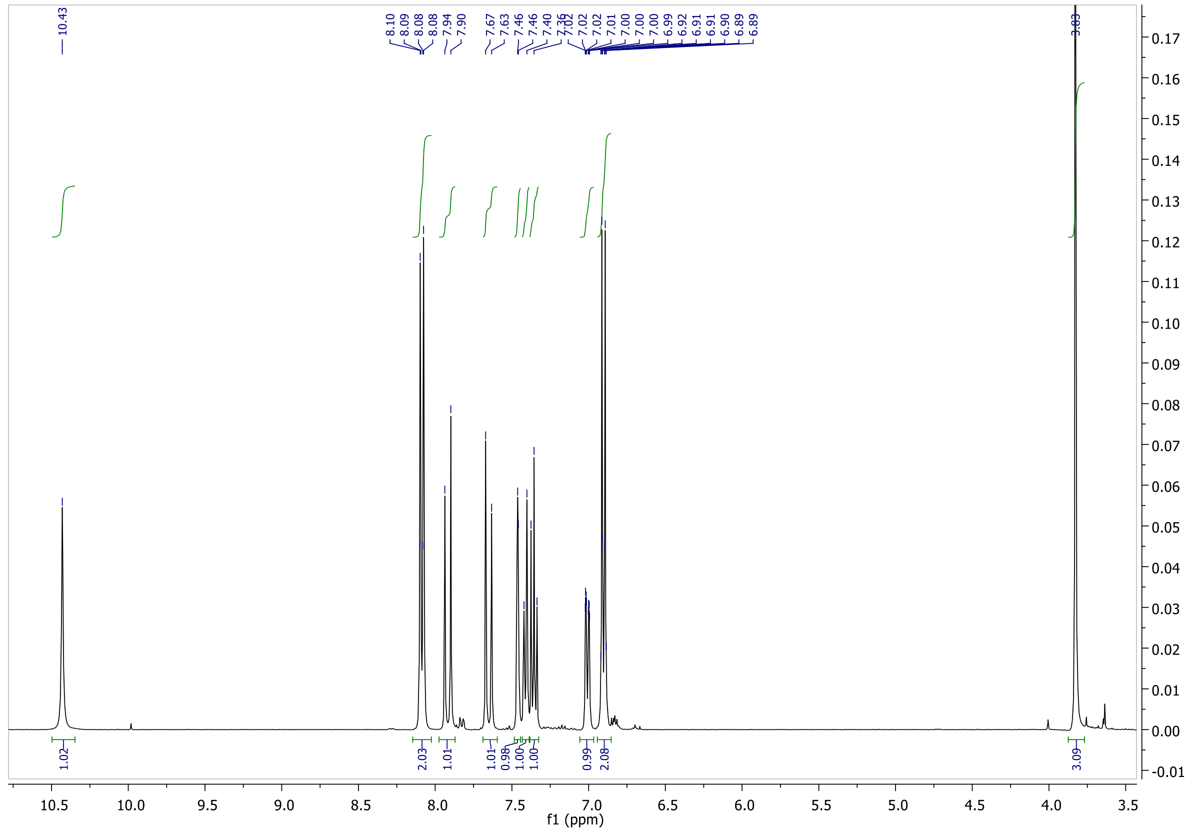

Figure S20. ^{1}H NMR spectrum of *trans*-4′-hydroxy-3-methoxychalkone (*trans*-**3**)

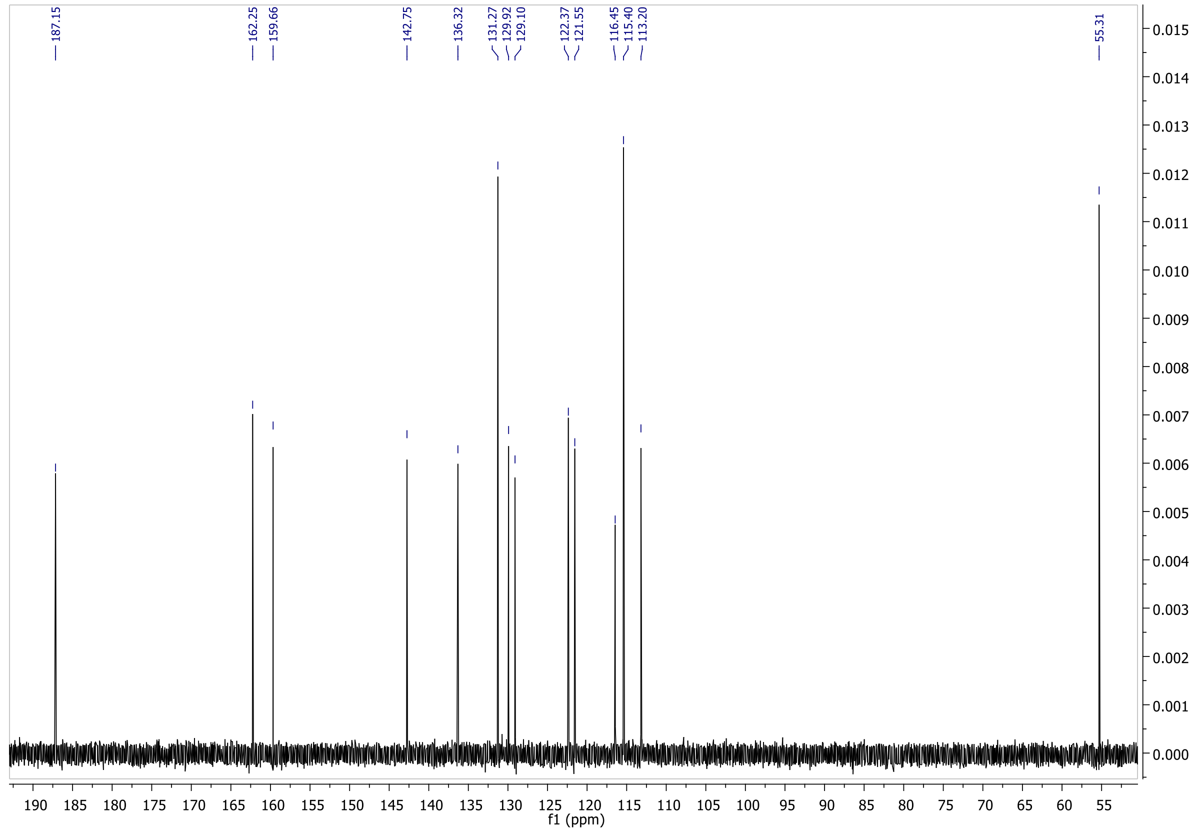

Figure S21. ^{13}C NMR spectrum of *trans*-4′-hydroxy-3-methoxychalkone (*trans*-**3**)

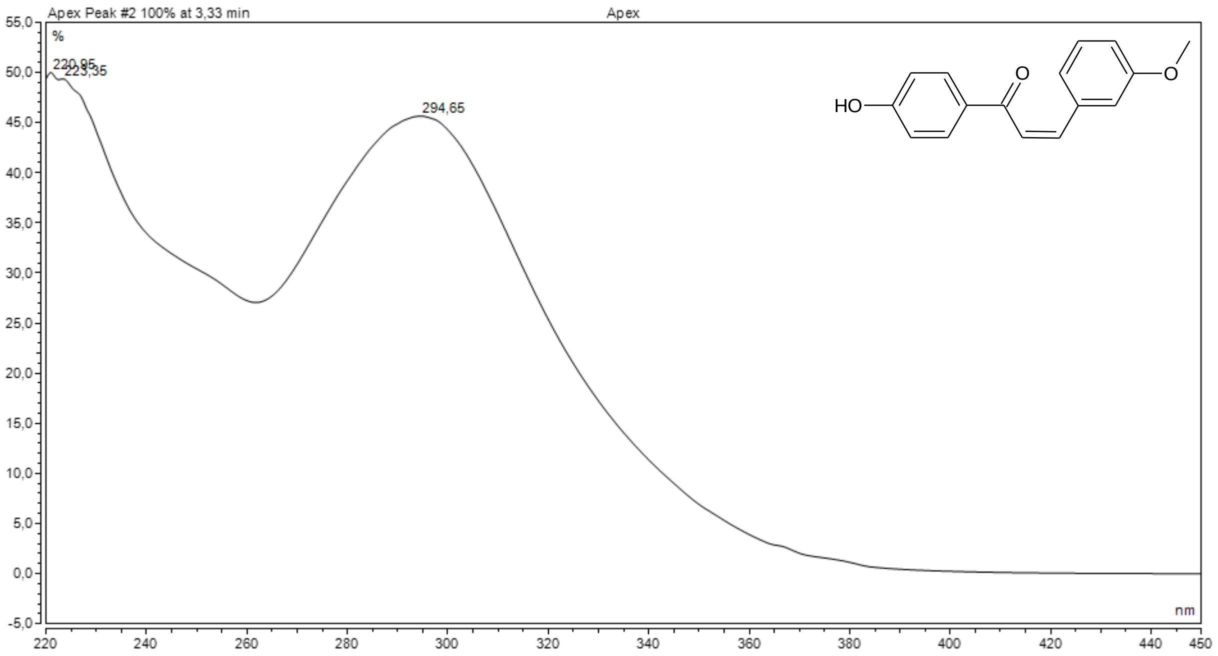

Figure S22. UV spectrum of *cis*-4′-hydroxy-3-methoxychalkone (*cis*-**3**)

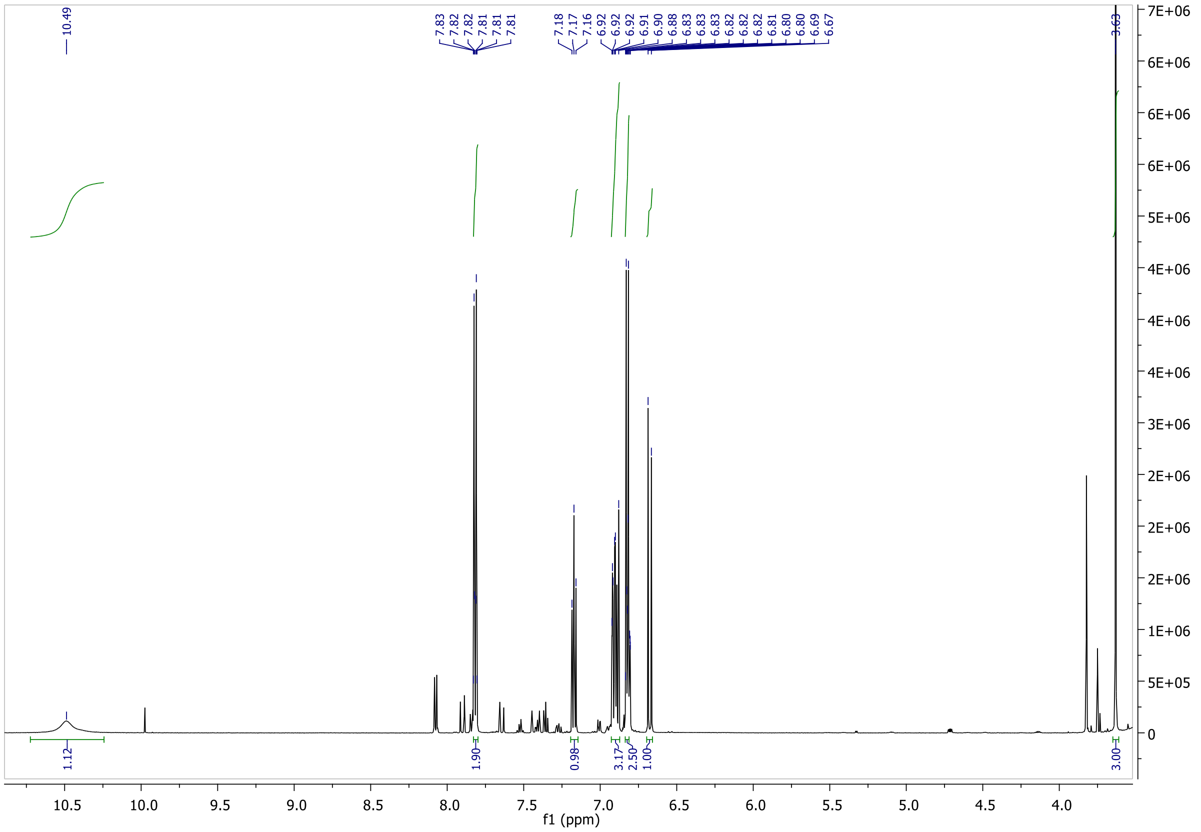

Figure S23. ¹H NMR spectrum of *cis*-4′-hydroxy-3-methoxychalkone (*cis*-**3**)

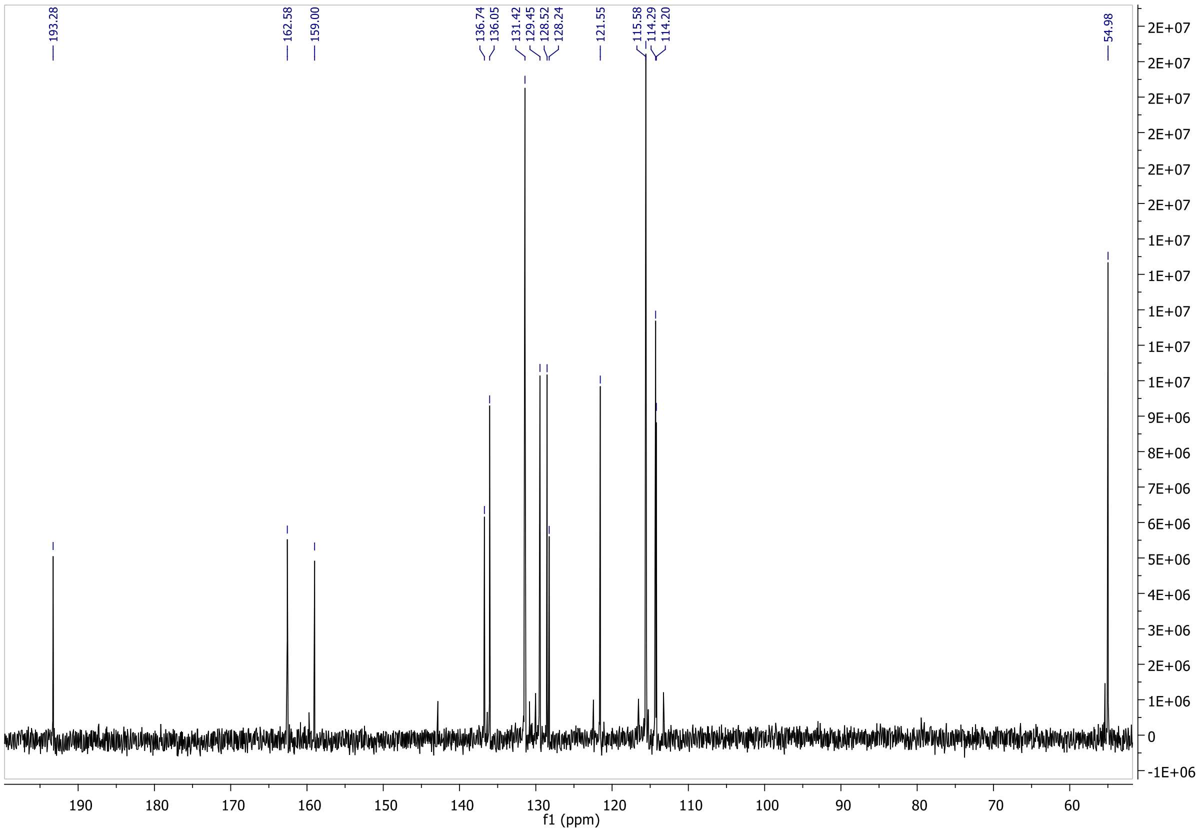

Figure S24. ¹³C NMR spectrum of *cis*-4′-hydroxy-3-methoxychalkone (*cis*-**3**)

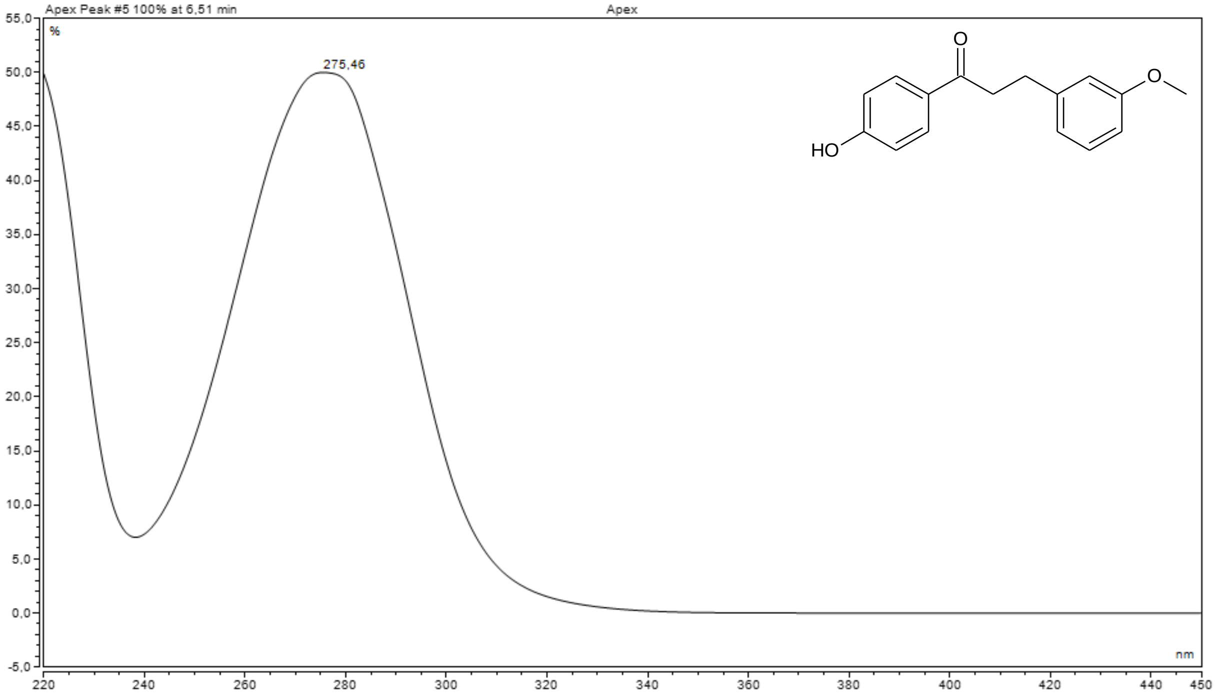

Figure S25. UV spectrum of 4′-hydroxy-3-methoxydihydrochalcone (**3a**)

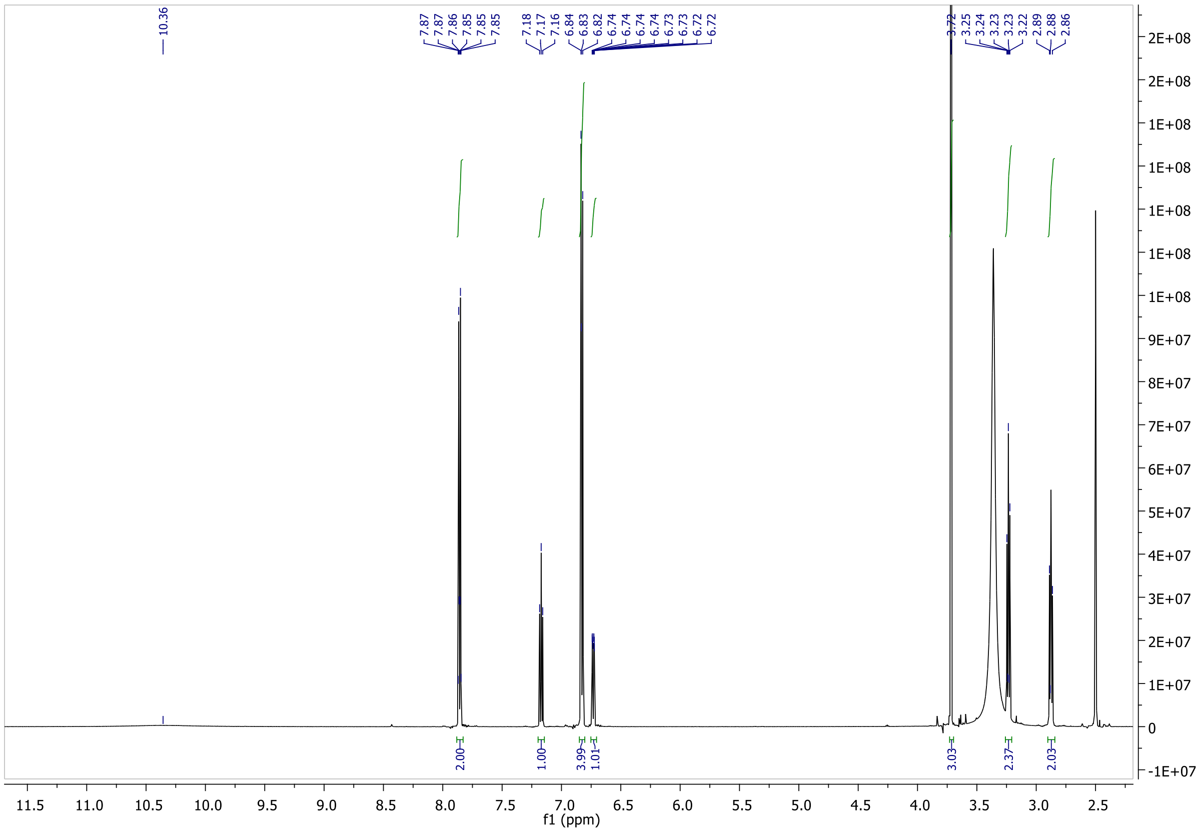

Figure S26. ^{1}H NMR spectrum of 4′-hydroxy-3-methoxydihydrochalcone (**3a**)

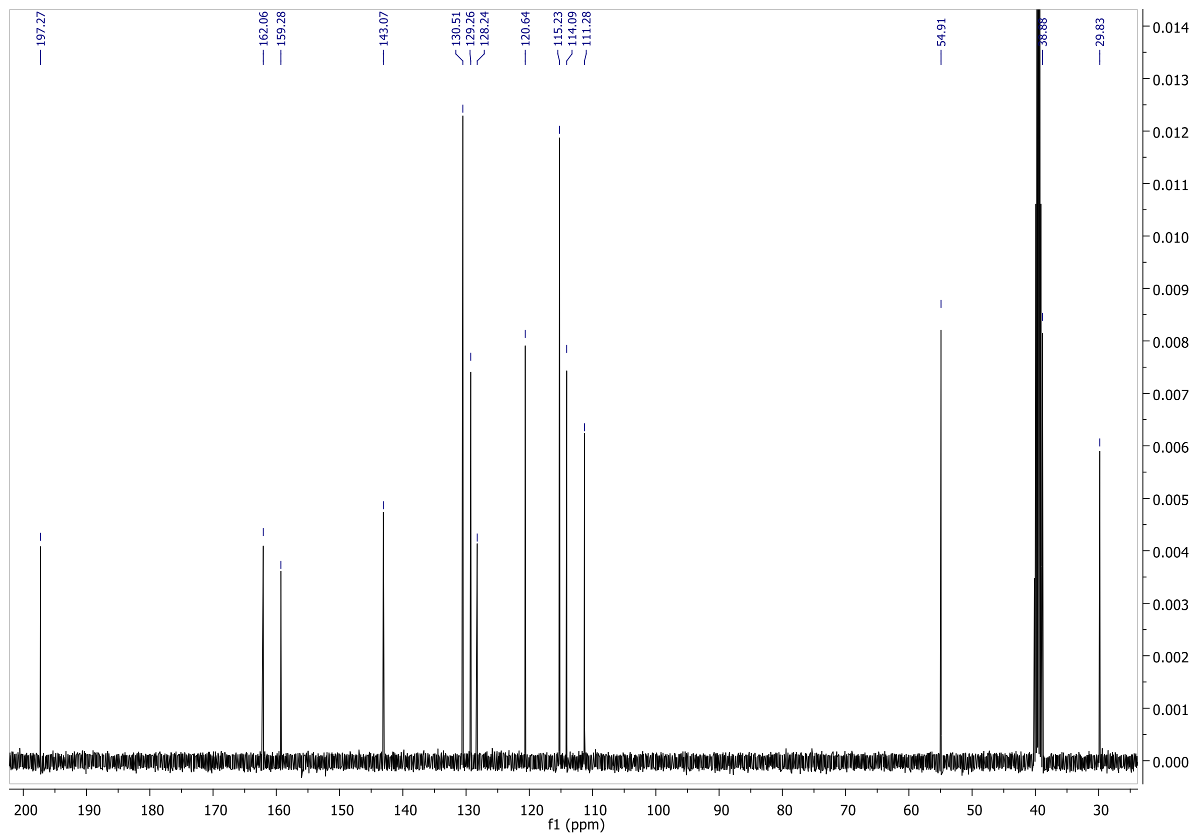

Figure S27. ^{13}C NMR spectrum of 4′-hydroxy-3-methoxydihydrochalcone (**3a**)

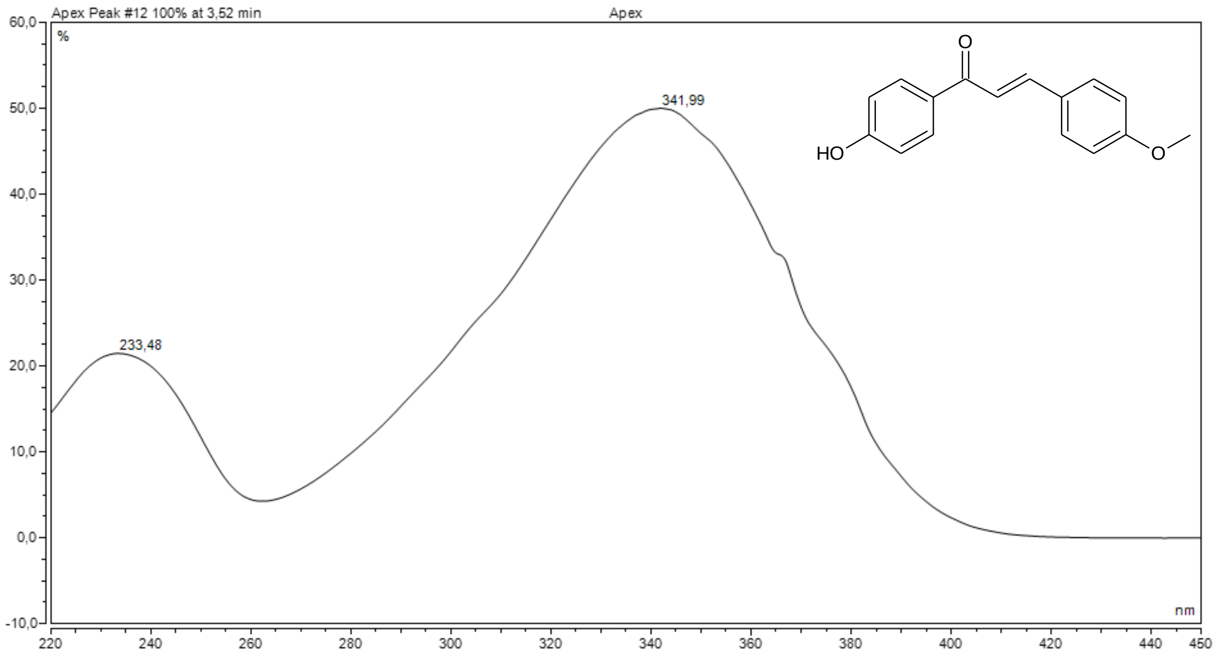

Figure S28. UV spectrum of *trans*-4′-hydroxy-4-methoxychalcone (*trans*-**4**)

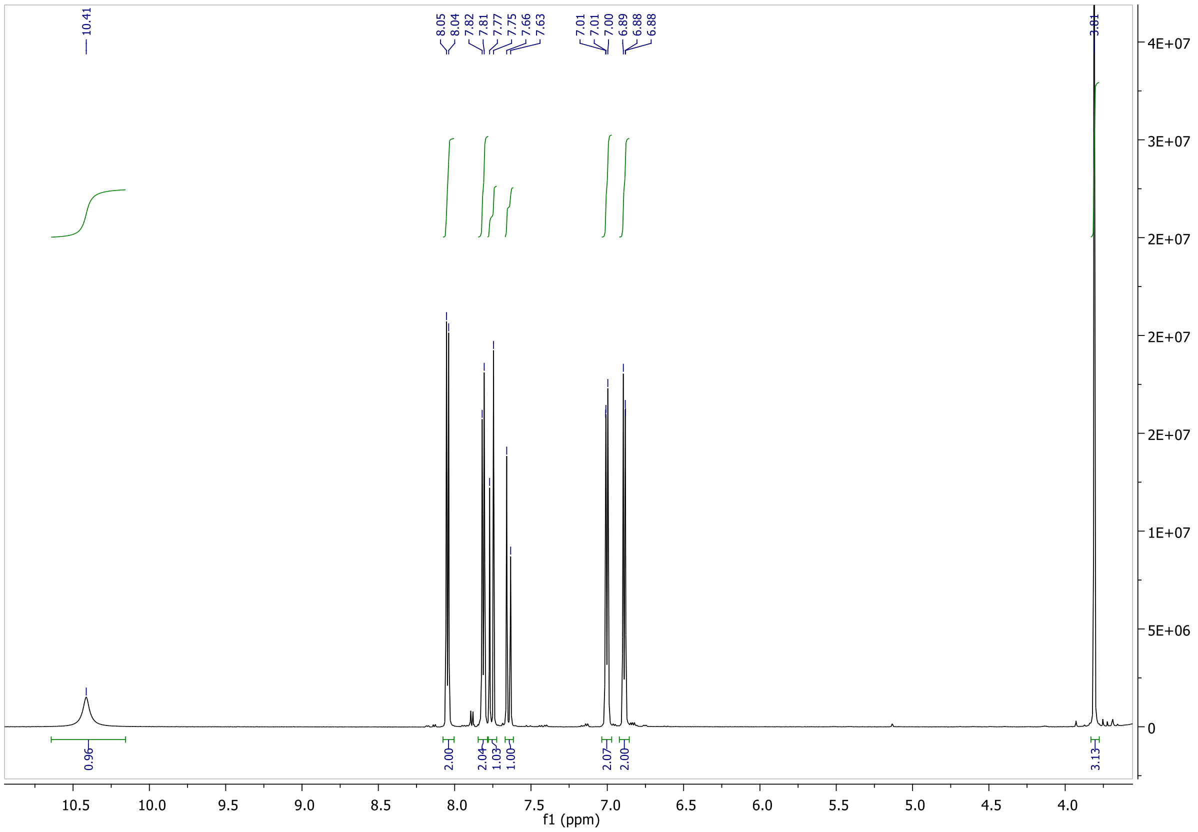

Figure S29. ^{1}H NMR spectrum of *trans*-4′-hydroxy-4-methoxychalcone (*trans*-**4**)

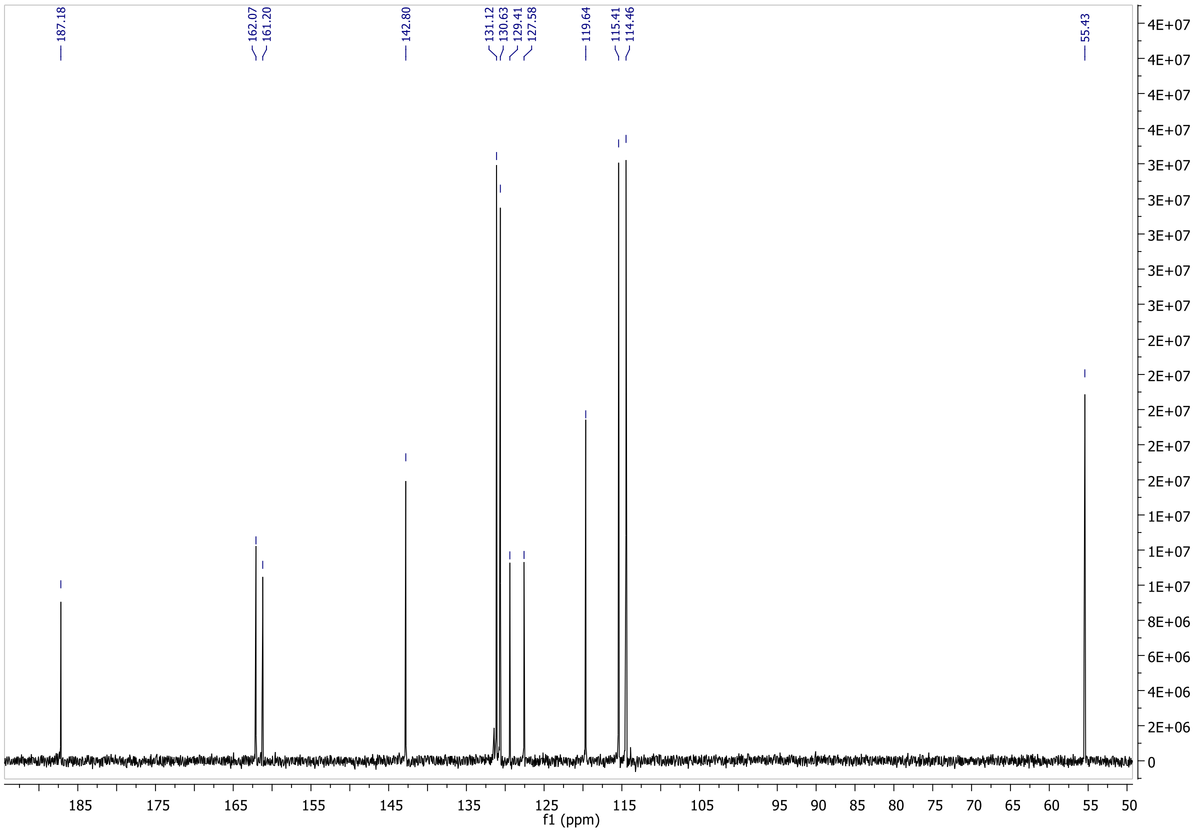

Figure S30. ^{13}C NMR spectrum of *trans*-4′-hydroxy-4-methoxychalcone (*trans*-**4**)

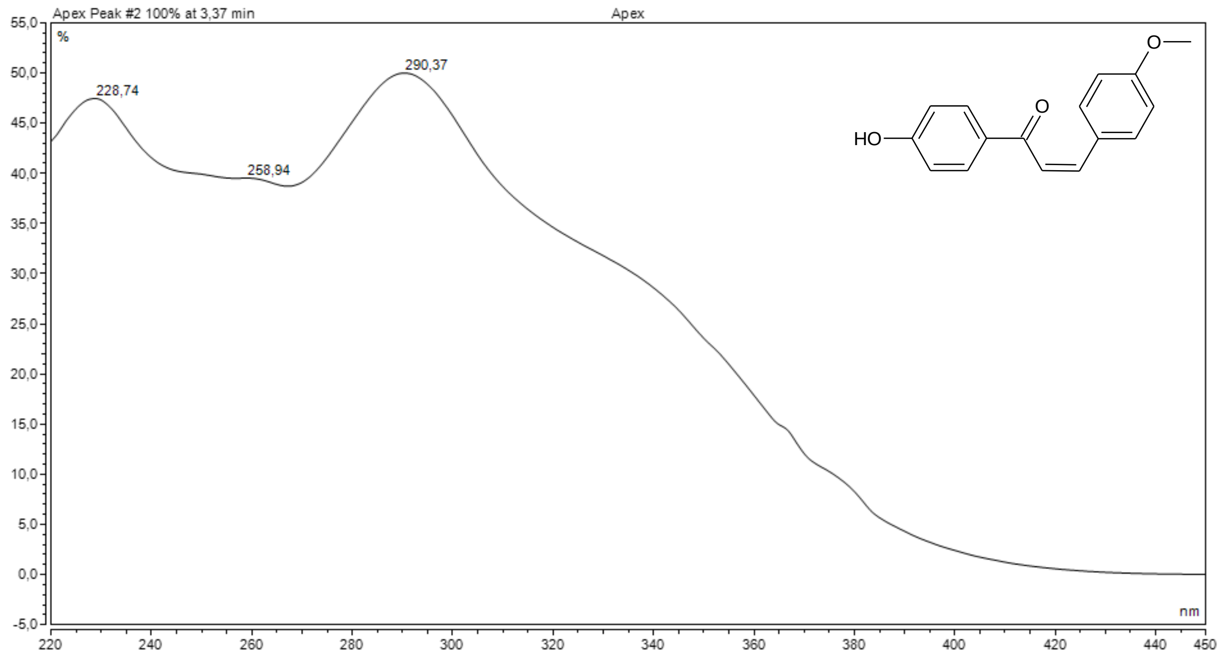

Figure S31. UV spectrum of *cis*-4′-hydroxy-4-methoxychalcone (*cis*-**4**)

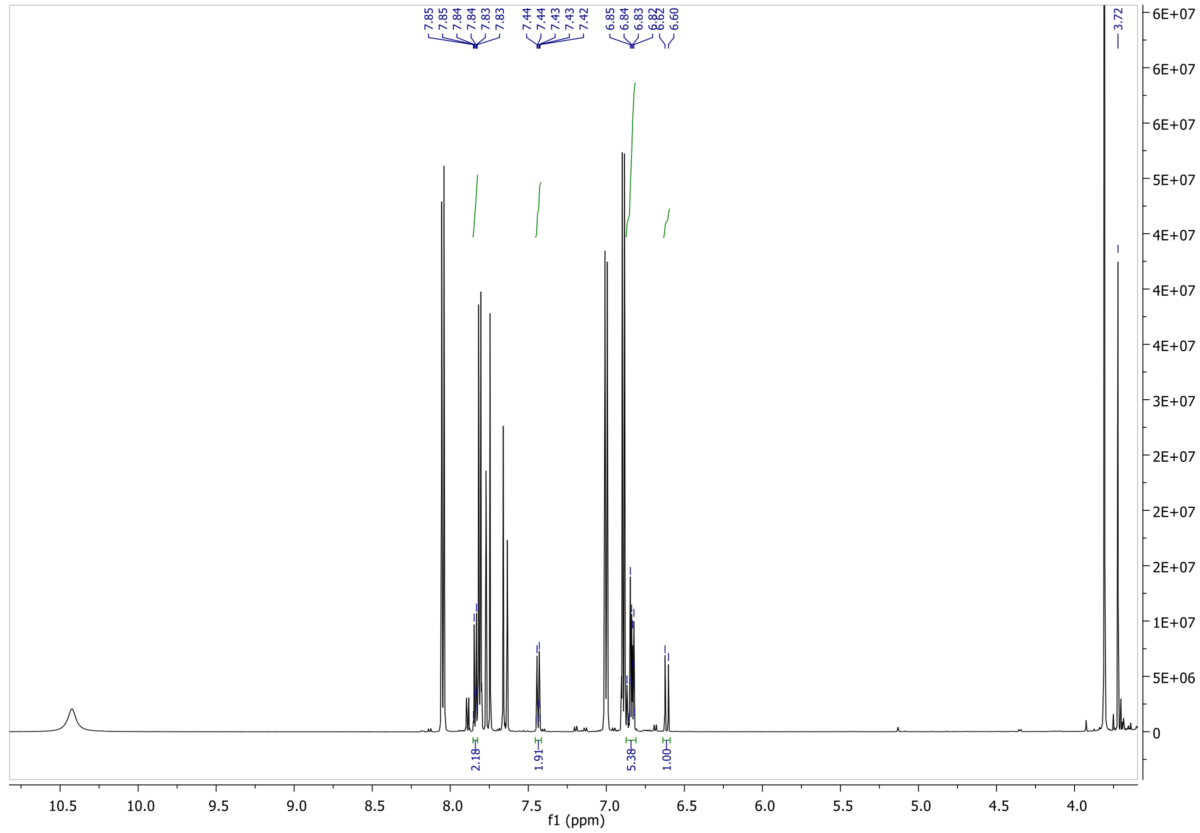

Figure S32. ^{1}H NMR spectrum of *cis*-4′-hydroxy-4-methoxychalcone (*cis*-**4**)

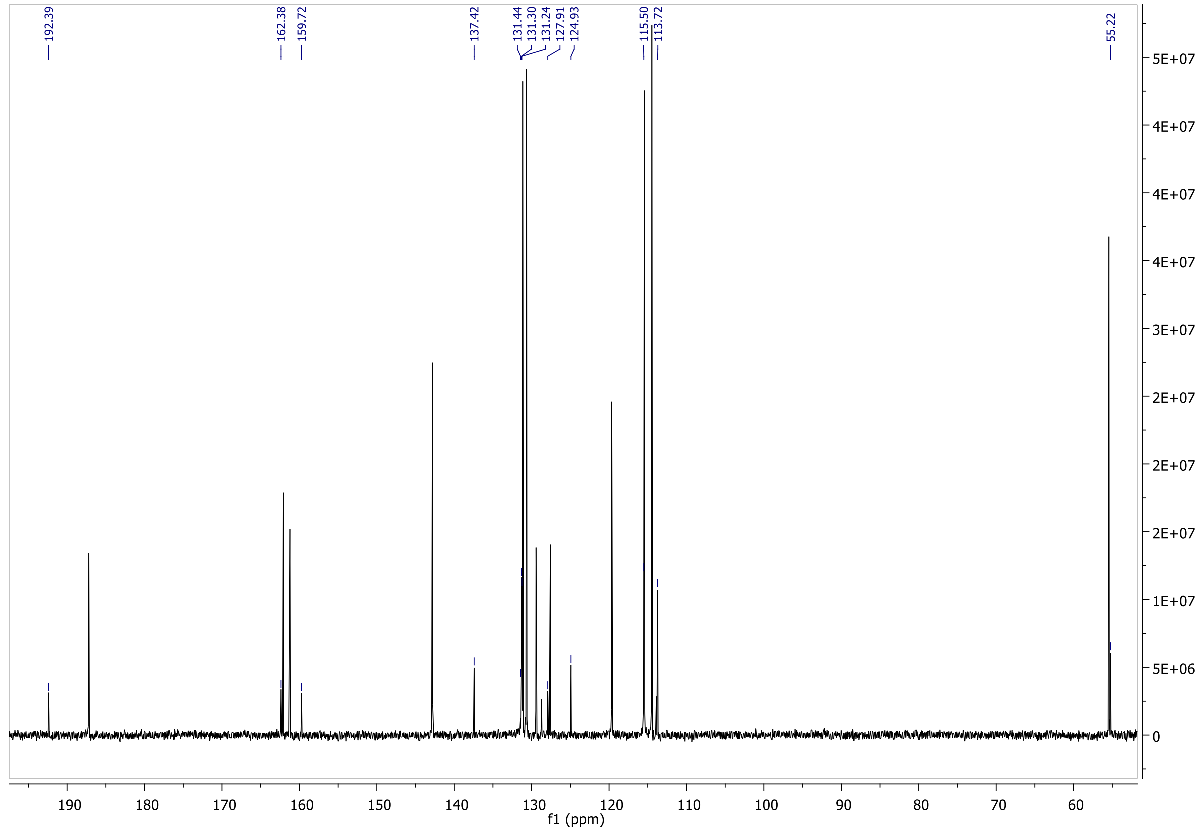

Figure S33. ^{13}C NMR spectrum of *cis*-4′-hydroxy-4-methoxychalcone (*cis*-**4**)

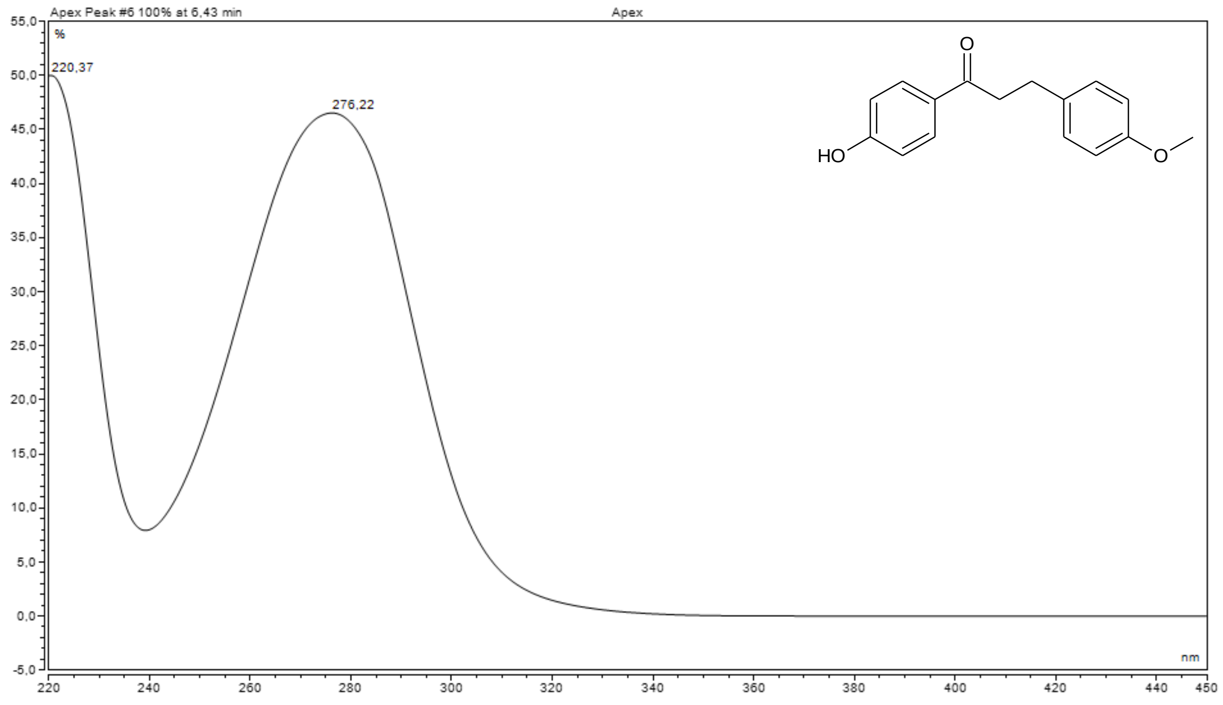

Figure S34. UV spectrum of 4′-hydroxy-4-methoxydihydrochalcone (**4a**)

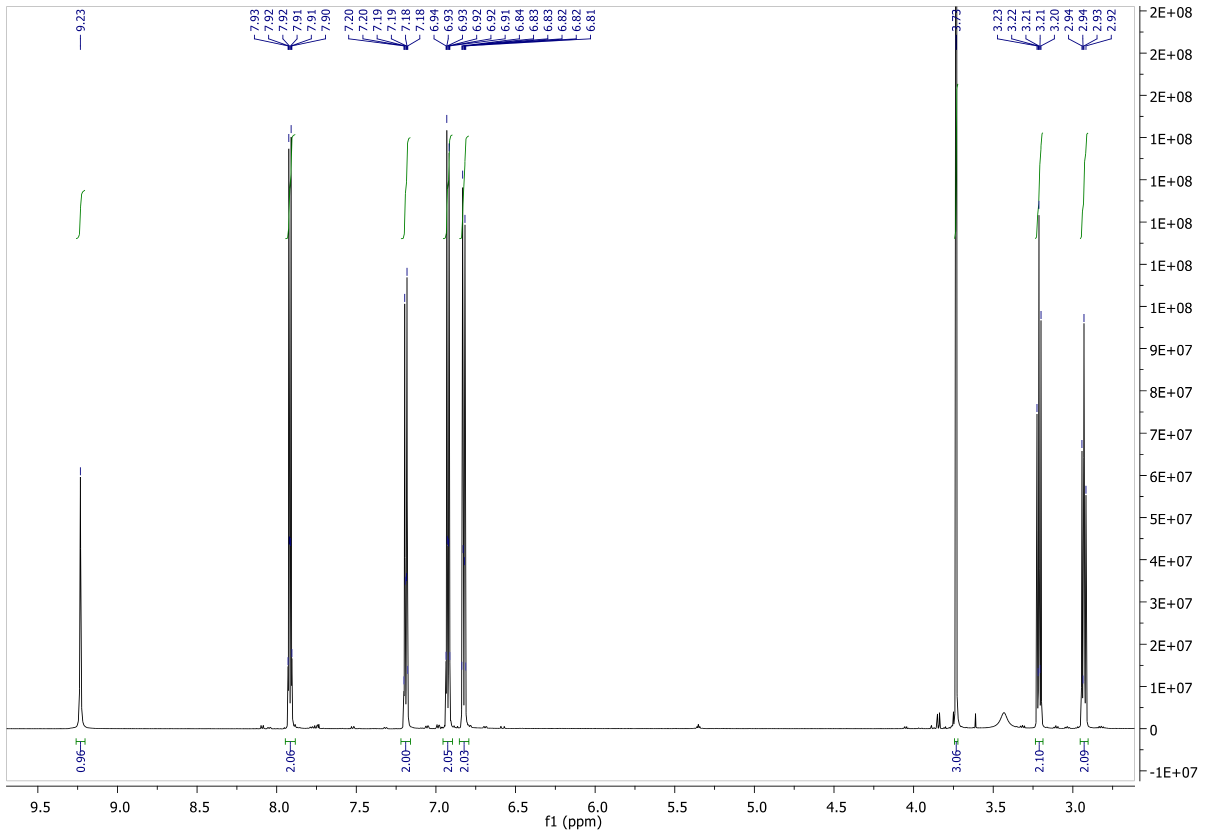

Figure S35. ^{1}H NMR spectrum of 4′-hydroxy-4-methoxydihydrochalcone (**4a**)

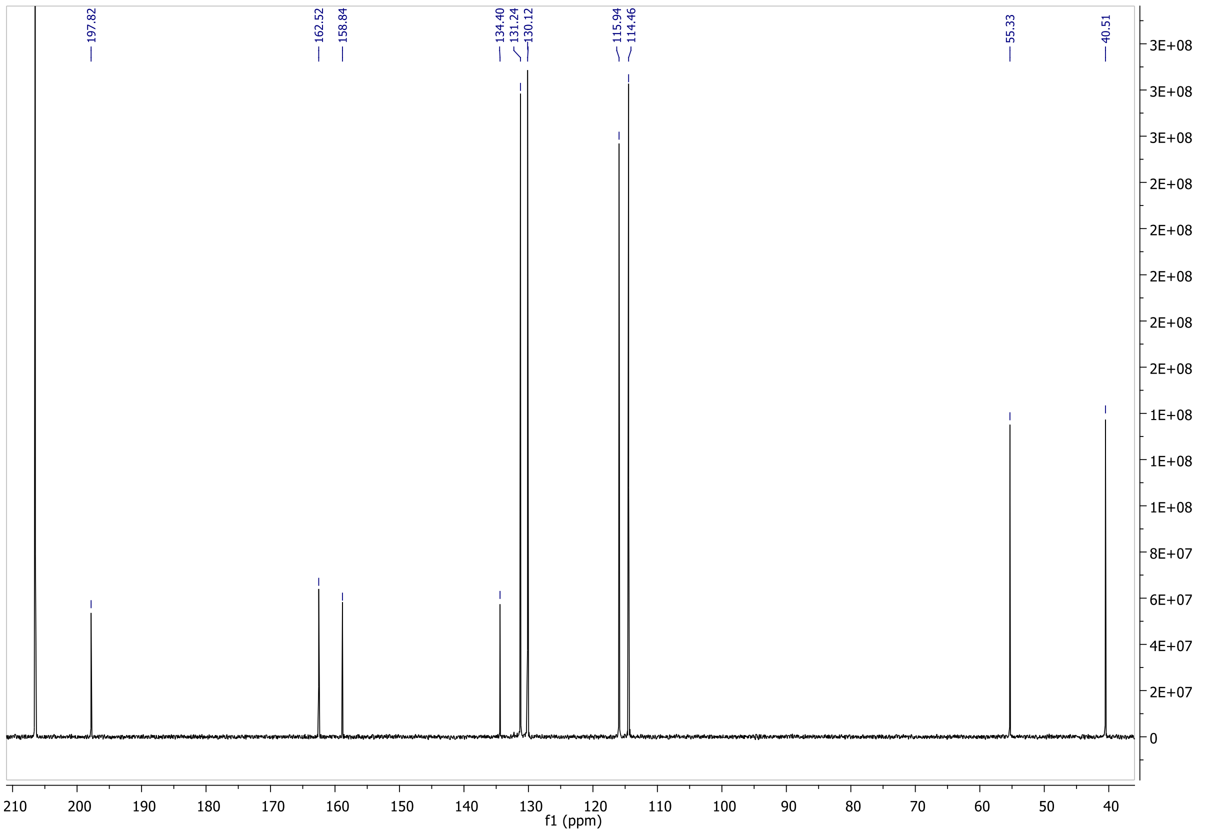

Figure S36. ^{13}C NMR spectrum of 4′-hydroxy-4-methoxydihydrochalcone (**4a**)

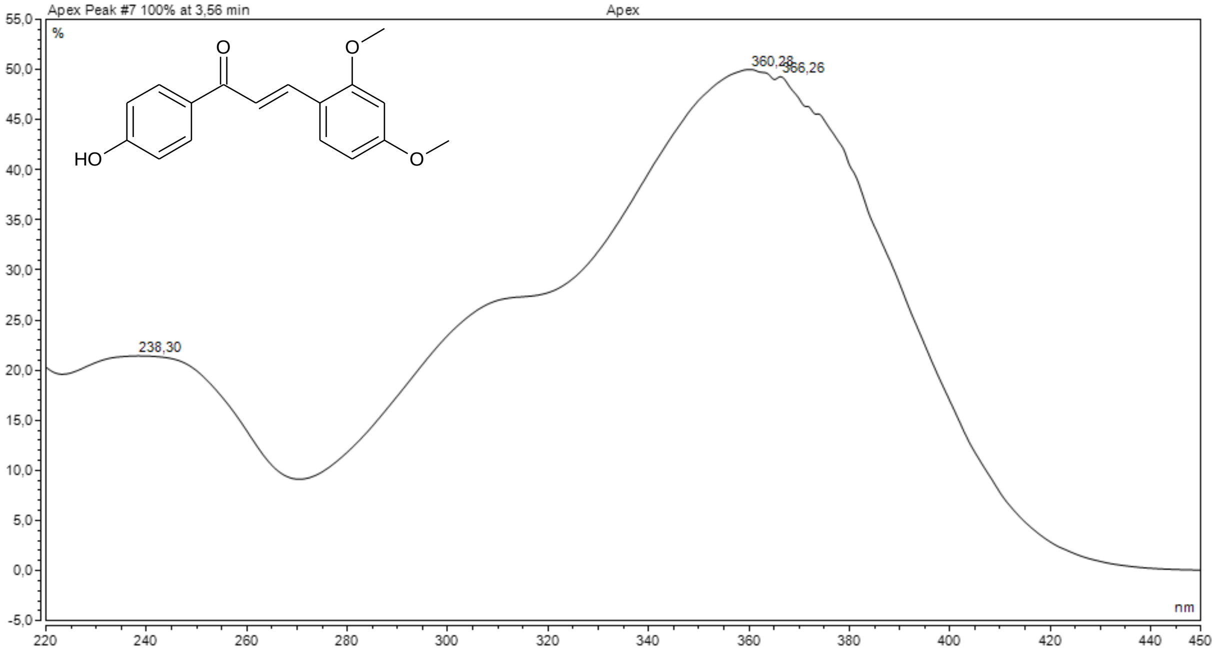

Figure S37. UV spectrum of *trans*-4′-hydroxy-2,4-dimethoxychalcone (*trans*-**5**)

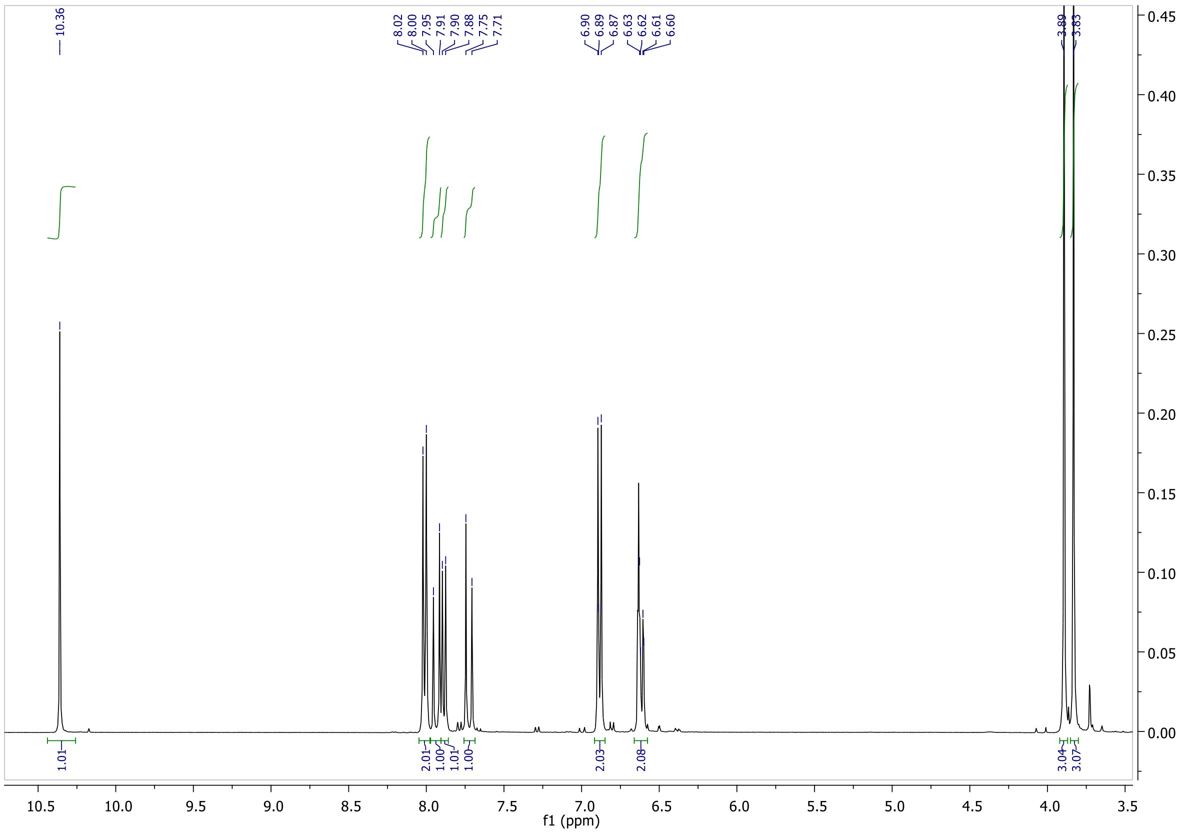

Figure S38. ^{1}H NMR spectrum of *trans*-4′-hydroxy-2,4-dimethoxychalcone (*trans*-**5**)

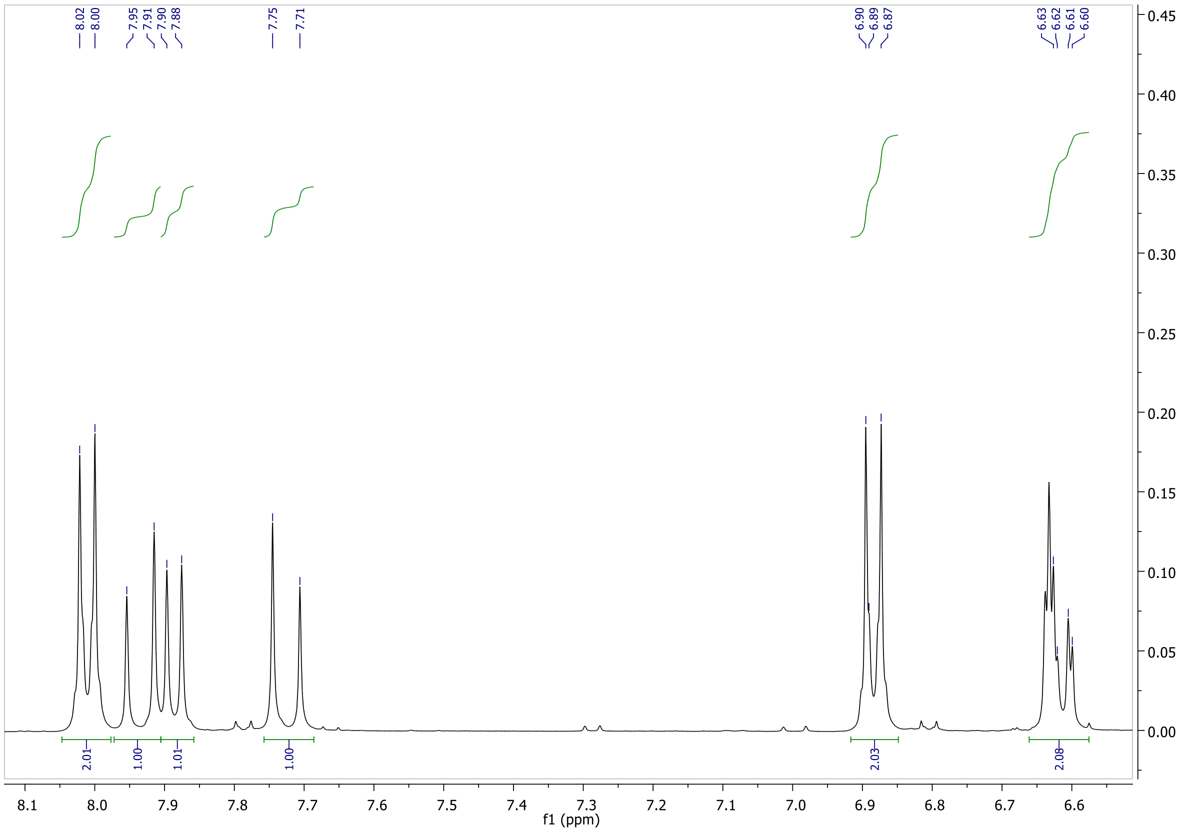

Figure S39. Part of the ¹H NMR spectrum of *trans*-4′-hydroxy-2,4-dimethoxychalcone (*trans*-**5**)

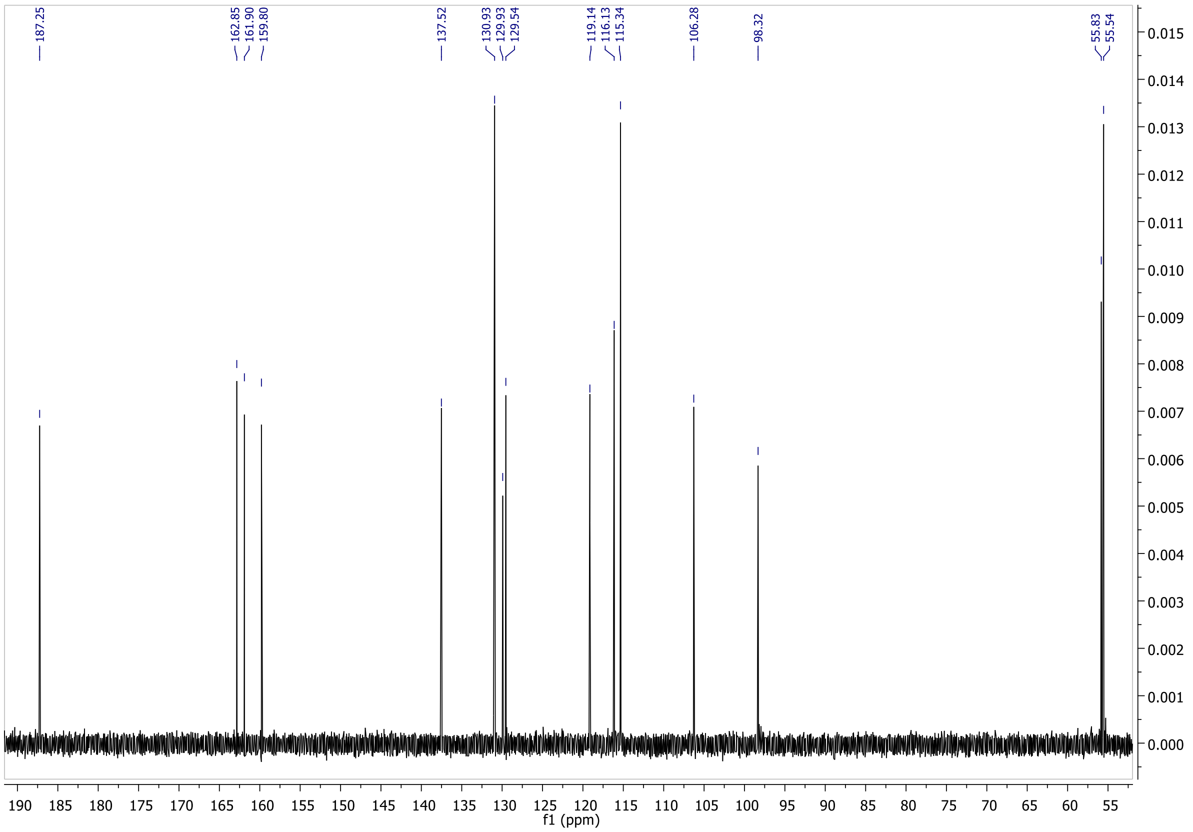

Figure S40. ¹³C NMR spectrum of *trans*-4′-hydroxy-2,4-dimethoxychalcone (*trans*-**5**)

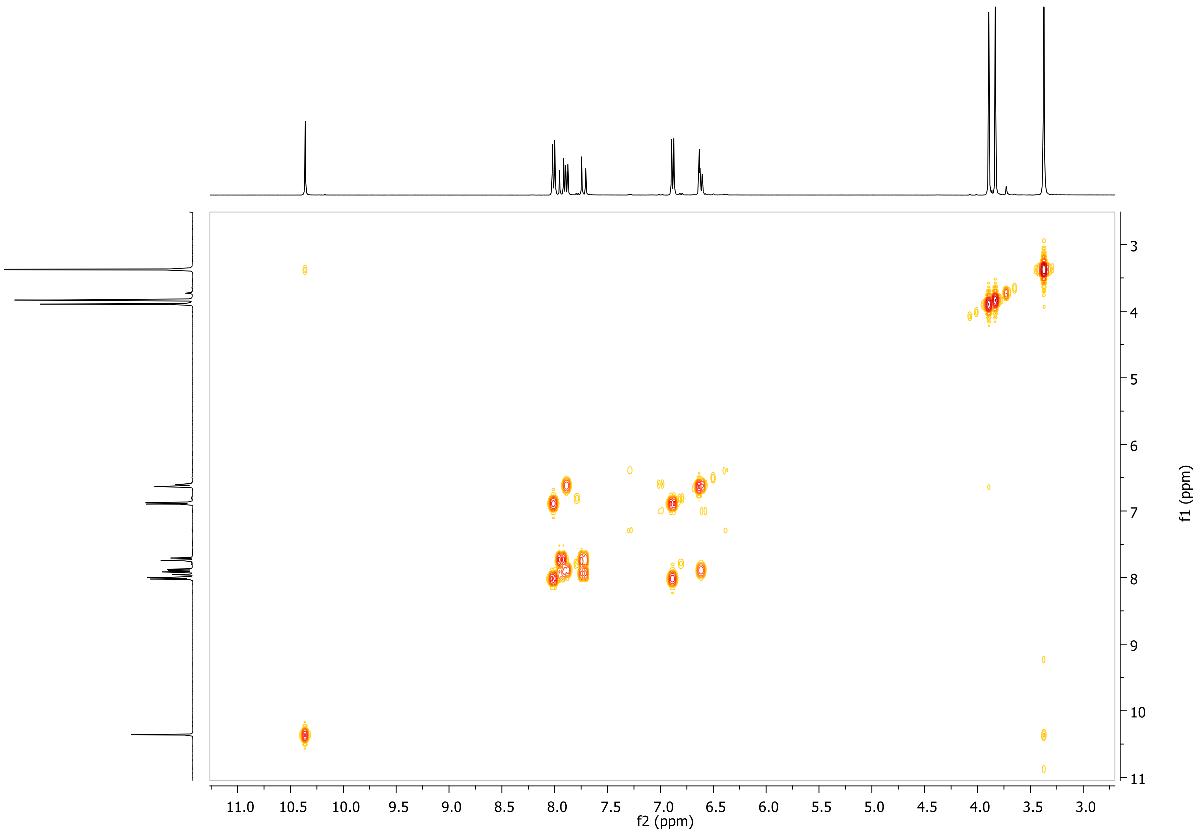

Figure S41. COSY NMR spectrum of *trans*-4′-hydroxy-2,4-dimethoxychalcone (*trans*-**5**)

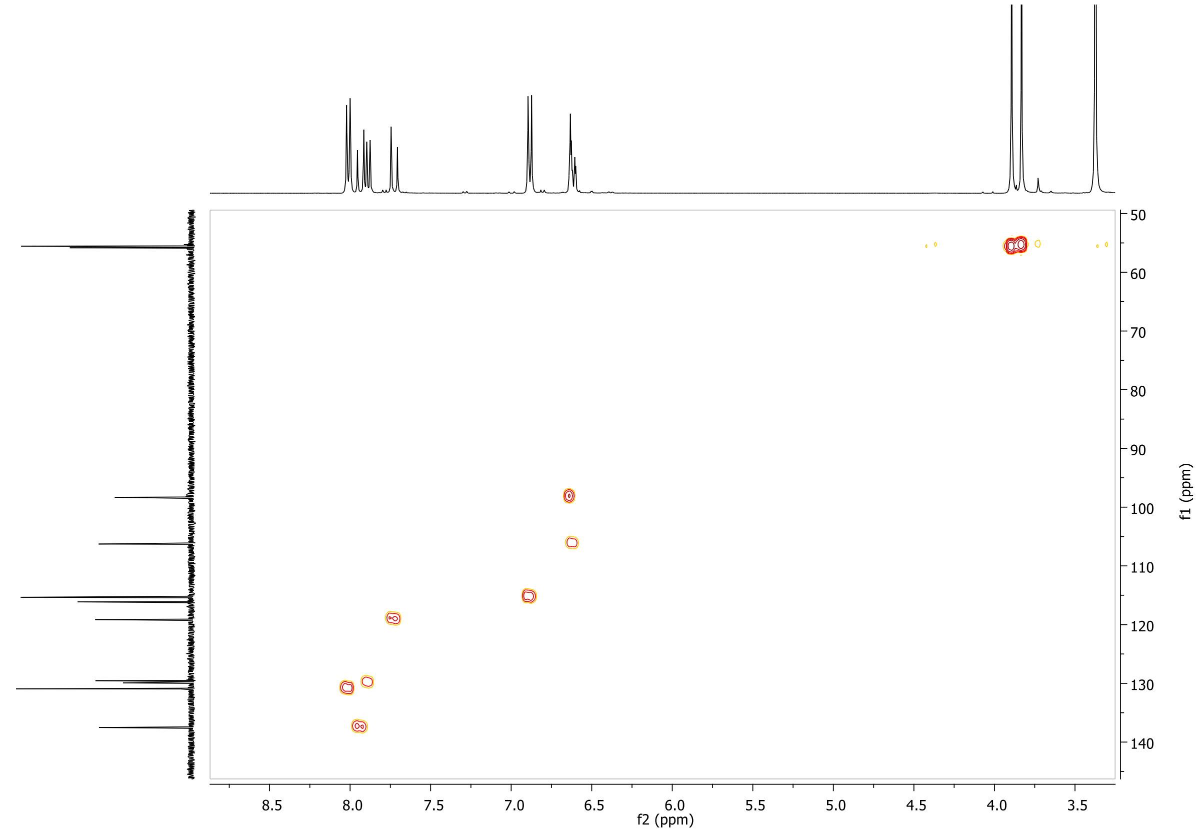

Figure S42. HSQC NMR spectrum of *trans*-4′-hydroxy-2,4-dimethoxychalcone (*trans*-**5**)

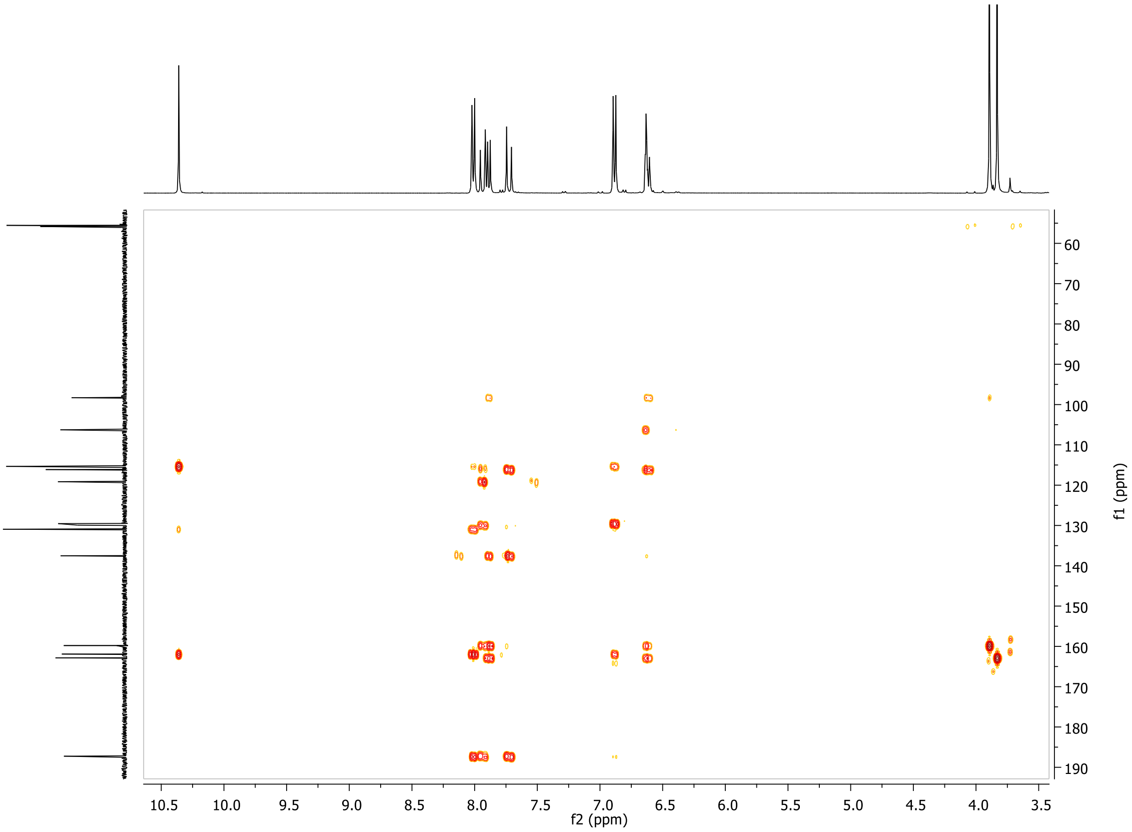

Figure S43. HMBC NMR spectrum of *trans*-4′-hydroxy-2,4-dimethoxychalcone (*trans*-**5**)

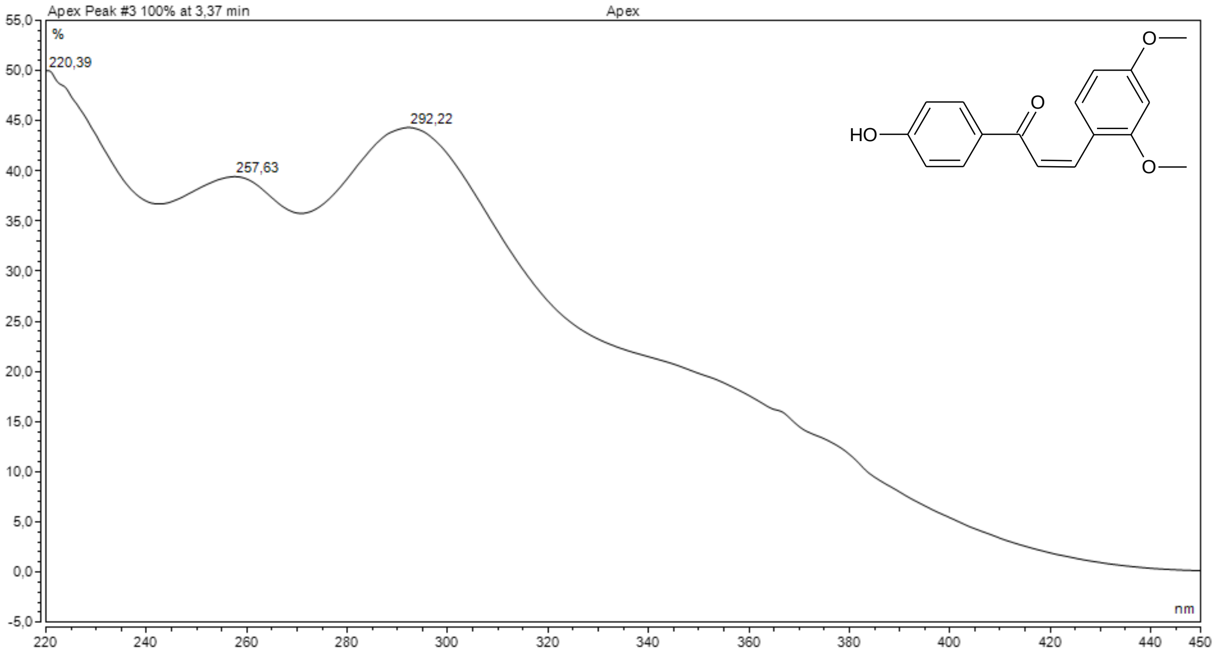

Figure S44. UV spectrum of *cis*-4′-hydroxy-2,4-dimethoxychalcone (*cis*-**5**)

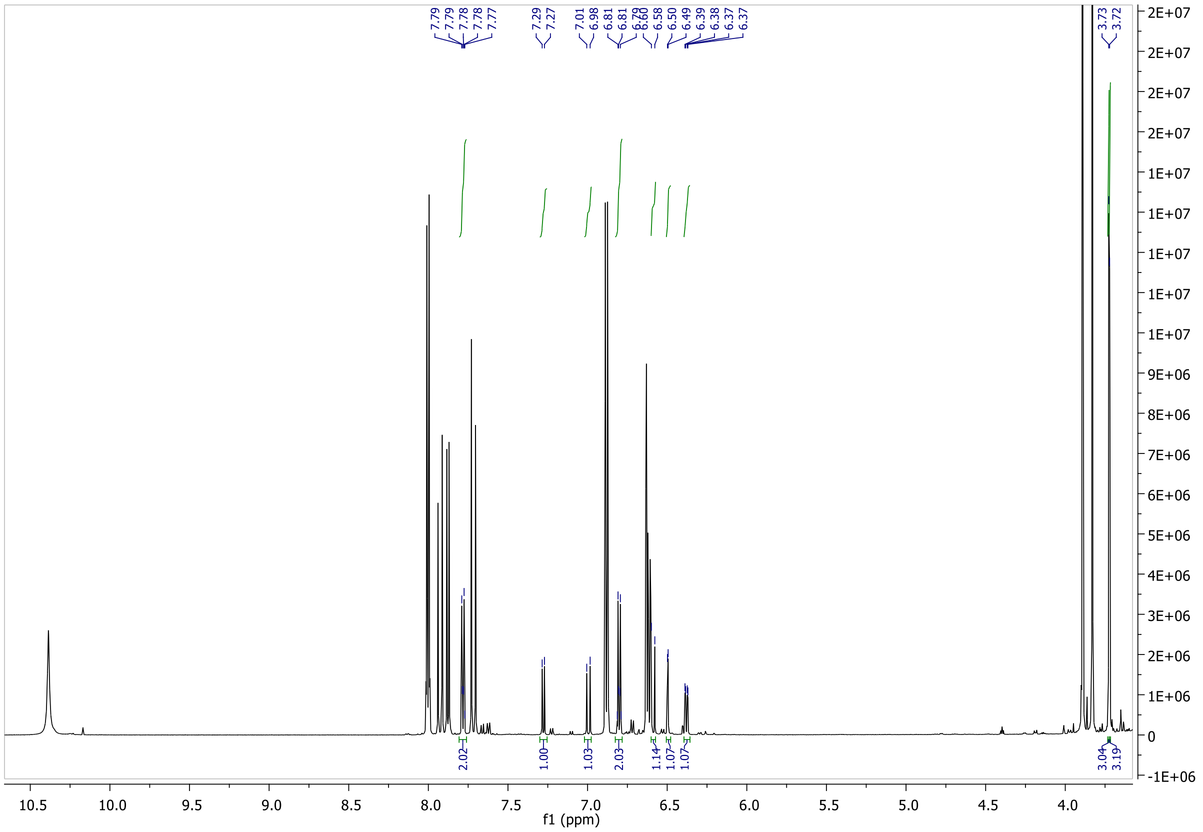

Figure S45. ^{1}H NMR spectrum of *cis*-4′-hydroxy-2,4-dimethoxychalcone (*cis*-**5**)

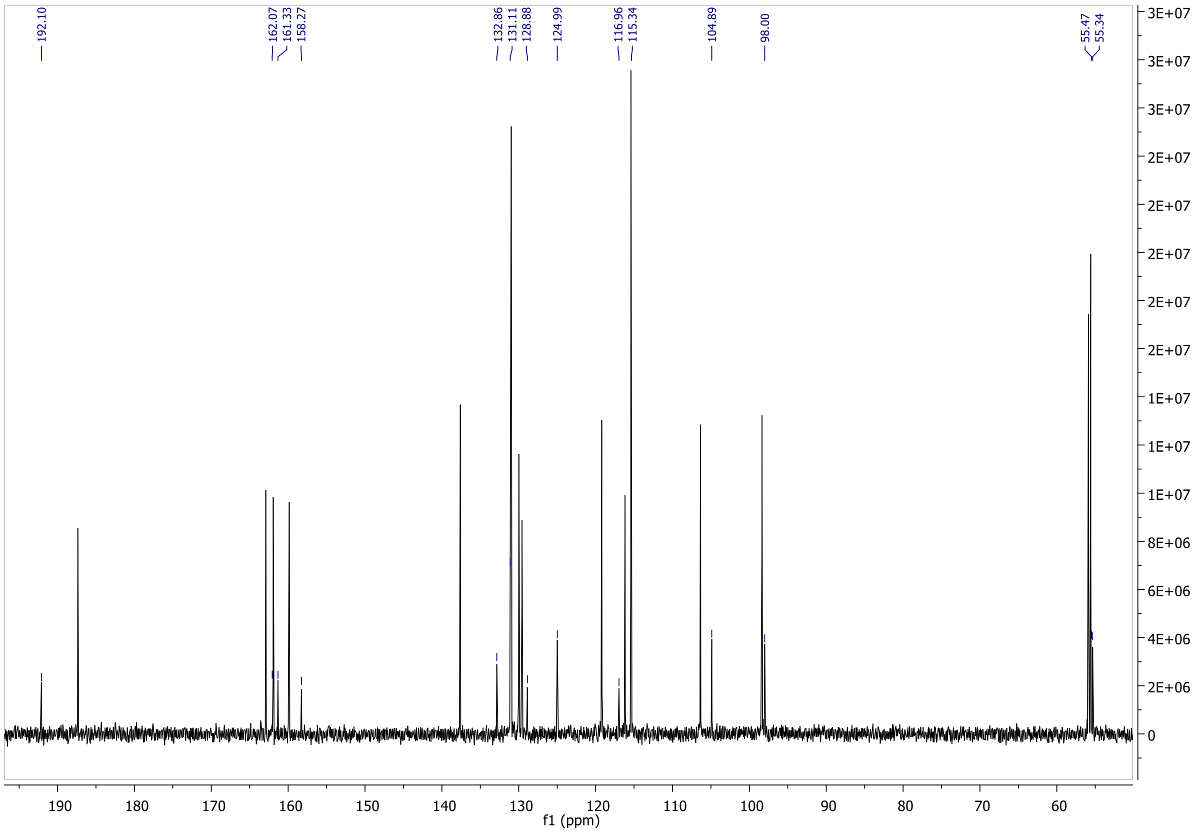

Figure S46. ^{13}C NMR spectrum of *cis*-4′-hydroxy-2,4-dimethoxychalcone (*cis*-**5**)

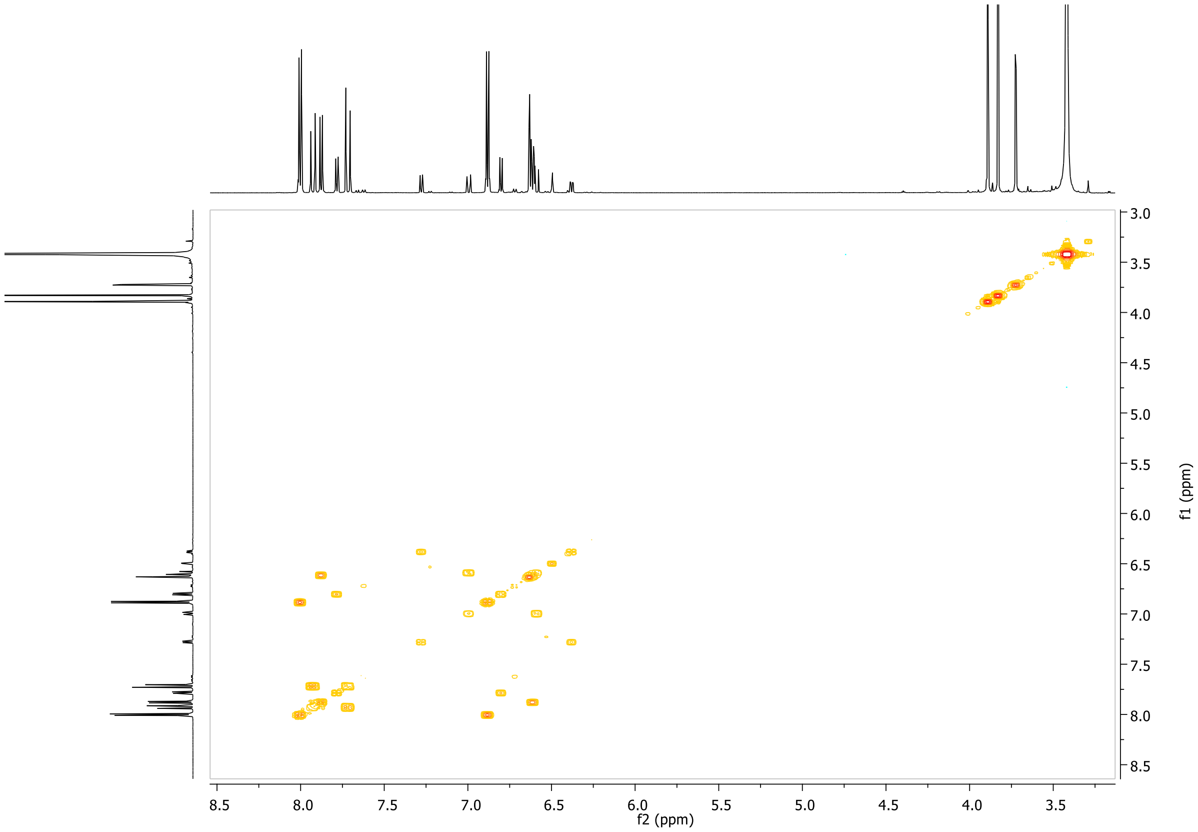

Figure S47. COSY spectrum of *cis*-4′-hydroxy-2,4-dimethoxychalcone (*cis*-**5**)

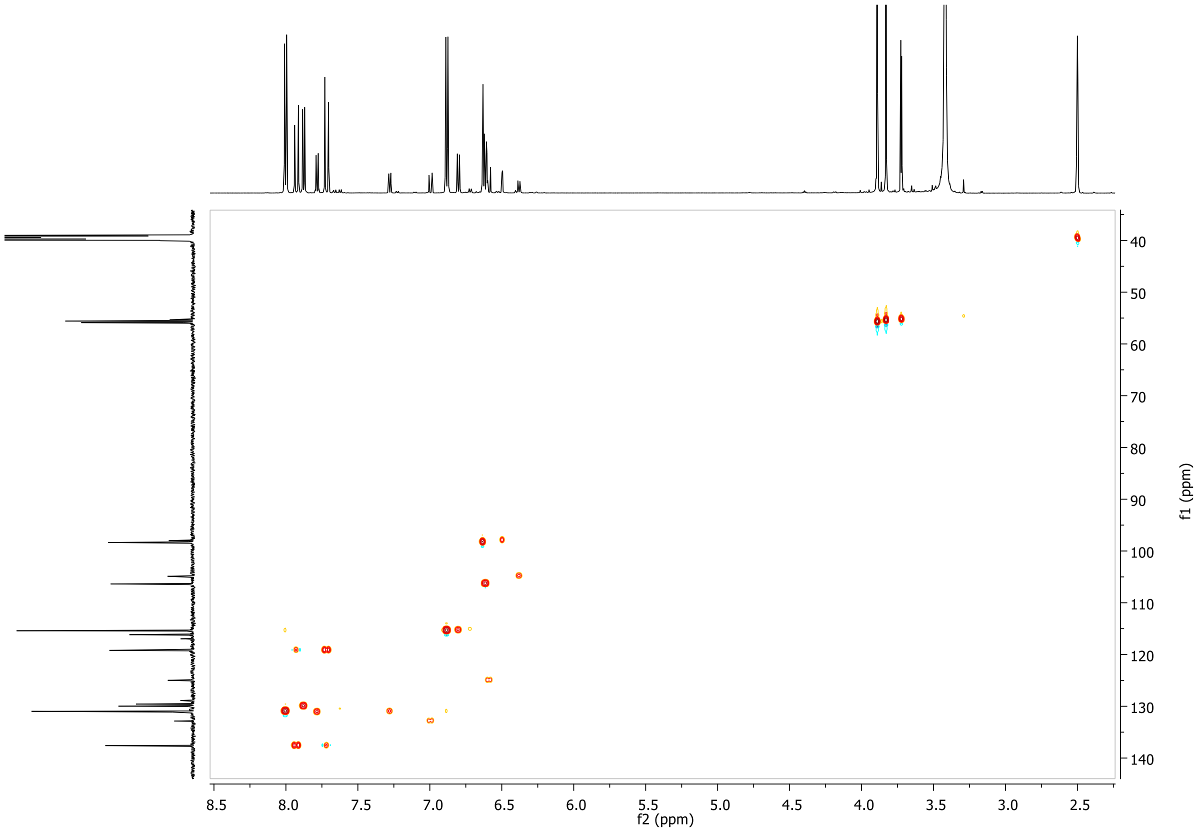

Figure S48. HSQC NMR spectrum of *cis*-4′-hydroxy-2,4-dimethoxychalcone (*cis*-**5**)

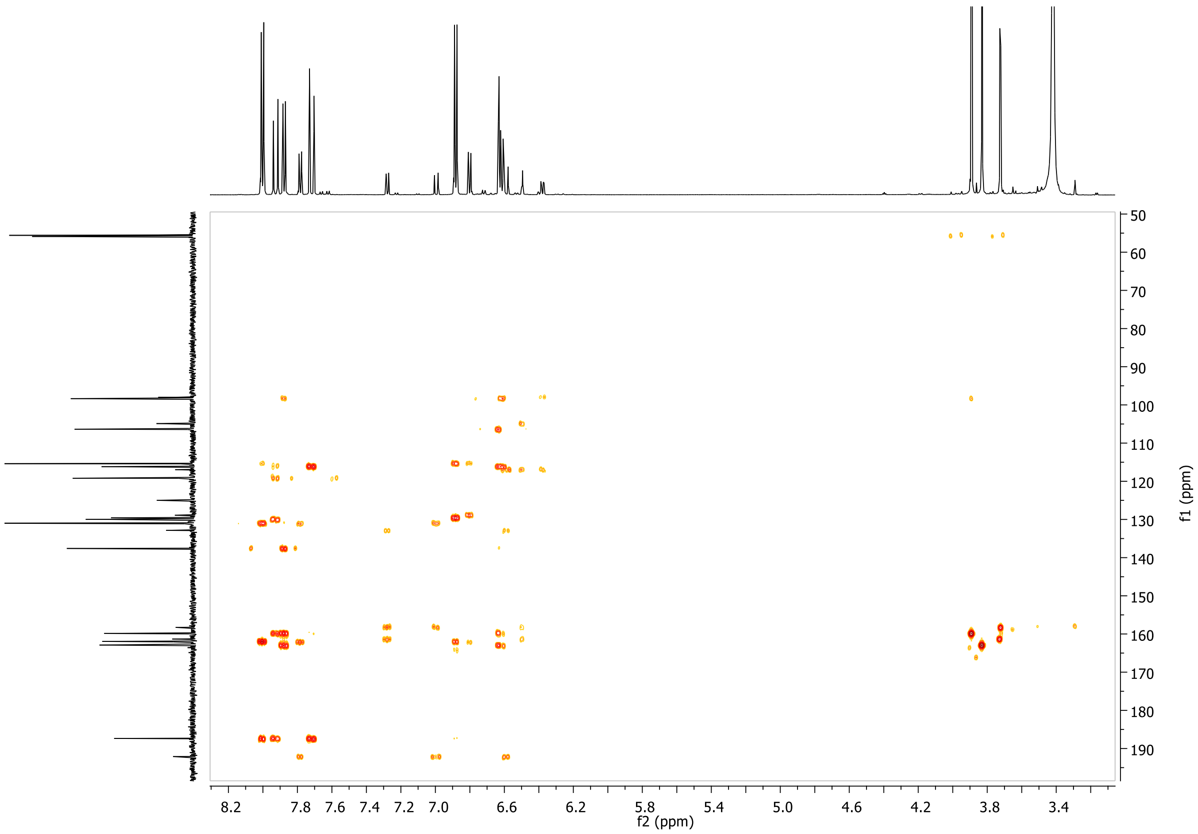

Figure S49. HMBC NMR spectrum of *cis*-4'-hydroxy-2,4-dimethoxychalcone (*cis*-**5**)

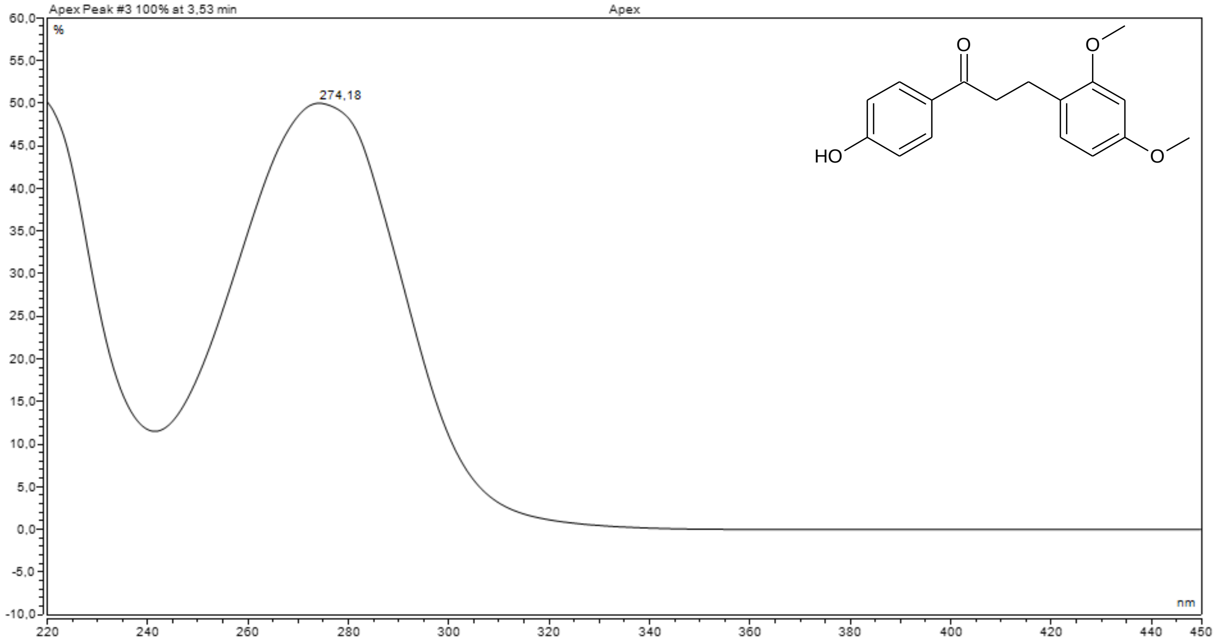

Figure S50. UV spectrum of 4′-hydroxy-2,4-dimetoxydihydrochalcone (**5a**)

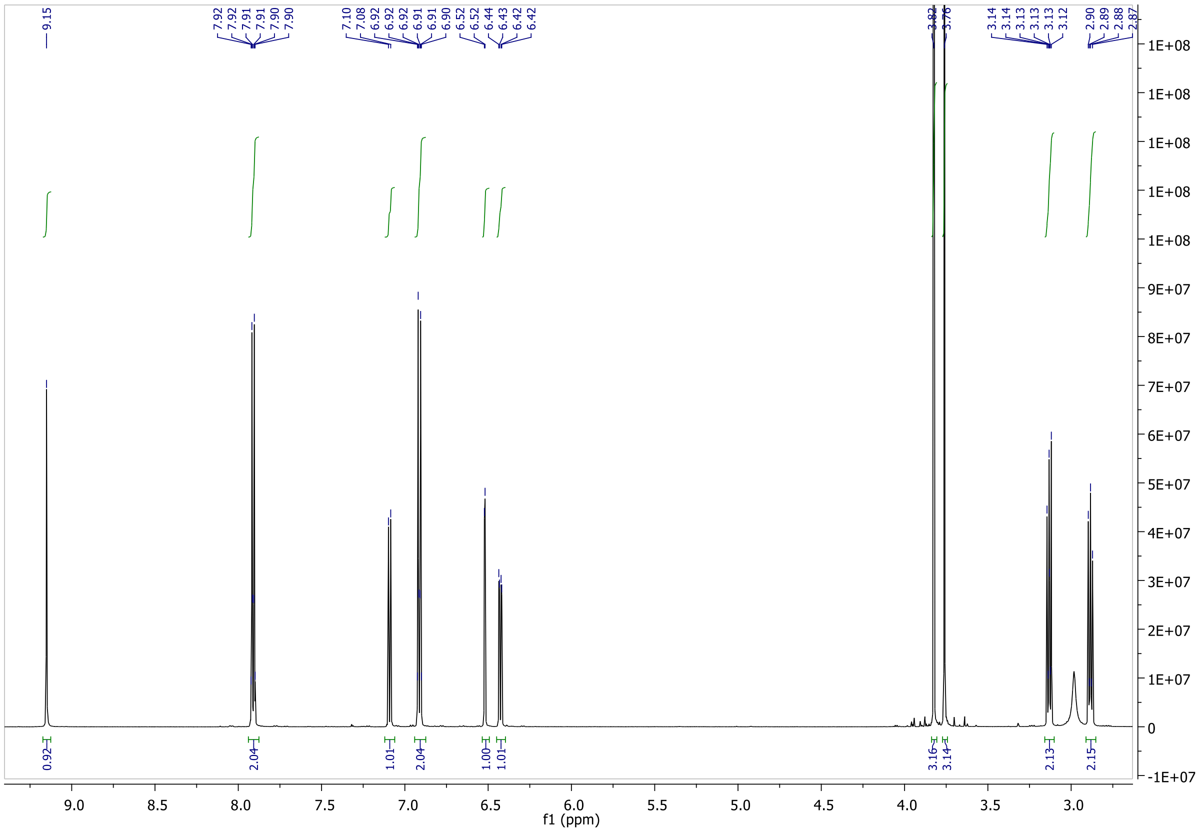

Figure S51. ^{1}H NMR spectrum of 4′-hydroxy-2,4-dimetoxydihydrochalcone (**5a**)

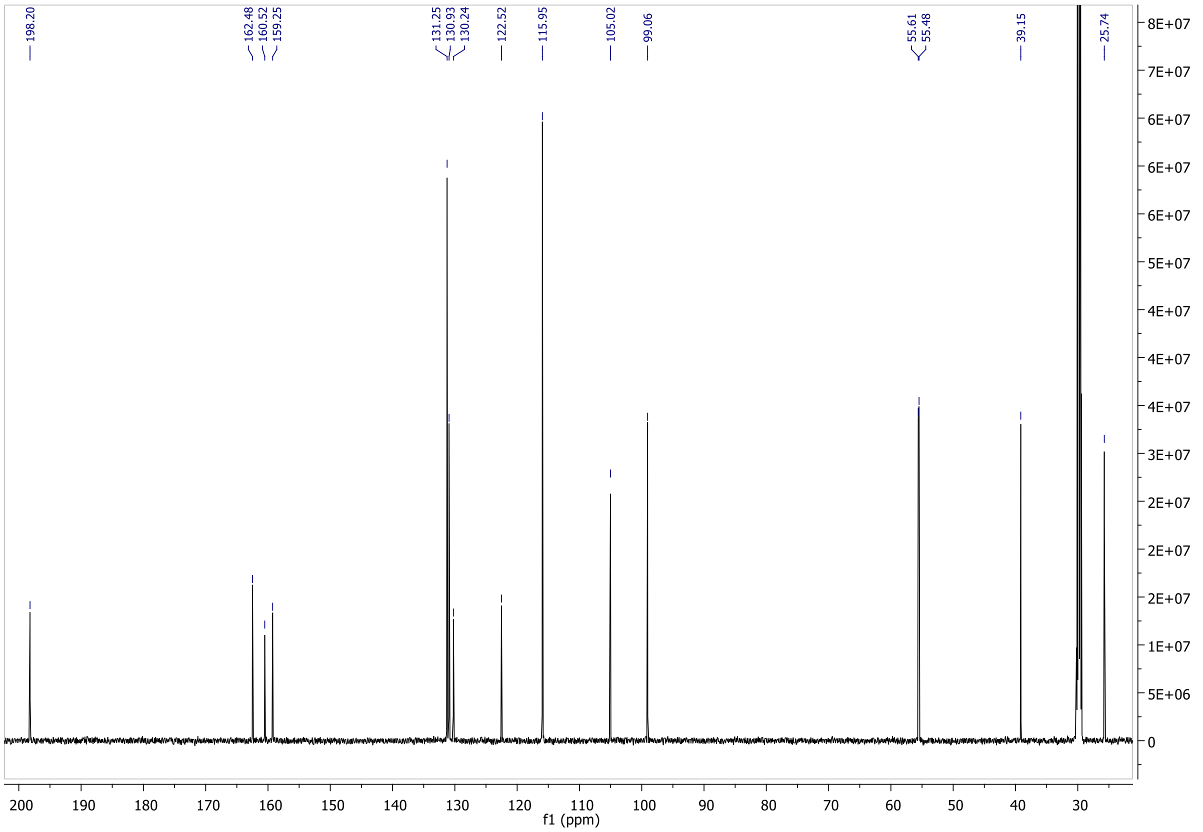

Figure S52. ^{13}C NMR spectrum of 4′-hydroxy-2,4-dimetoxydihydrochalcone (**5a**)

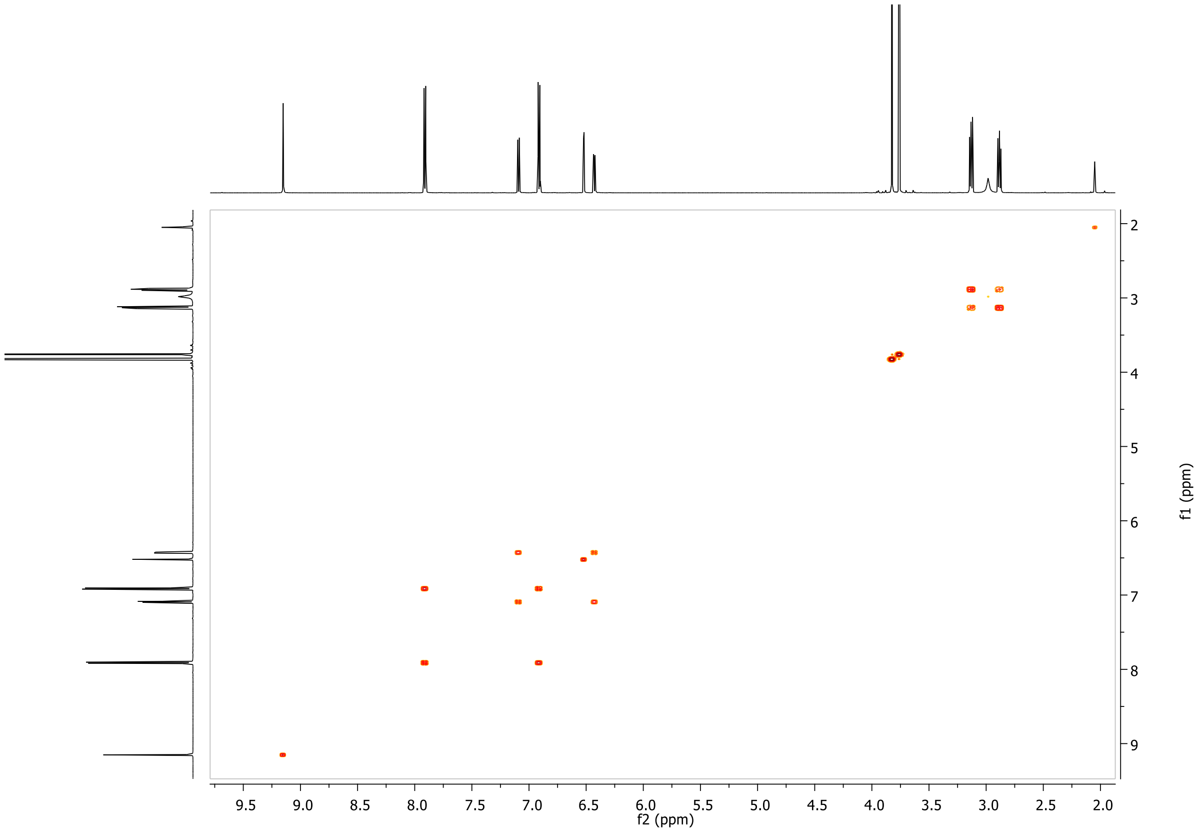

Figure S53. COSY NMR spectrum of 4′-hydroxy-2,4-dimetoxydihydrochalcone (**5a**)

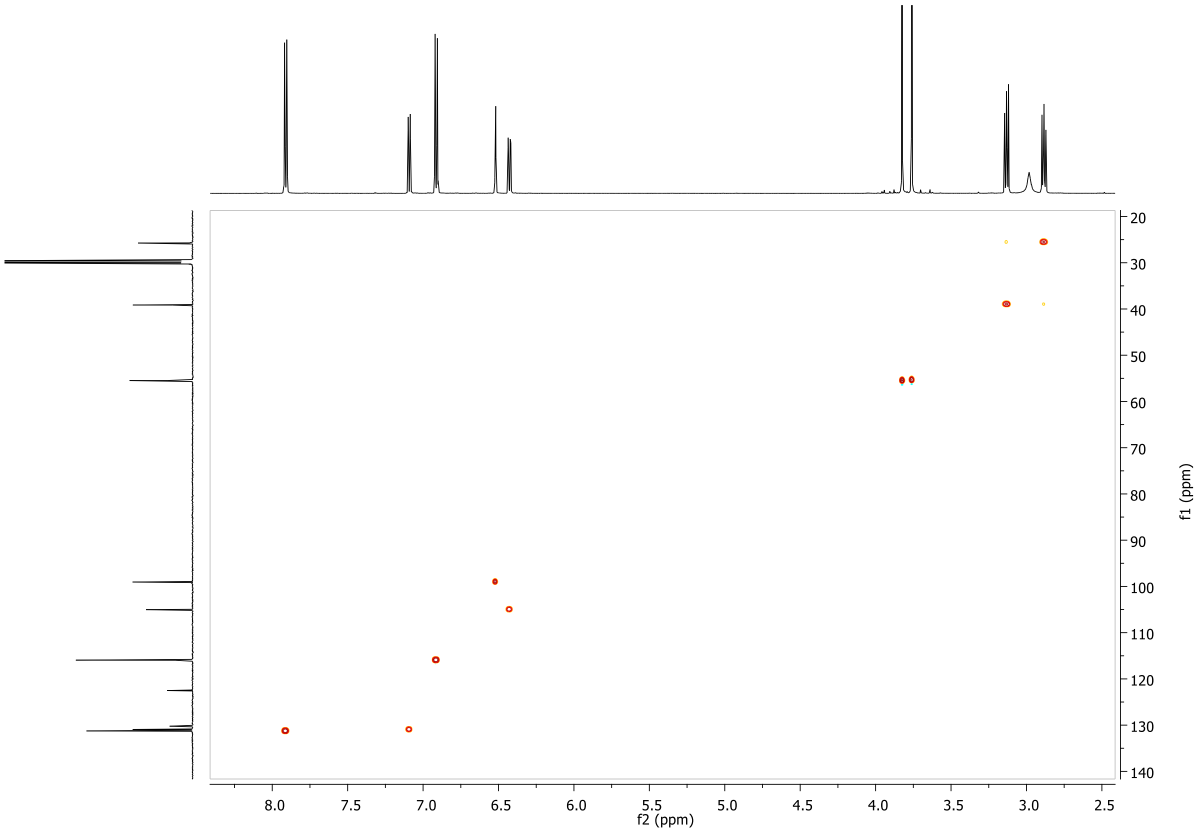

Figure S54. HSQC NMR spectrum of 4′-hydroxy-2,4-dimetoxydihydrochalcone (**5a**)

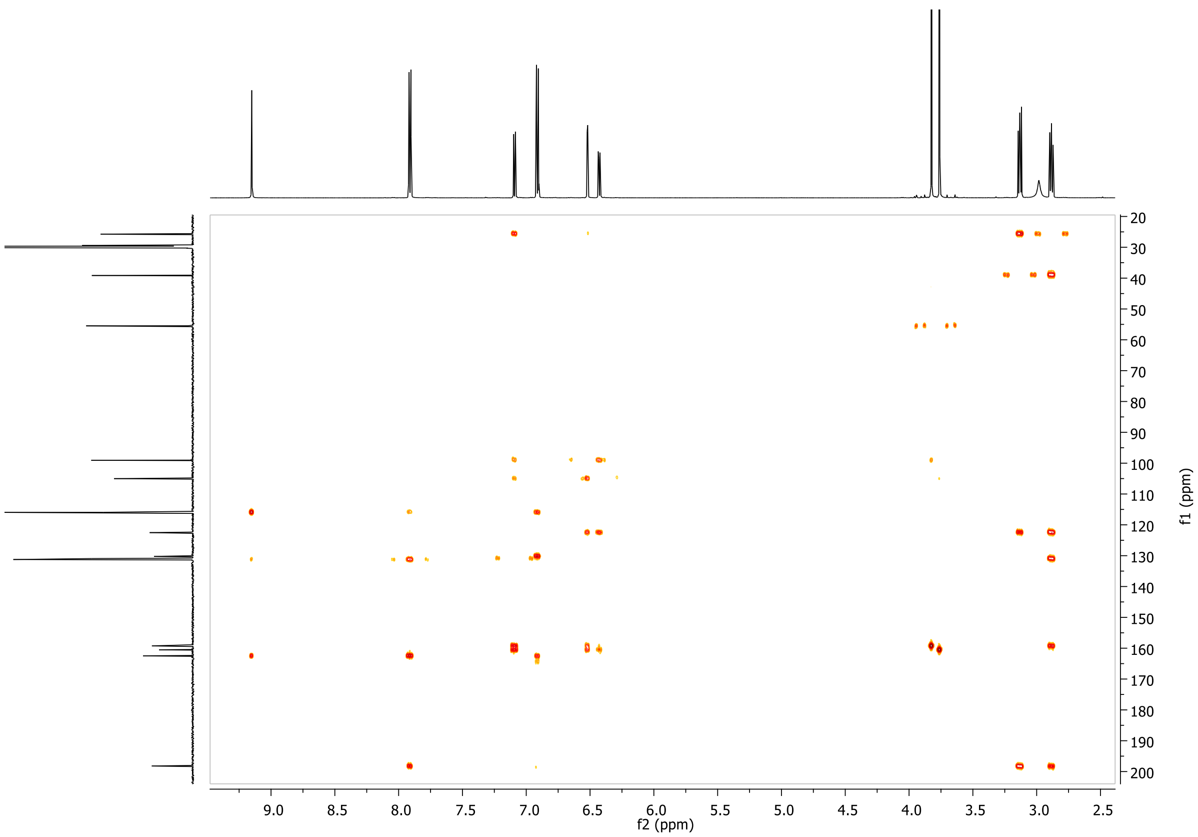

Figure S55. HMBC NMR spectrum of 4′-hydroxy-2,4-dimetoxydihydrochalcone (**5a**)

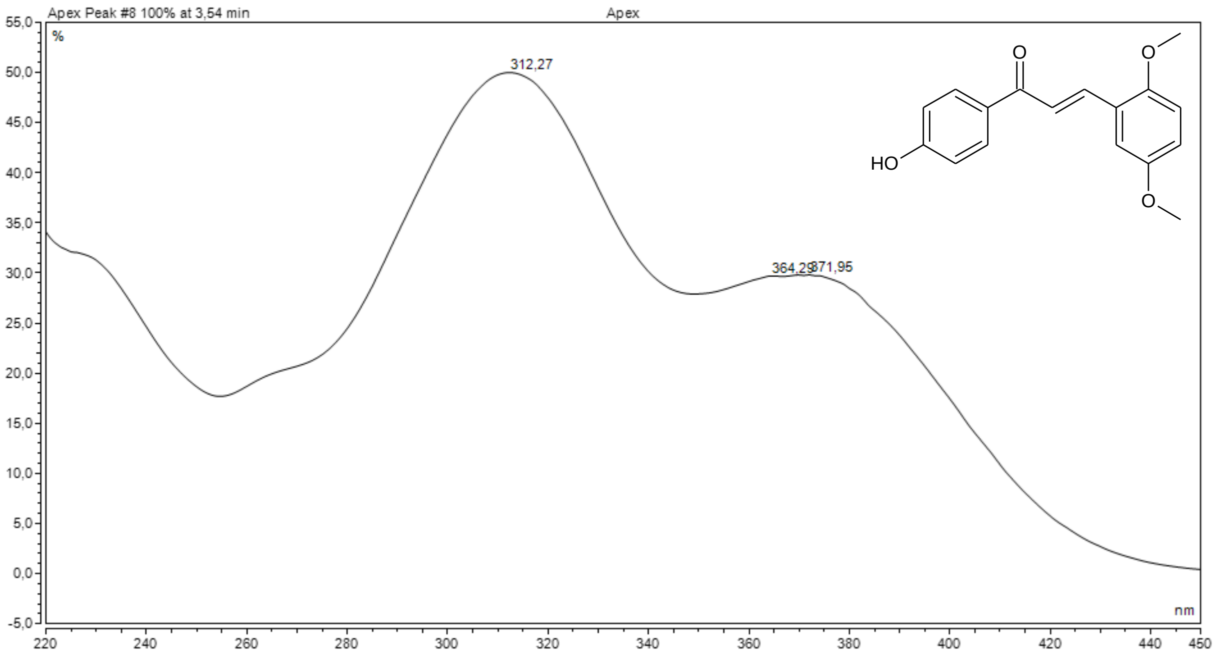

Figure S56. UV spectrum of *trans*-4′ -hydroxy-2,5-dimethoxychalcone (*trans*-**6**)

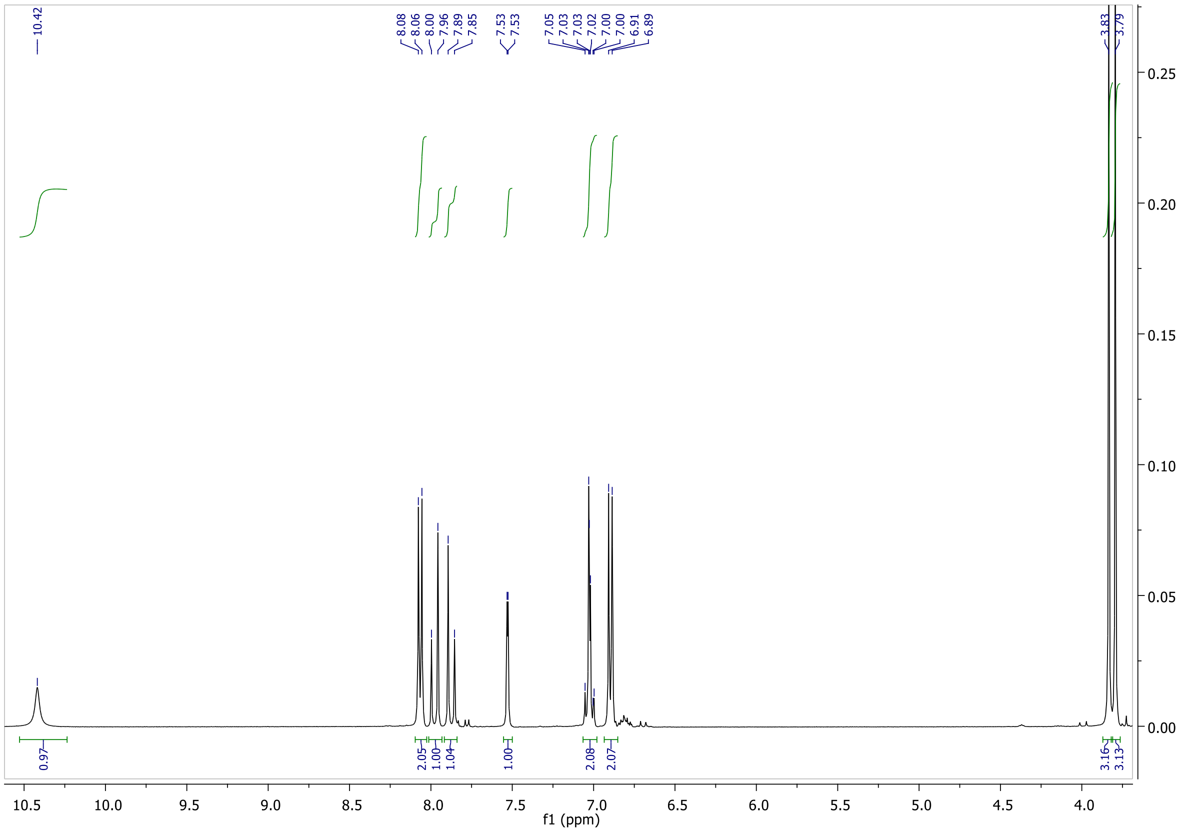

Figure S57. ^{1}H NMR spectrum of *trans*-4′ -hydroxy-2,5-dimethoxychalcone (*trans*-**6**)

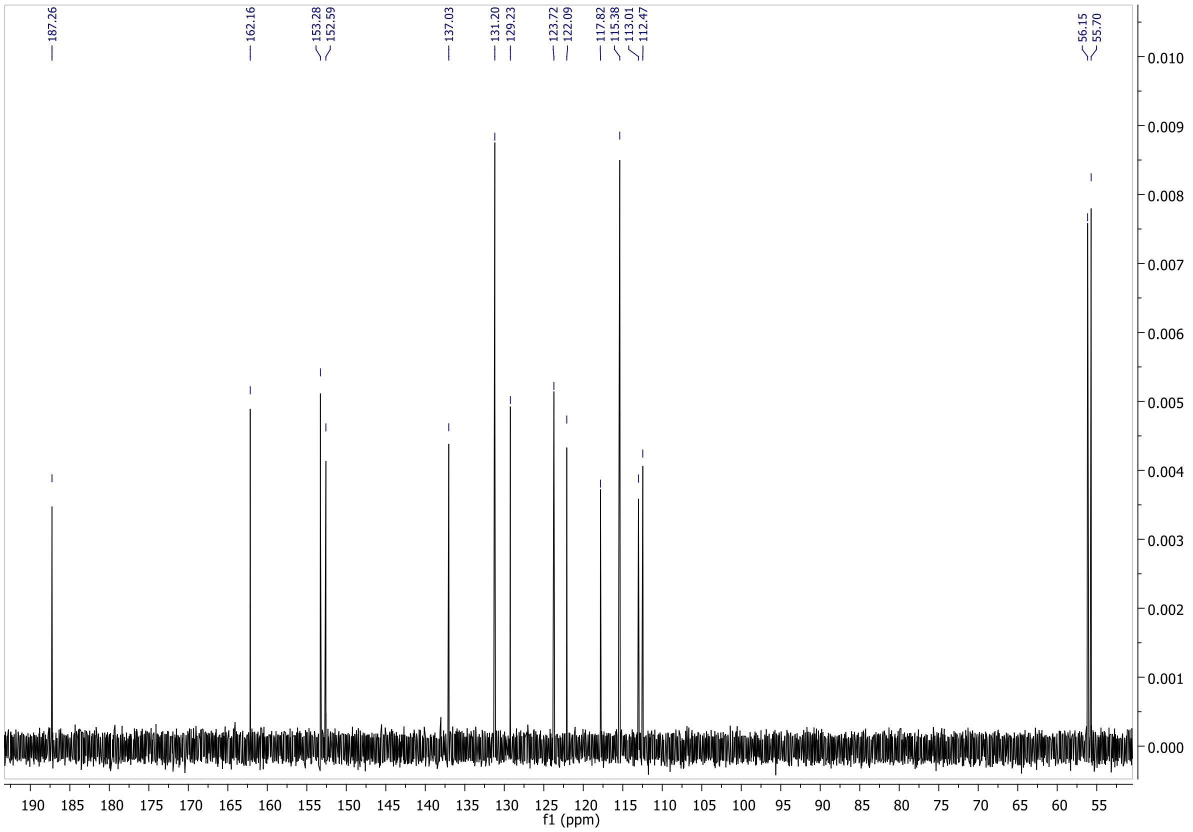

Figure S58. ^{13}C NMR spectrum of *trans*-4′ -hydroxy-2,5-dimethoxychalcone (*trans*-**6**)

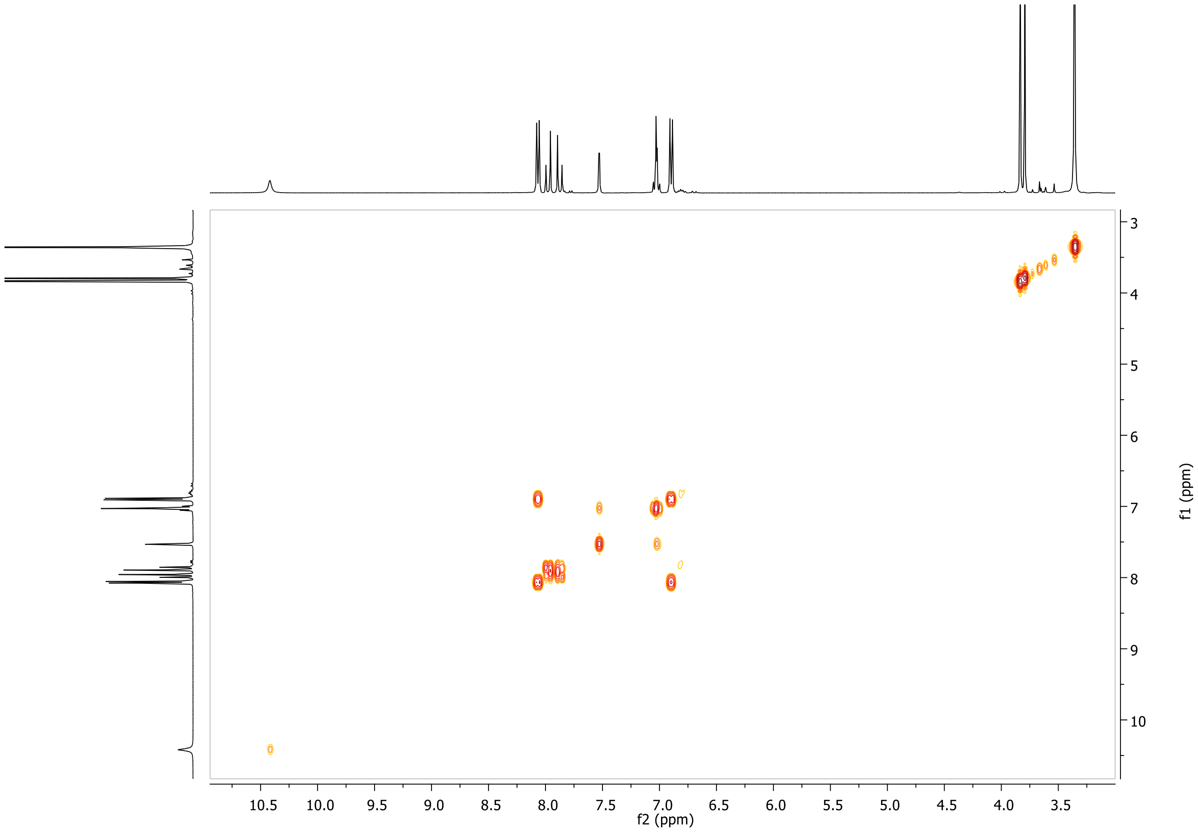

Figure S59. COSY NMR spectrum of *trans*-4′ -hydroxy-2,5-dimethoxychalcone (*trans*-**6**)

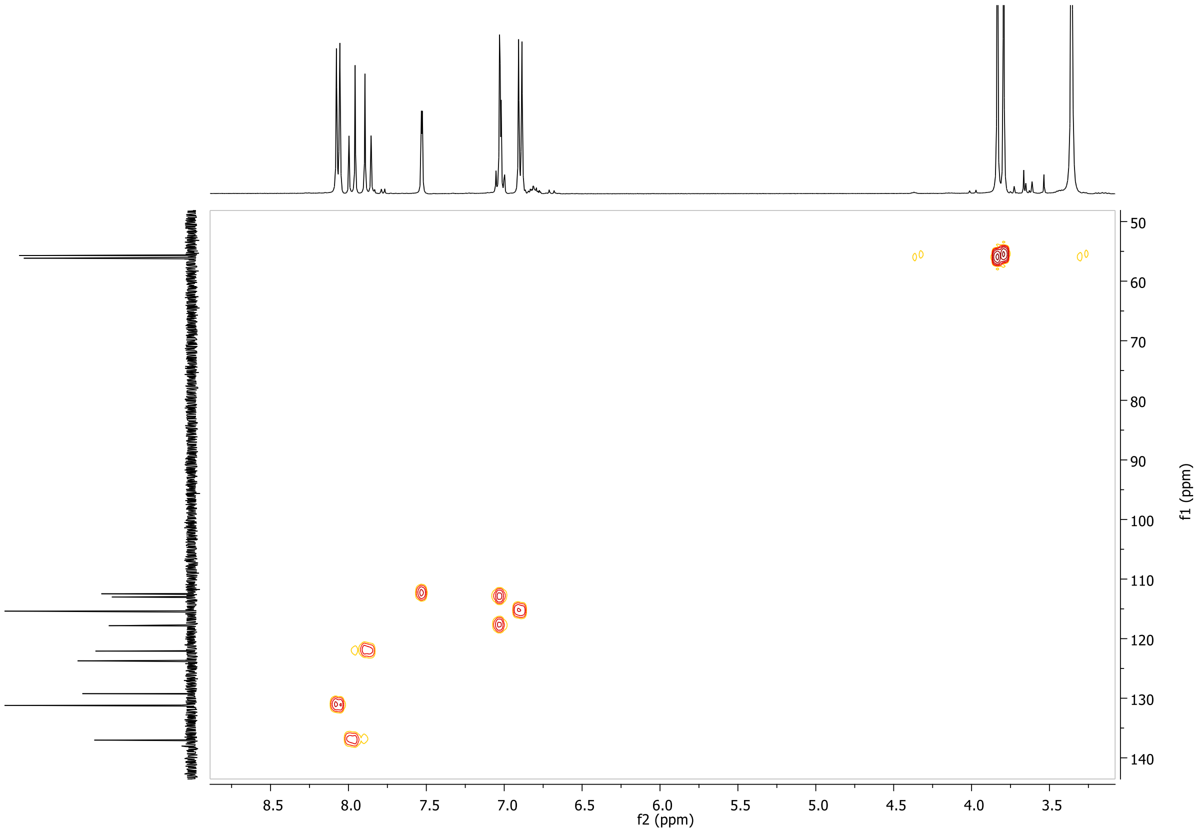

Figure S60. HSQC NMR spectrum of *trans*-4′ -hydroxy-2,5-dimethoxychalcone (*trans*-**6**)

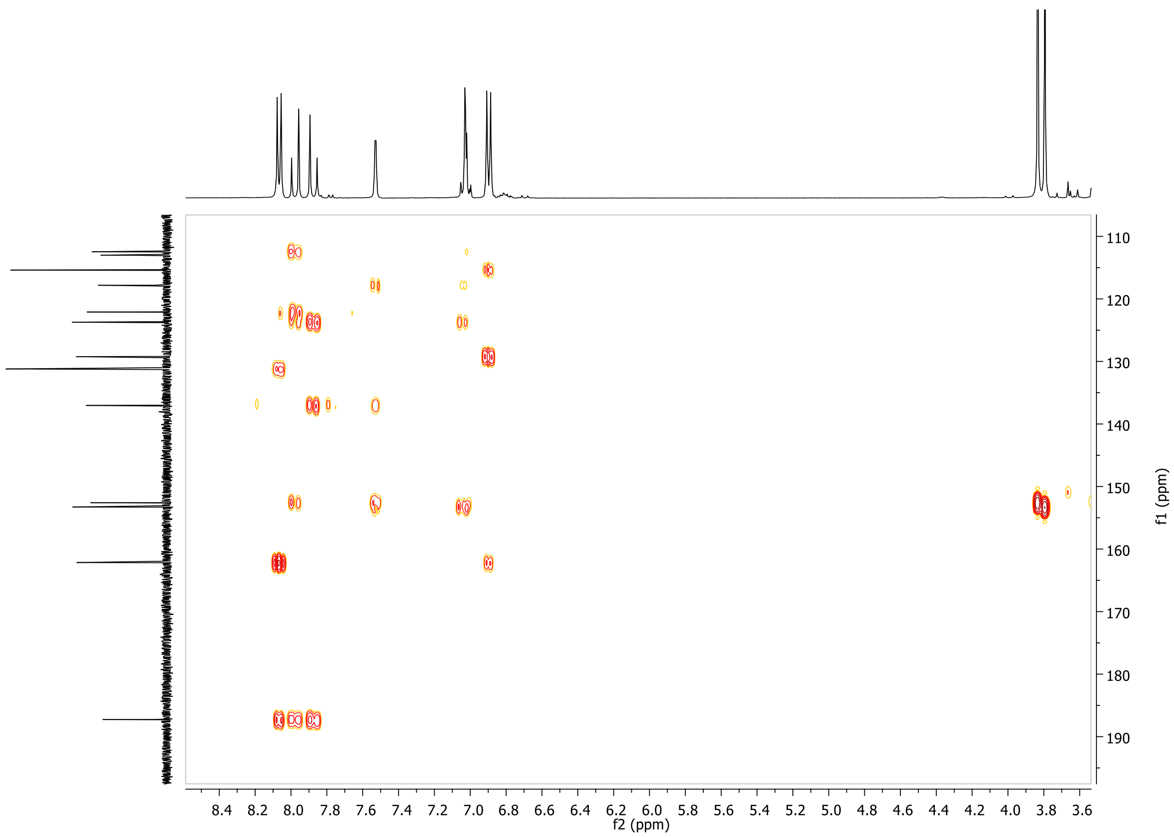

Figure S61. HMBC NMR spectrum of *trans*-4′ -hydroxy-2,5-dimethoxychalcone (*trans*-**6**)

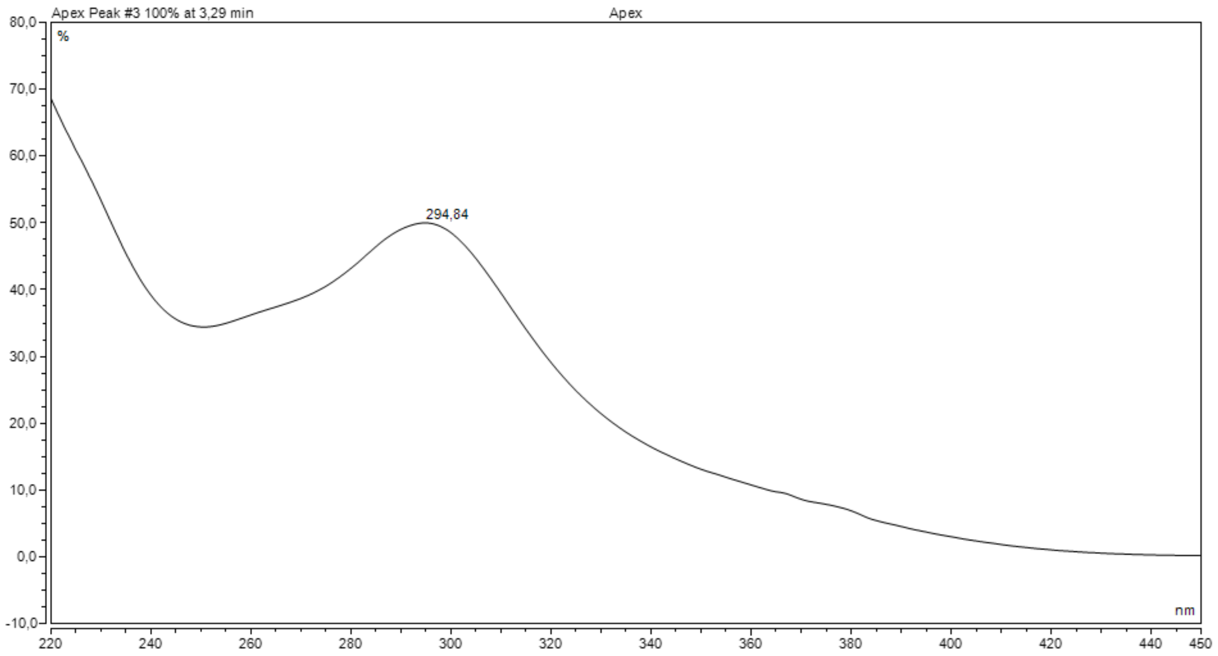

Figure S62. UV spectrum of *cis*-4′ -hydroxy-2,5-dimethoxychalcone (*cis*-**6**)

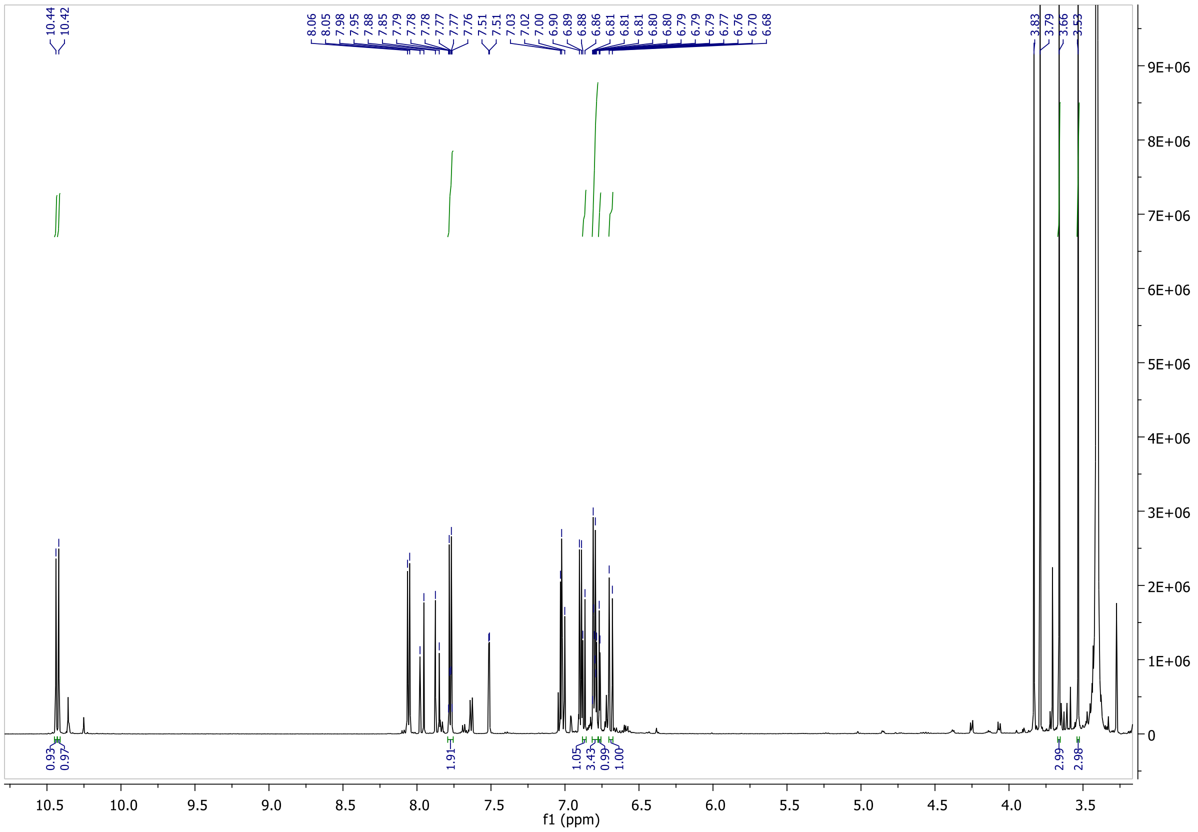

Figure S63. ^{1}H NMR spectrum of *cis*-4′ -hydroxy-2,5-dimethoxychalcone (*cis*-**6**)

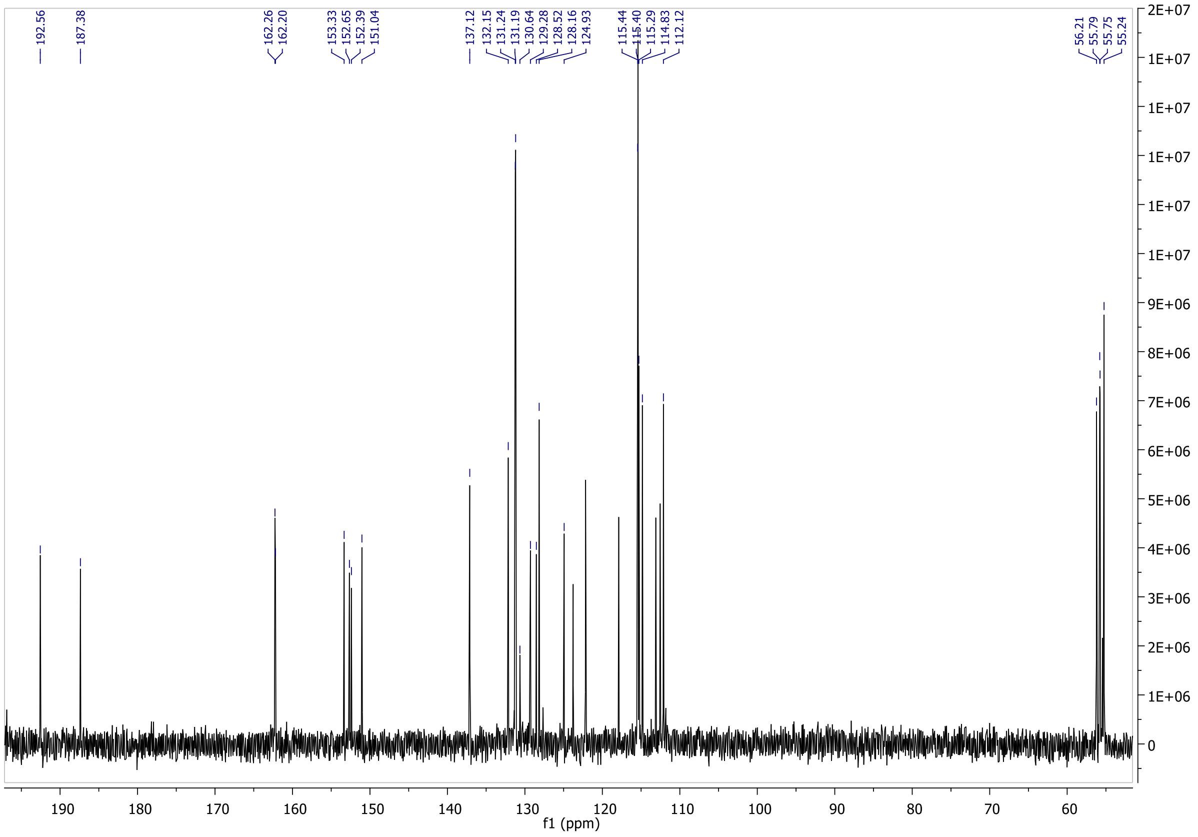

Figure S64. ^{13}C NMR spectrum of *cis*-4′ -hydroxy-2,5-dimethoxychalcone (*cis*-**6**)

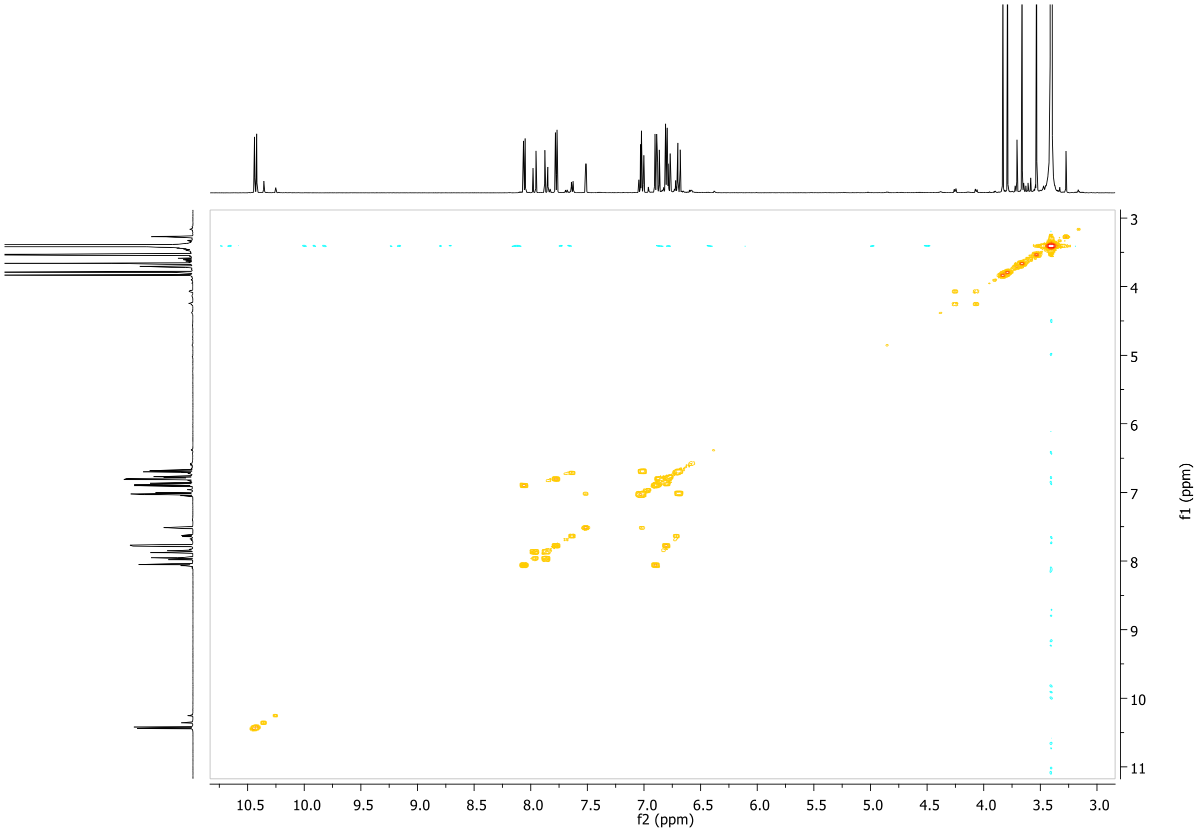

Figure S65. COSY NMR spectrum of *cis*-4′ -hydroxy-2,5-dimethoxychalcone (*cis*-**6**)

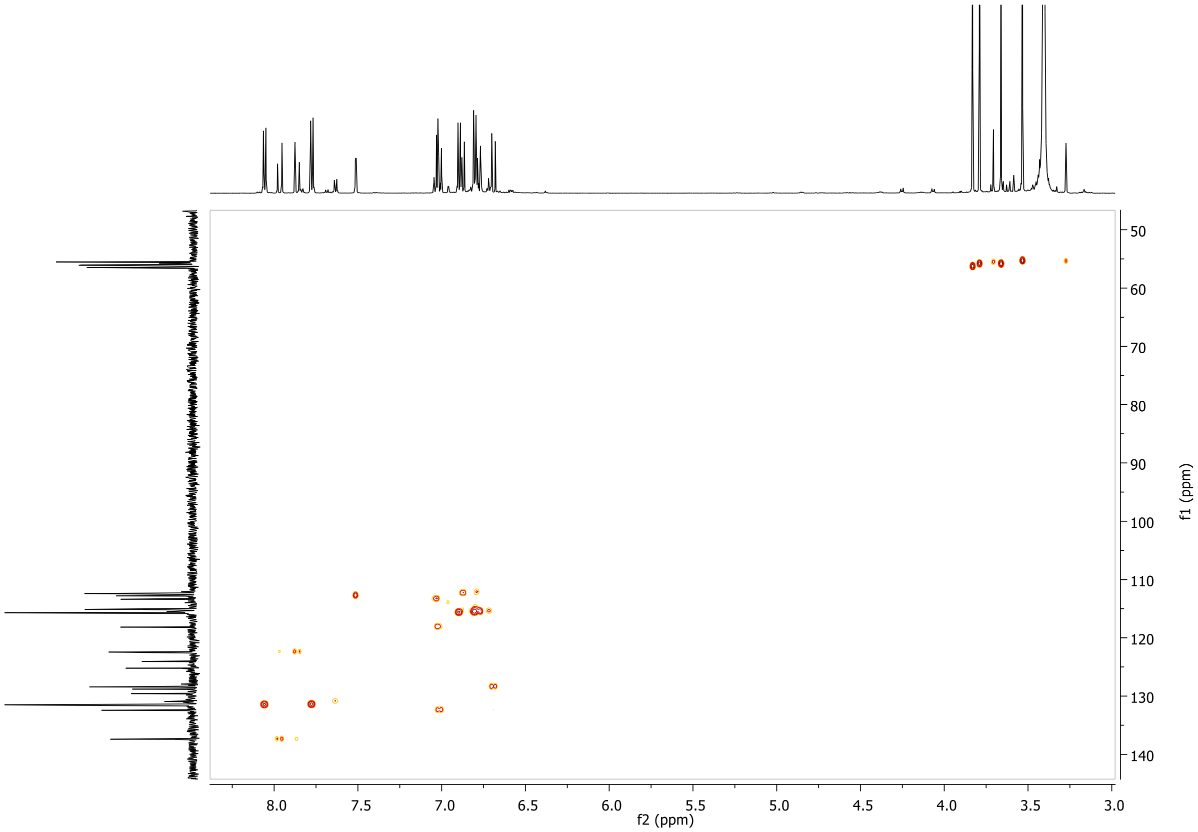

Figure S66. HSQC NMR spectrum of *cis*-4′ -hydroxy-2,5-dimethoxychalcone (*cis*-**6**)

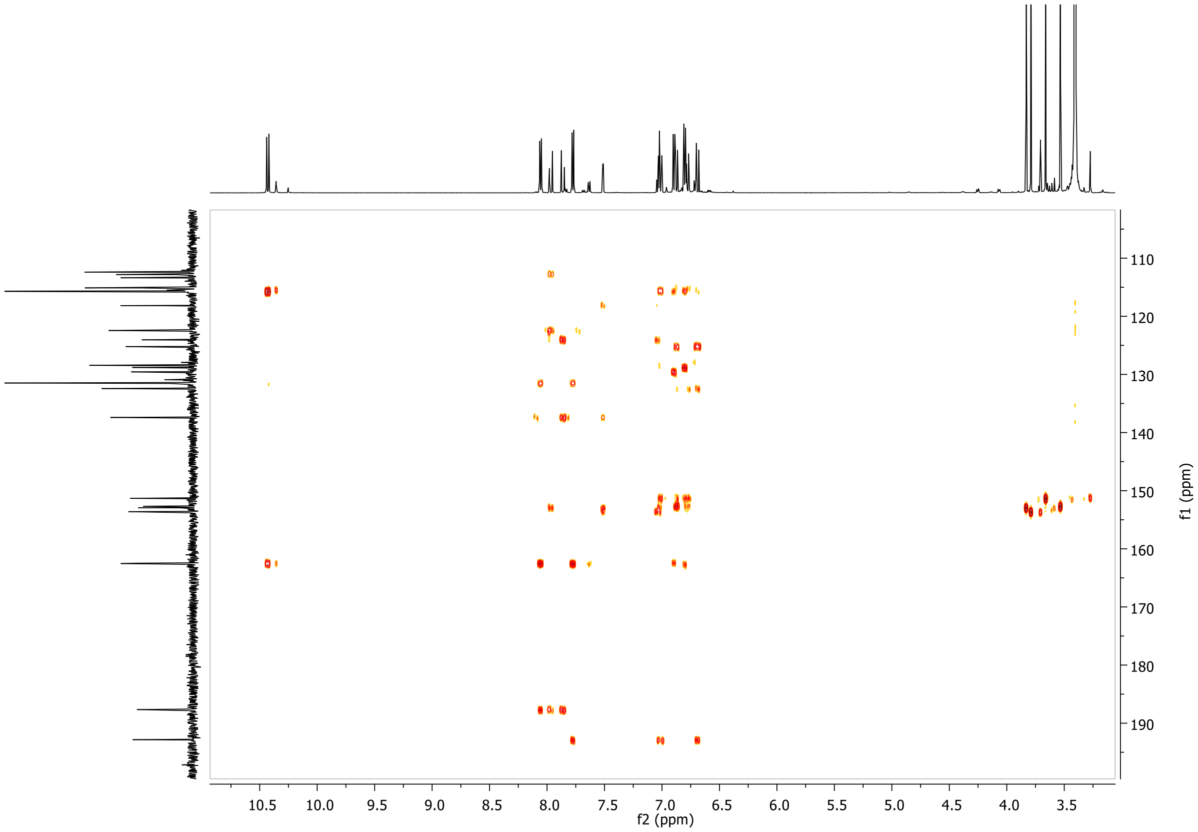

Figure S67. HMBC NMR spectrum of *cis*-4′ -hydroxy-2,5-dimethoxychalcone (*cis*-**6**)

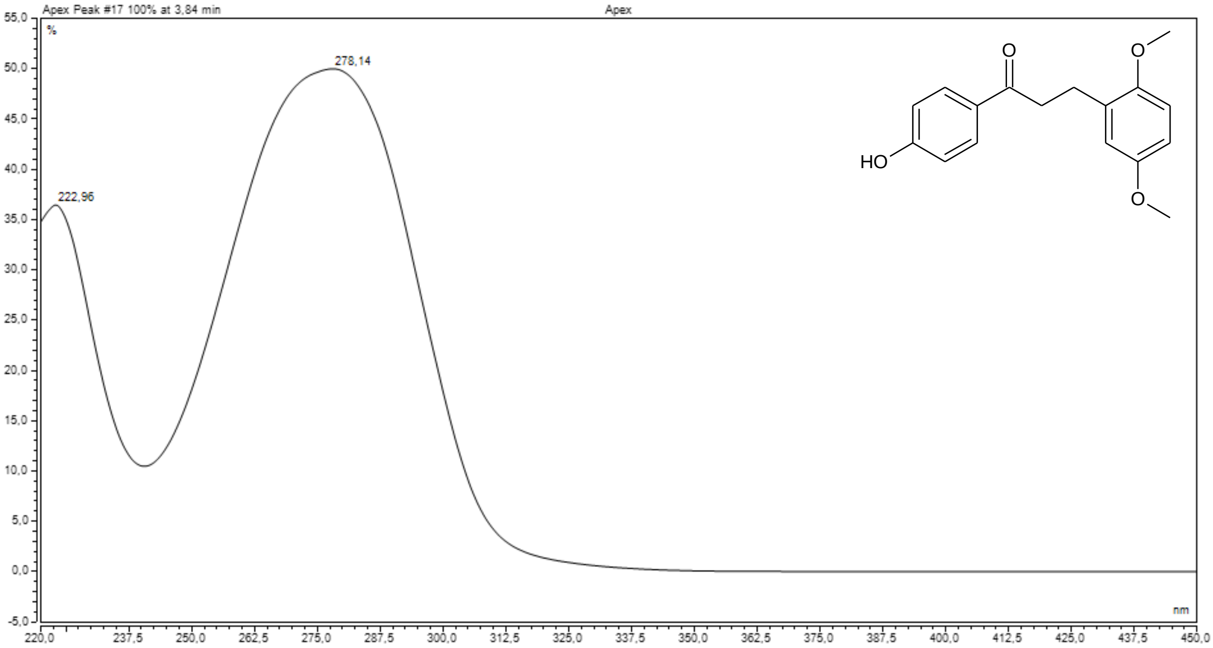

Figure S68. UV spectrum of 4′-hydroxy-2,5-dimethoxydihydrochalcone (**6a**)

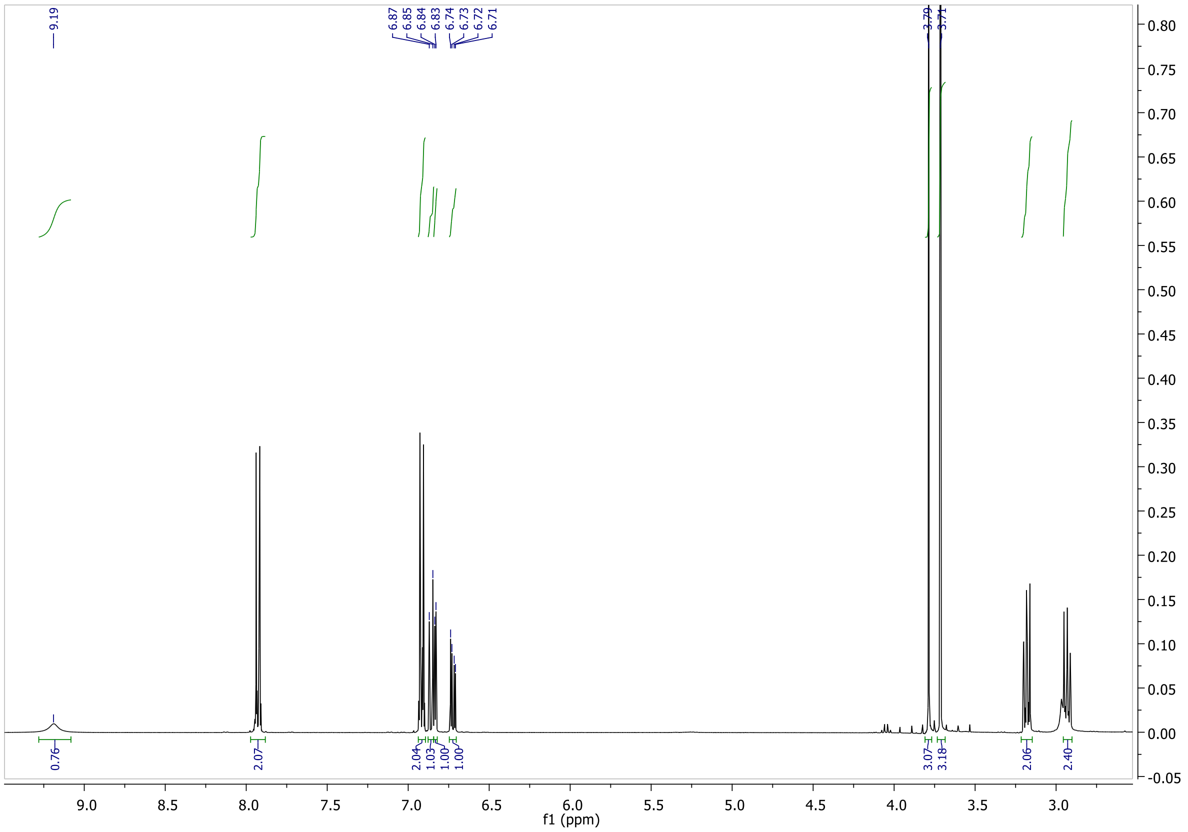

Figure S69. ^{1}H NMR spectrum of 4′-hydroxy-2,5-dimethoxydihydrochalcone (**6a**)

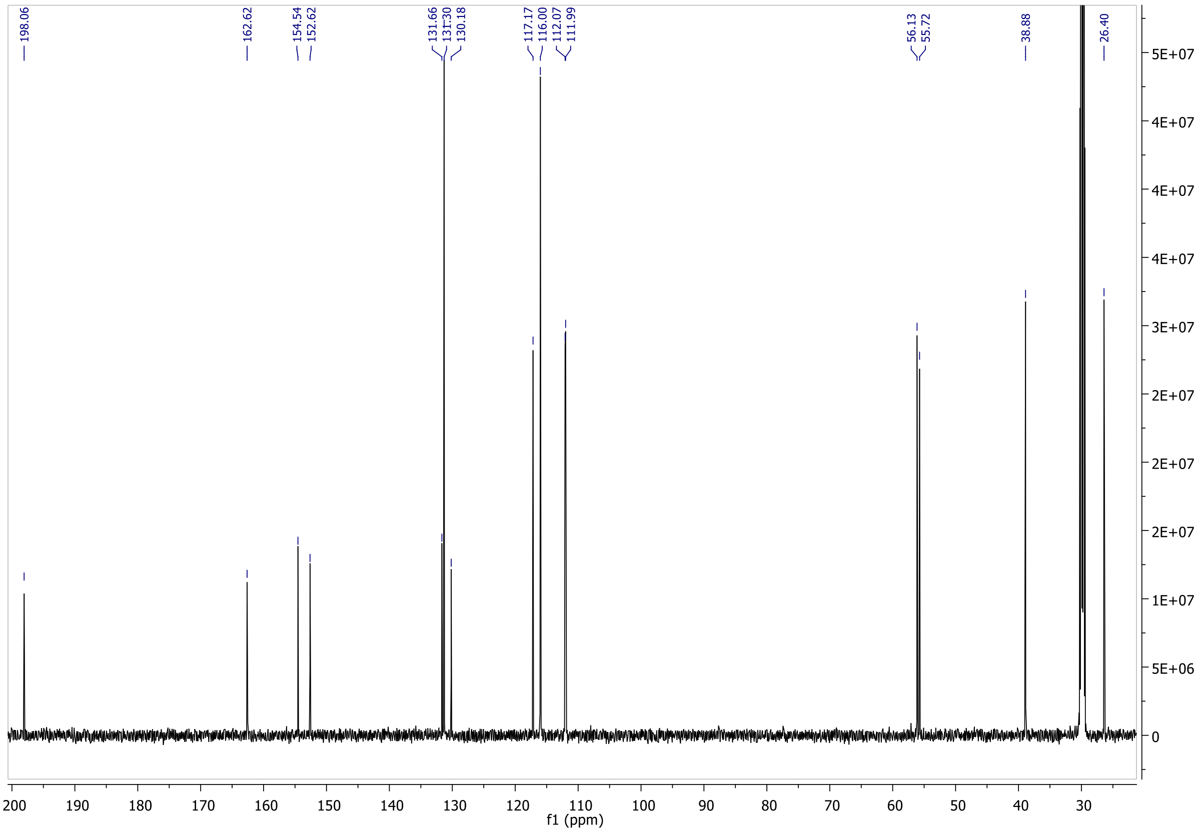

Figure S70. ^{13}C NMR spectrum of 4′-hydroxy-2,5-dimethoxydihydrochalcone (**6a**)

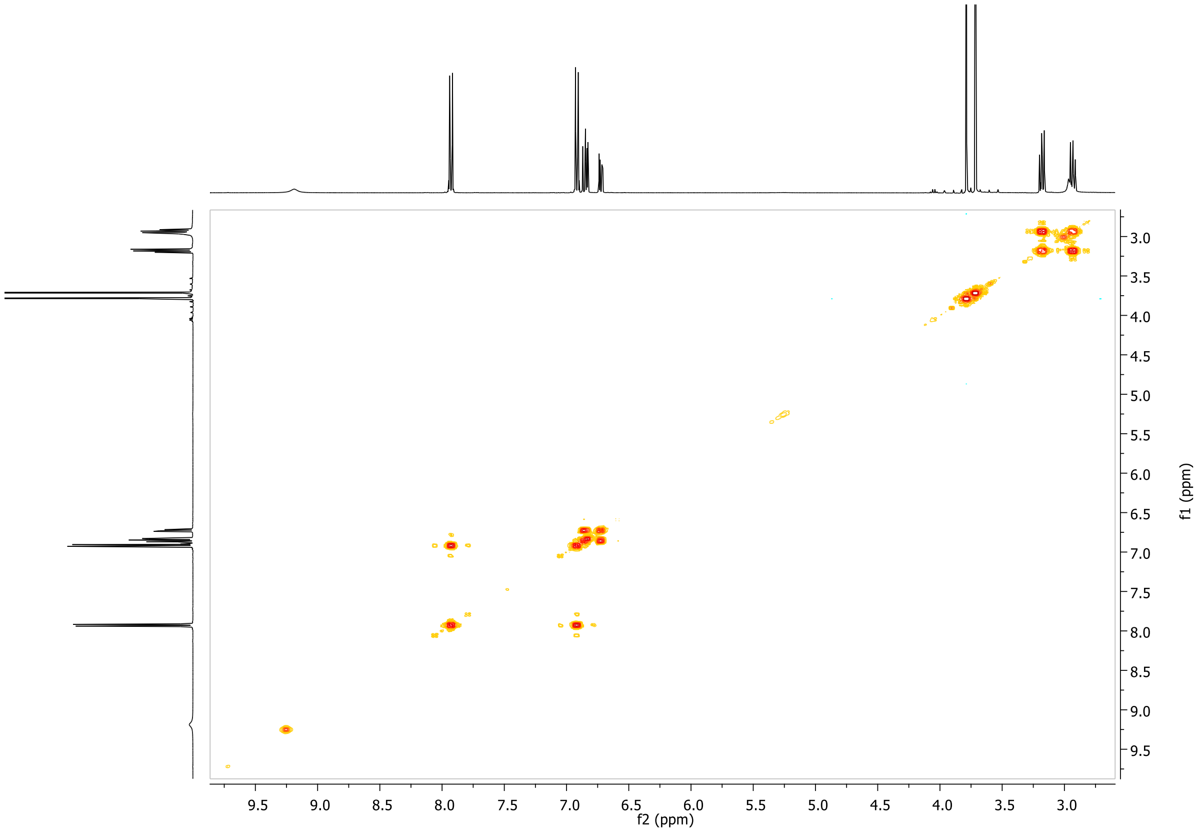

Figure S71. COSY NMR spectrum of 4′-hydroxy-2,5-dimethoxydihydrochalcone (**6a**)

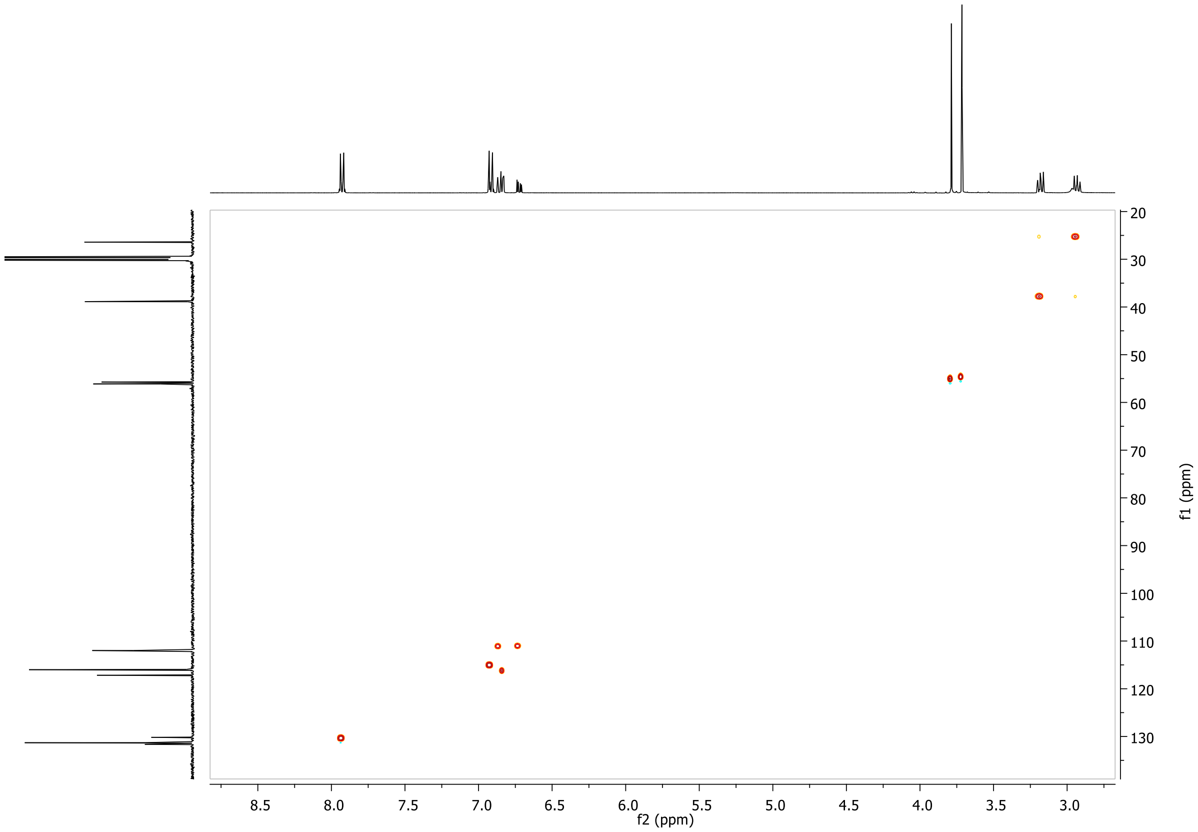

Figure S72. HSQC NMR spectrum of 4′-hydroxy-2,5-dimethoxydihydrochalcone (**6a**)

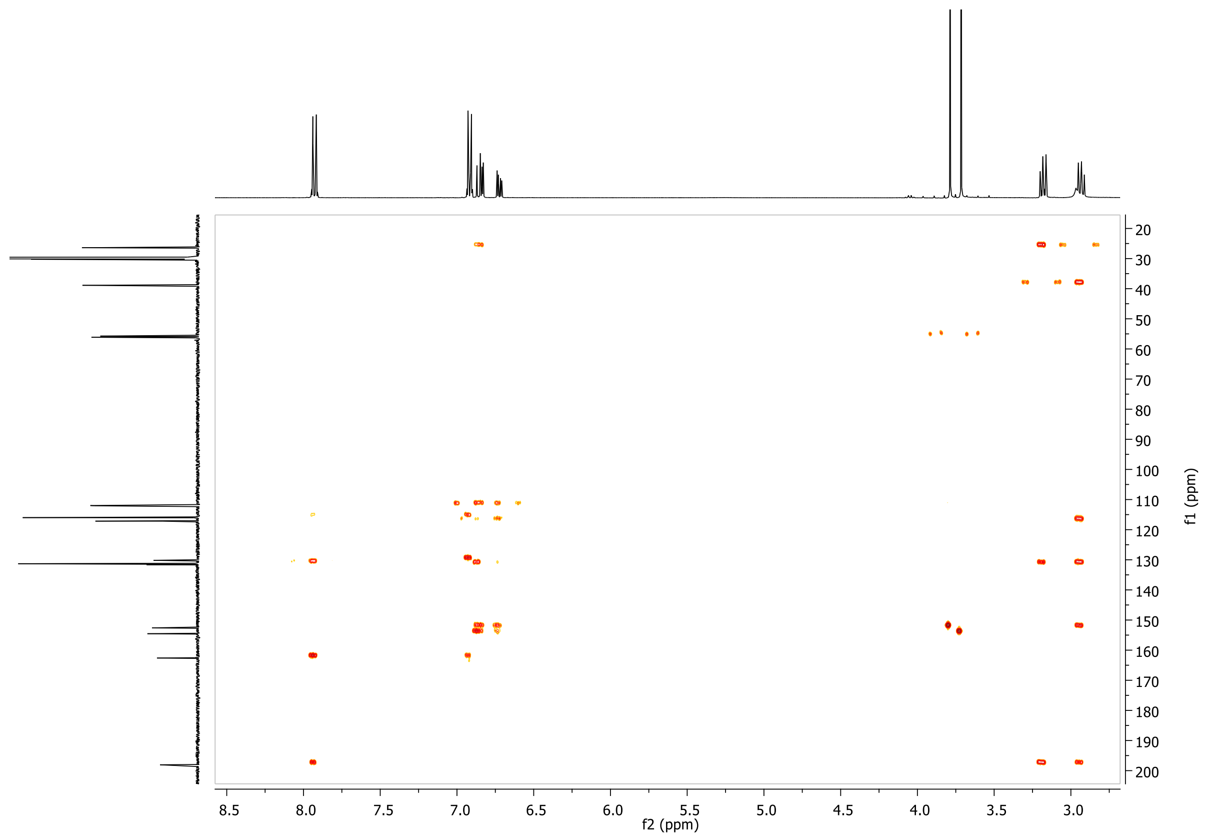

Figure S73. HMBC NMR spectrum of 4′-hydroxy-2,5-dimethoxydihydrochalcone (**6a**)

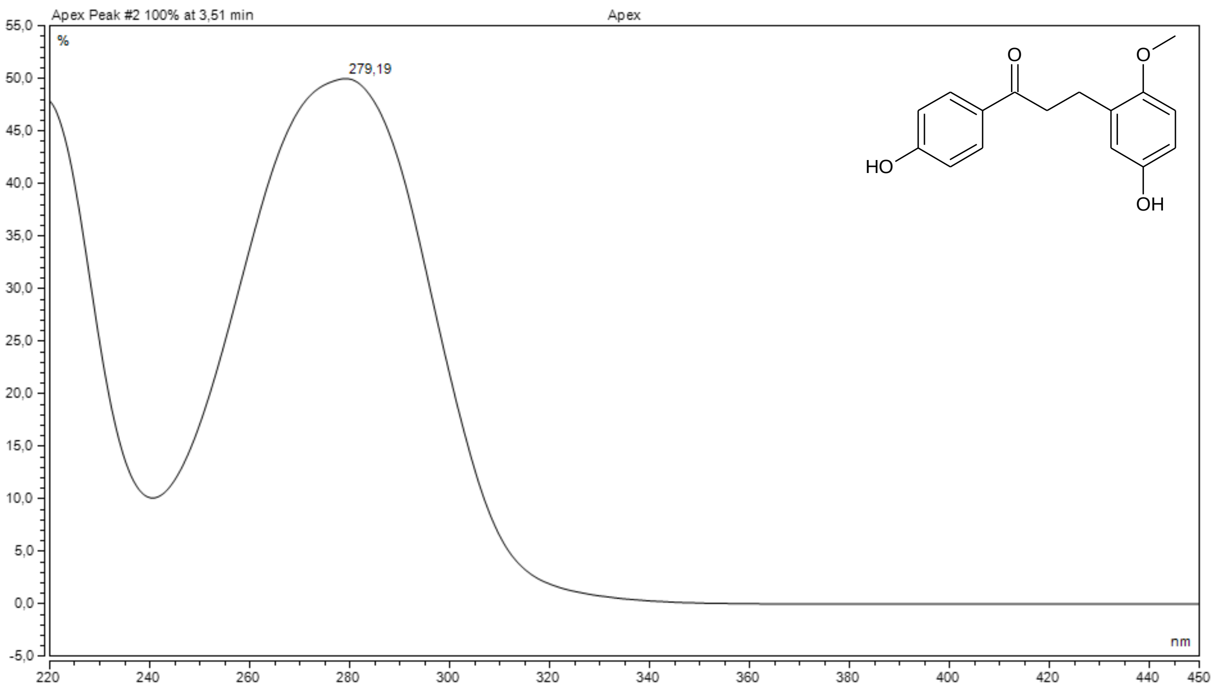

Figure S74. UV spectrum of 4′,5-dihydroxy-2-methoxydihydrochalcone (**6b**)

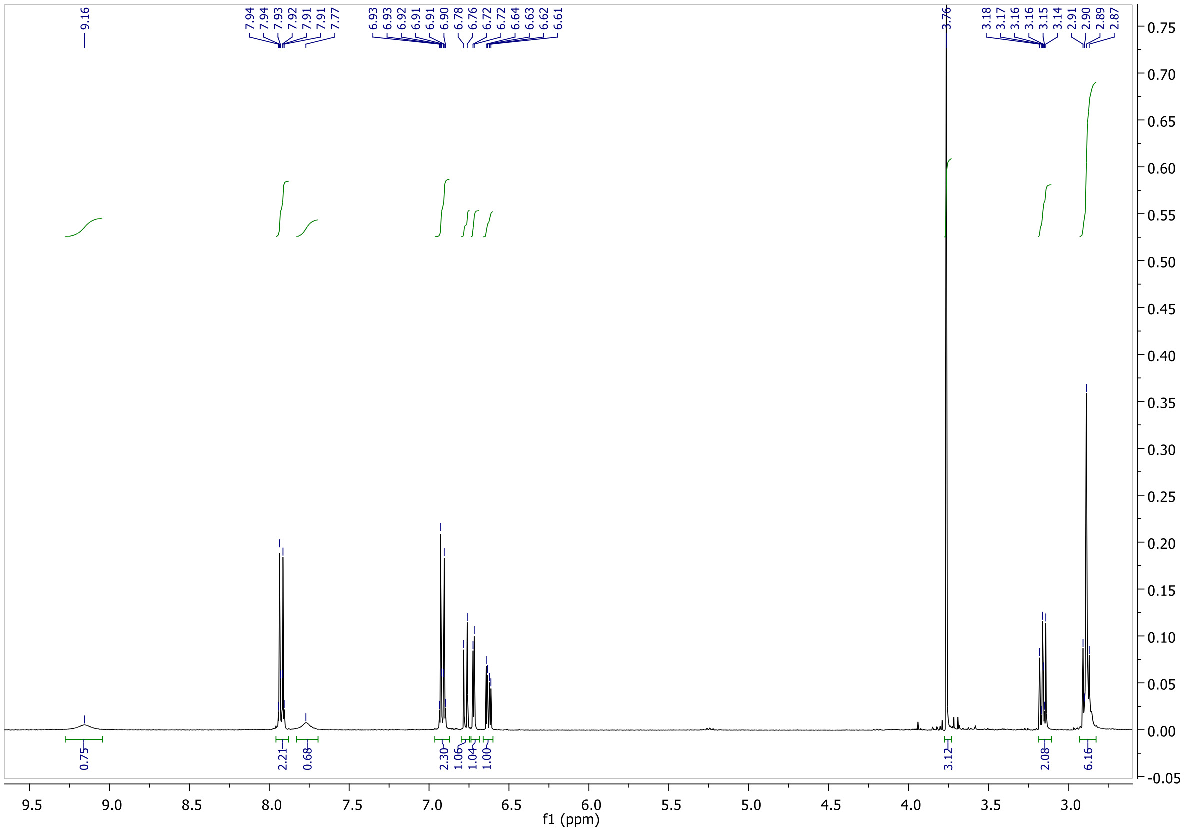

Figure S75. ^{1}H NMR spectrum of 4′,5-dihydroxy-2-methoxydihydrochalcone (**6b**)

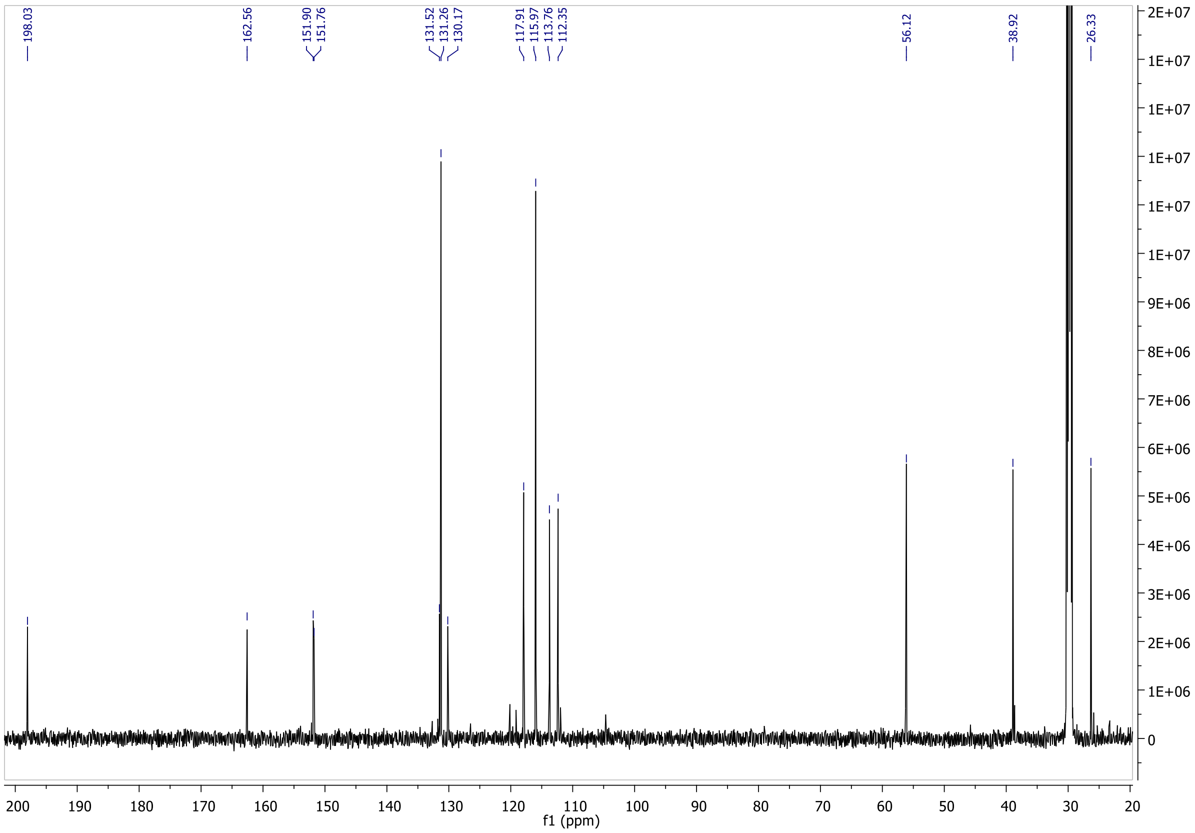

Figure S76. ^{13}C NMR spectrum of 4′,5-dihydroxy-2-methoxydihydrochalcone (**6b**)

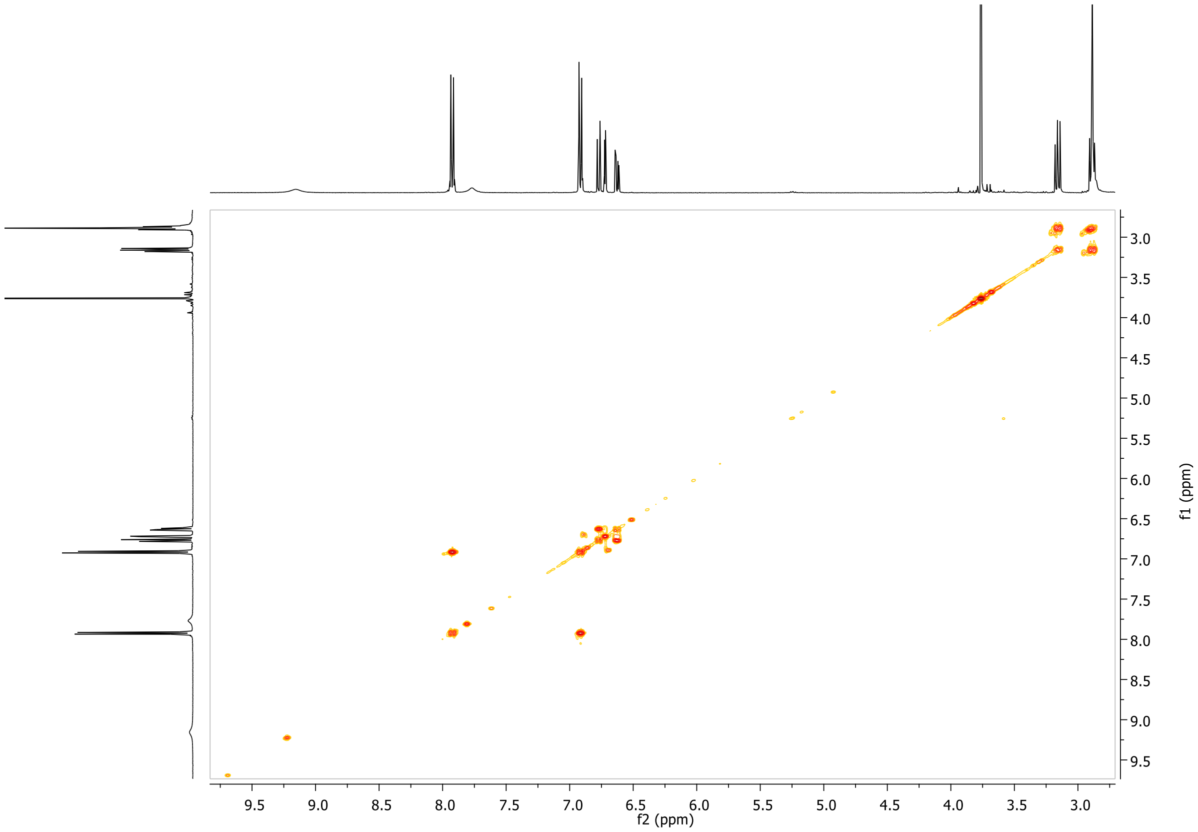

Figure S77. COSY NMR spectrum of 4′,5-dihydroxy-2-methoxydihydrochalcone (**6b**)

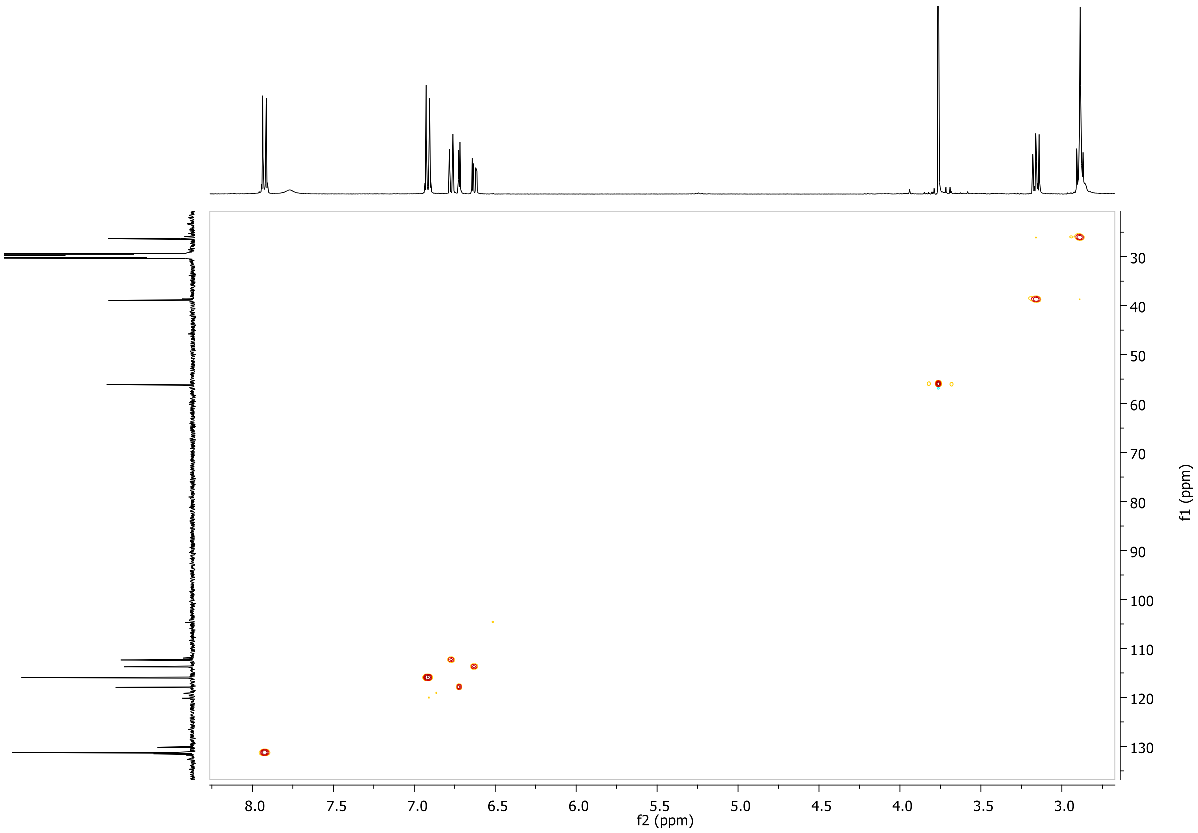

Figure S78. HMQC NMR spectrum of 4′,5-dihydroxy-2-methoxydihydrochalcone (**6b**)

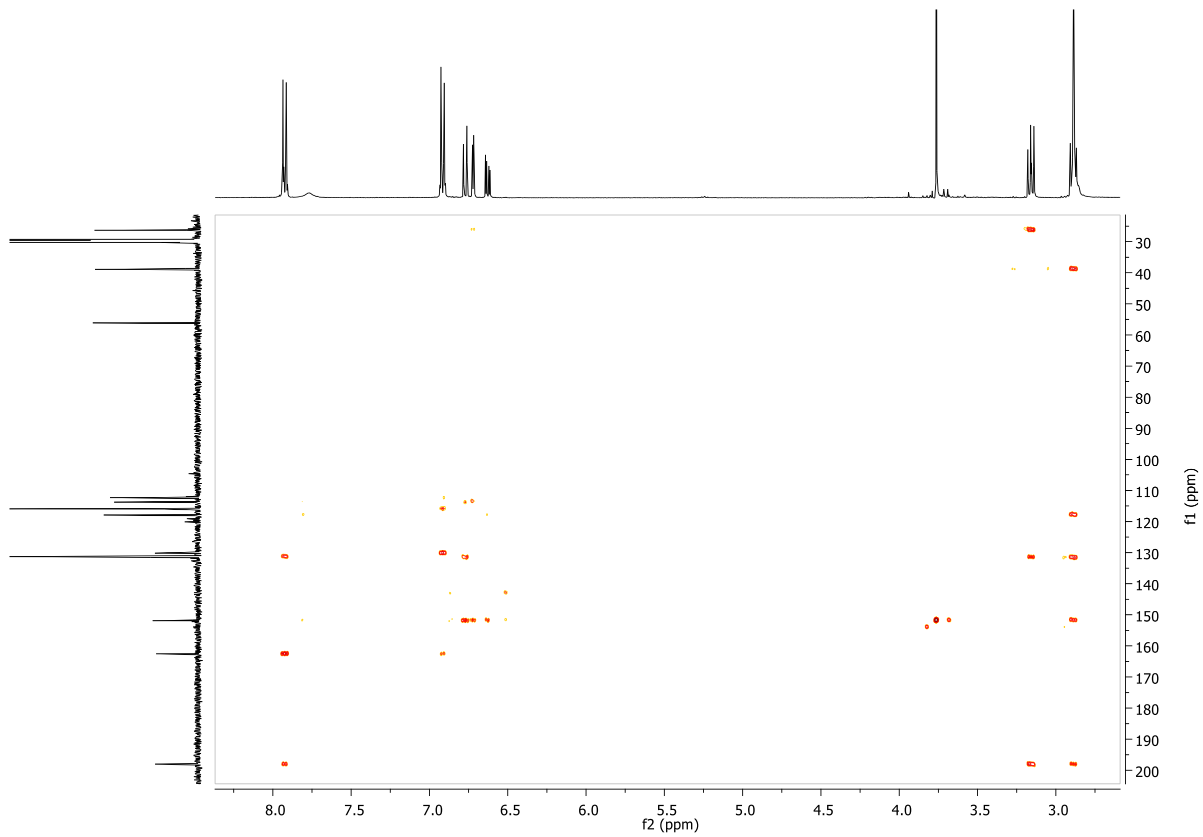

Figure S79. HMBC NMR spectrum of 4′,5-dihydroxy-2-methoxydihydrochalcone (**6b**)

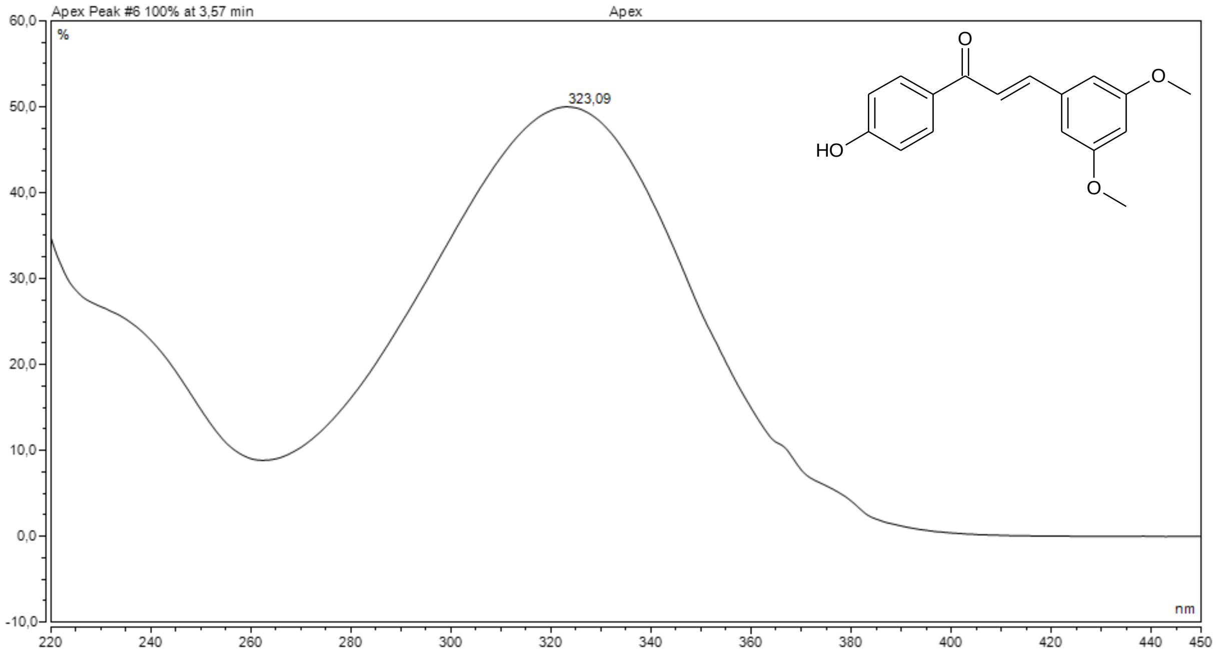

Figure S80 UV spectrum of *trans*-4′-hydroxy-3,5-dimethoxychalcone (*trans*-**7**)

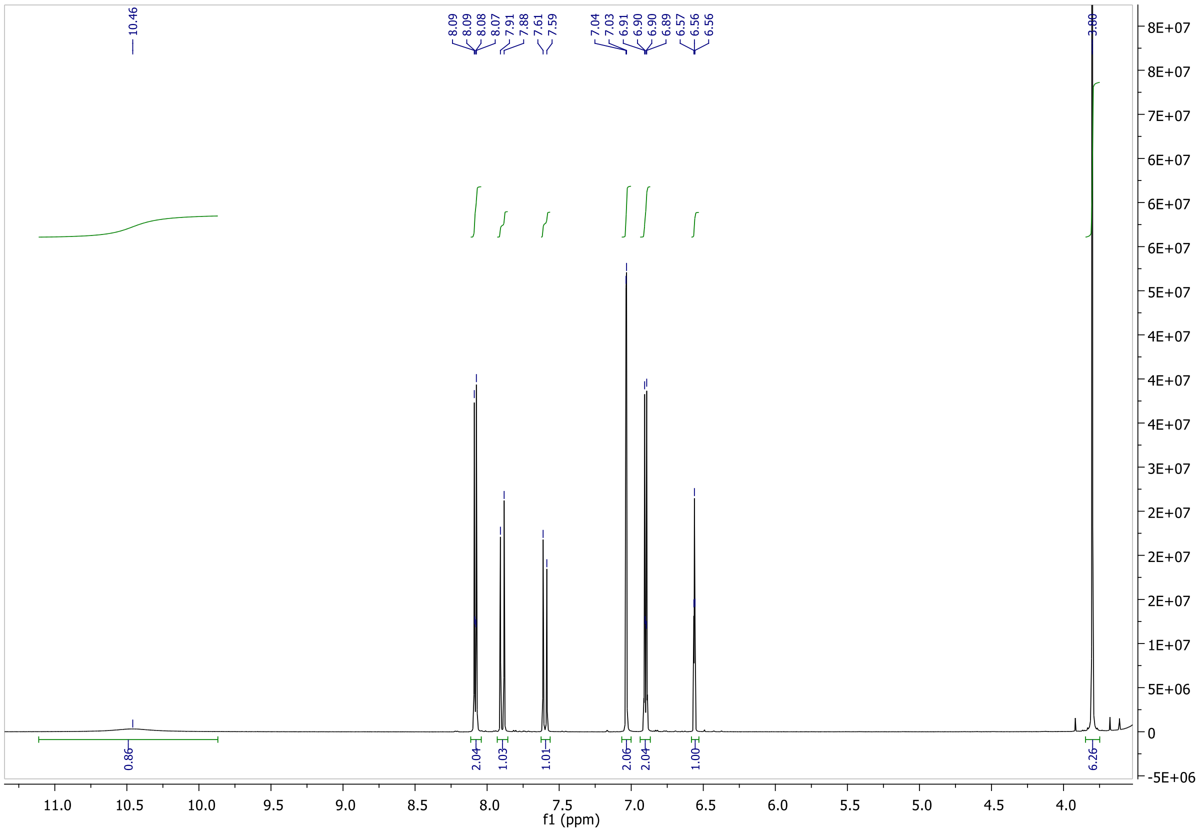

Figure S81. ^{1}H NMR spectrum of *trans*-4′-hydroxy-3,5-dimethoxychalcone (*trans*-**7**)

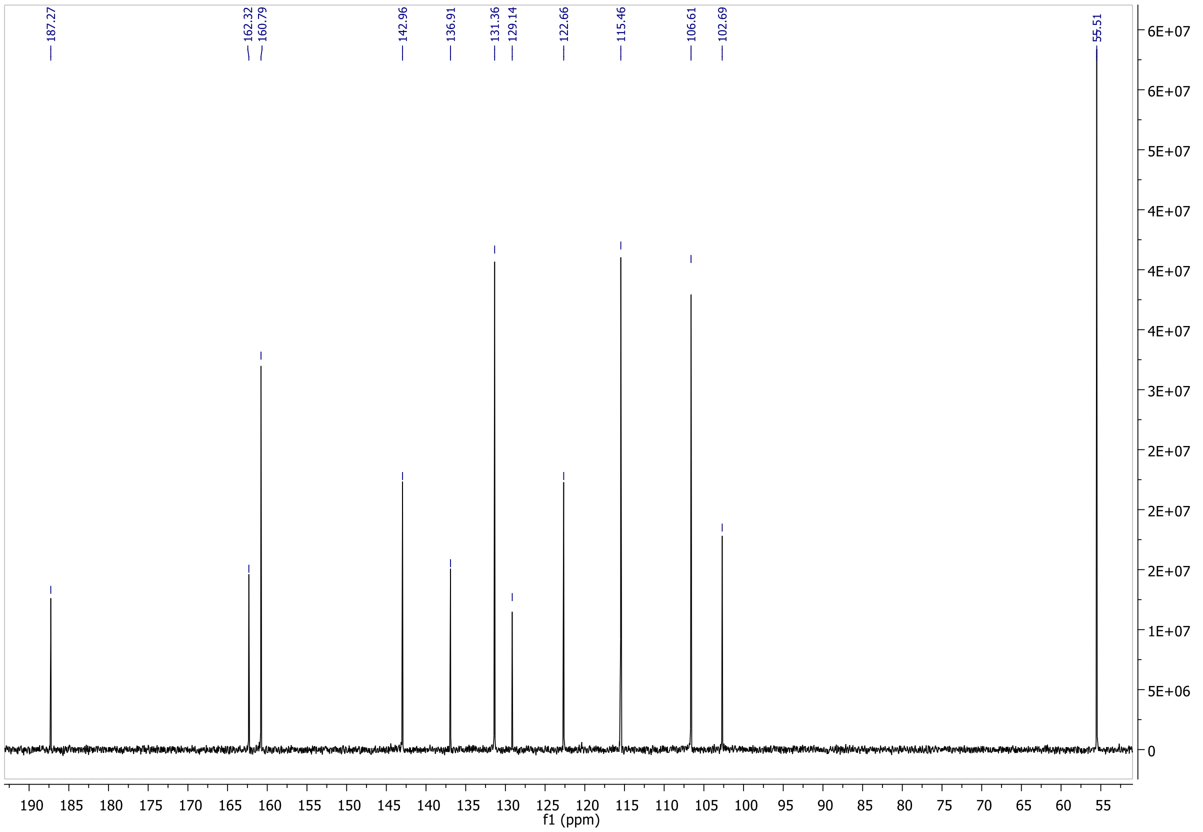

Figure S82. ^{13}C NMR spectrum of *trans*-4′-hydroxy-3,5-dimethoxychalcone (*trans*-**7**)

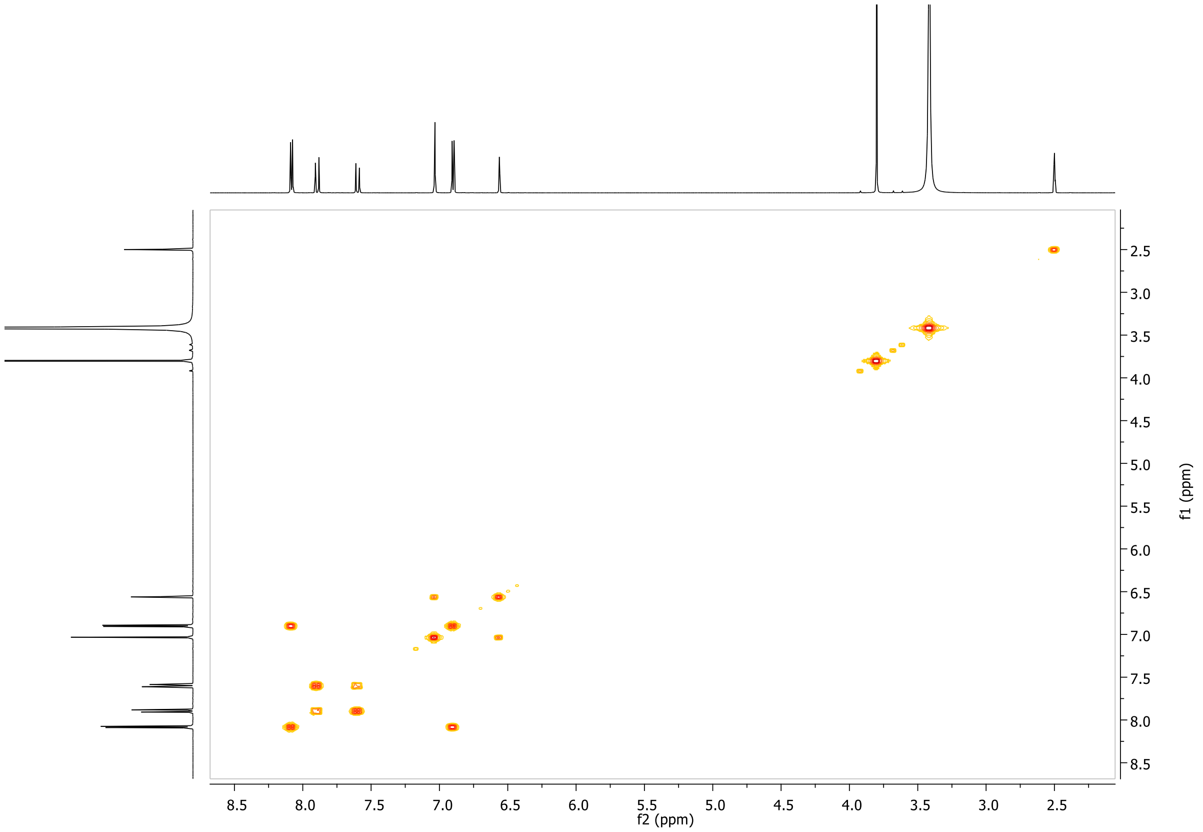

Figure S83. COSY NMR spectrum of *trans*-4′-hydroxy-3,5-dimethoxychalcone (*trans*-**7**)

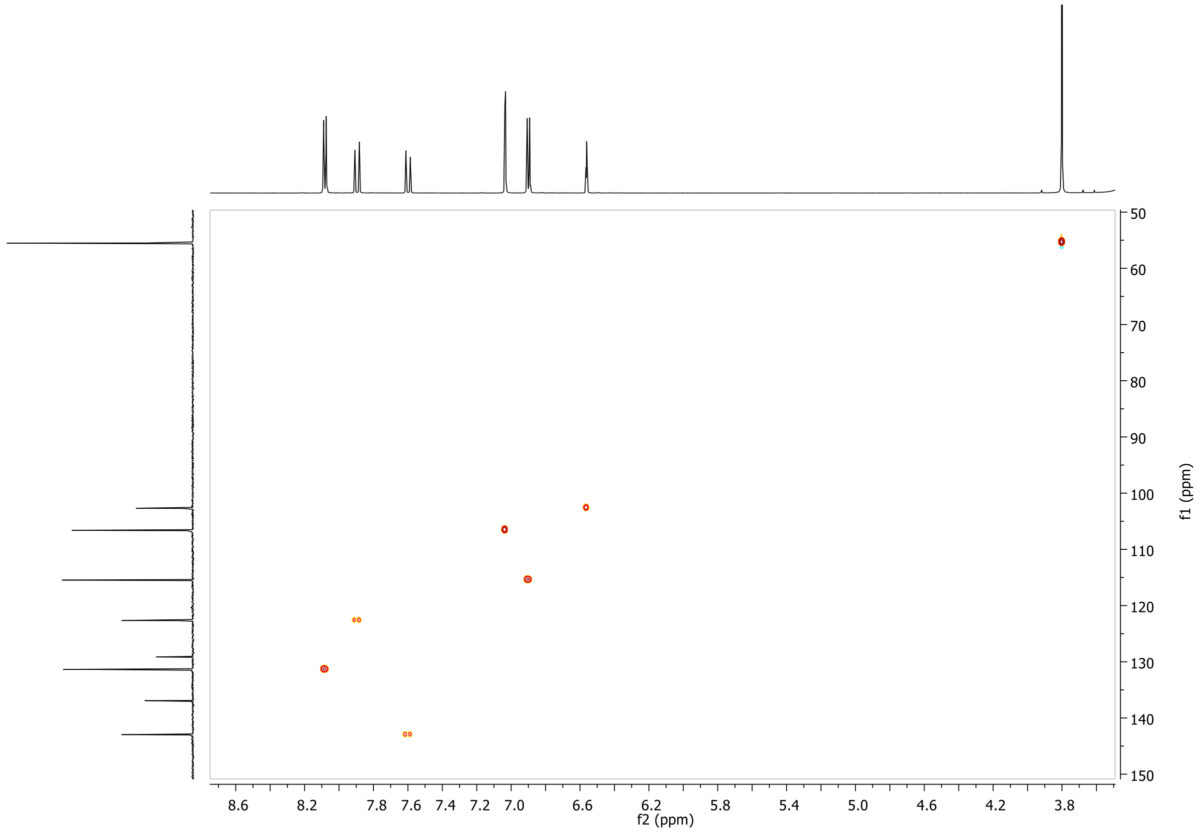

Figure S84. HSQC NMR spectrum of *trans*-4′-hydroxy-3,5-dimethoxychalcone (*trans*-**7**)

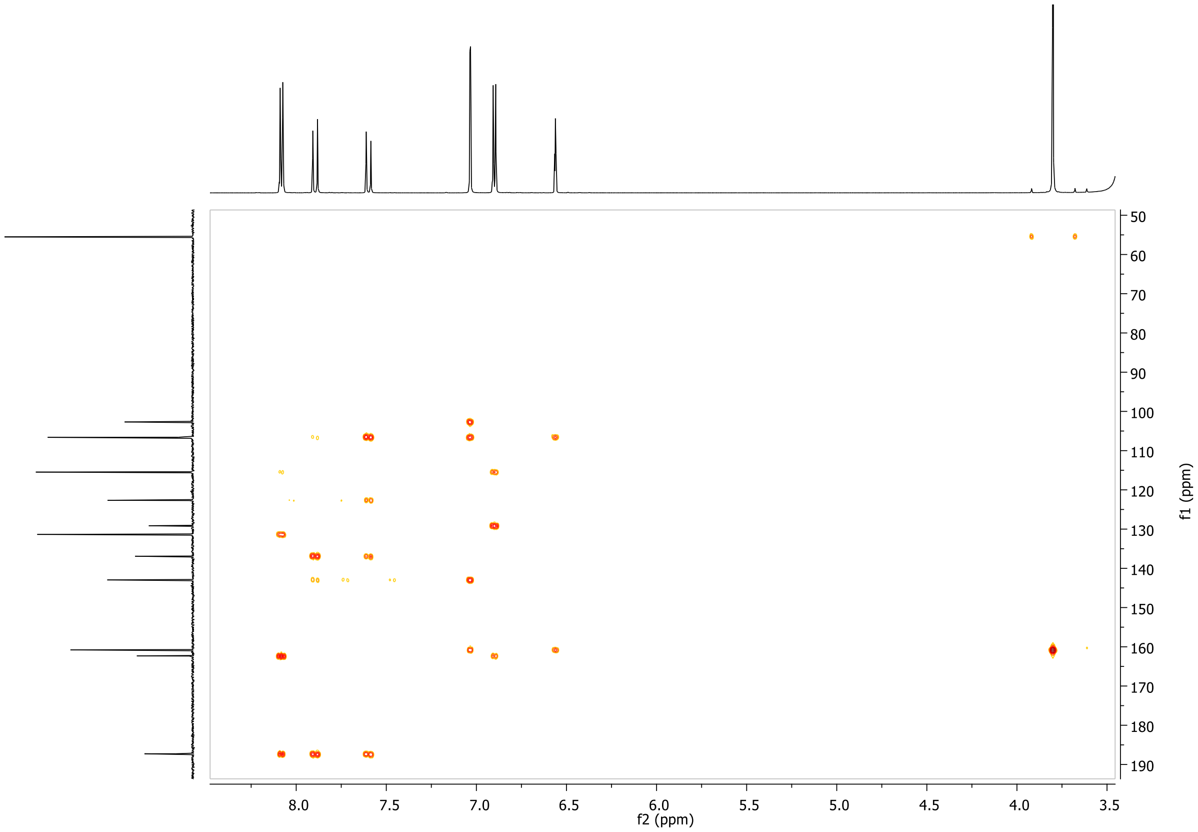

Figure S85. HMBC NMR spectrum of *trans*-4′-hydroxy-3,5-dimethoxychalcone (*trans*-**7**)

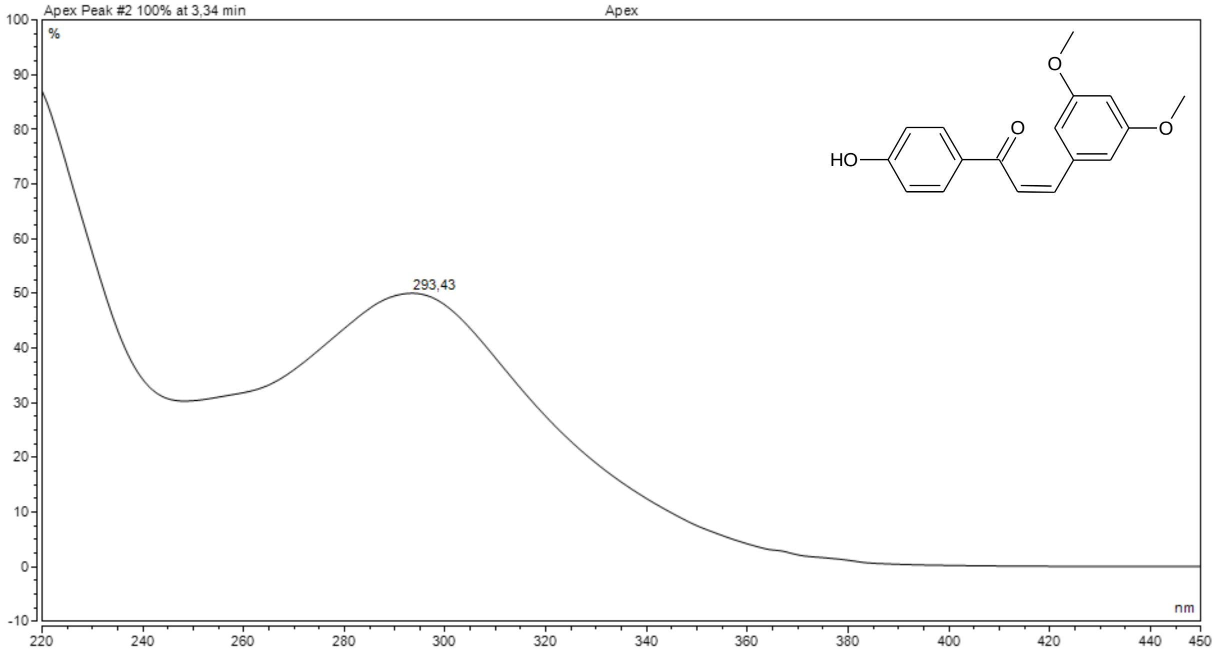

Figure S86. UV spectrum of *cis*-4′-hydroxy-3,5-dimethoxychalcone (*cis*-**7**)

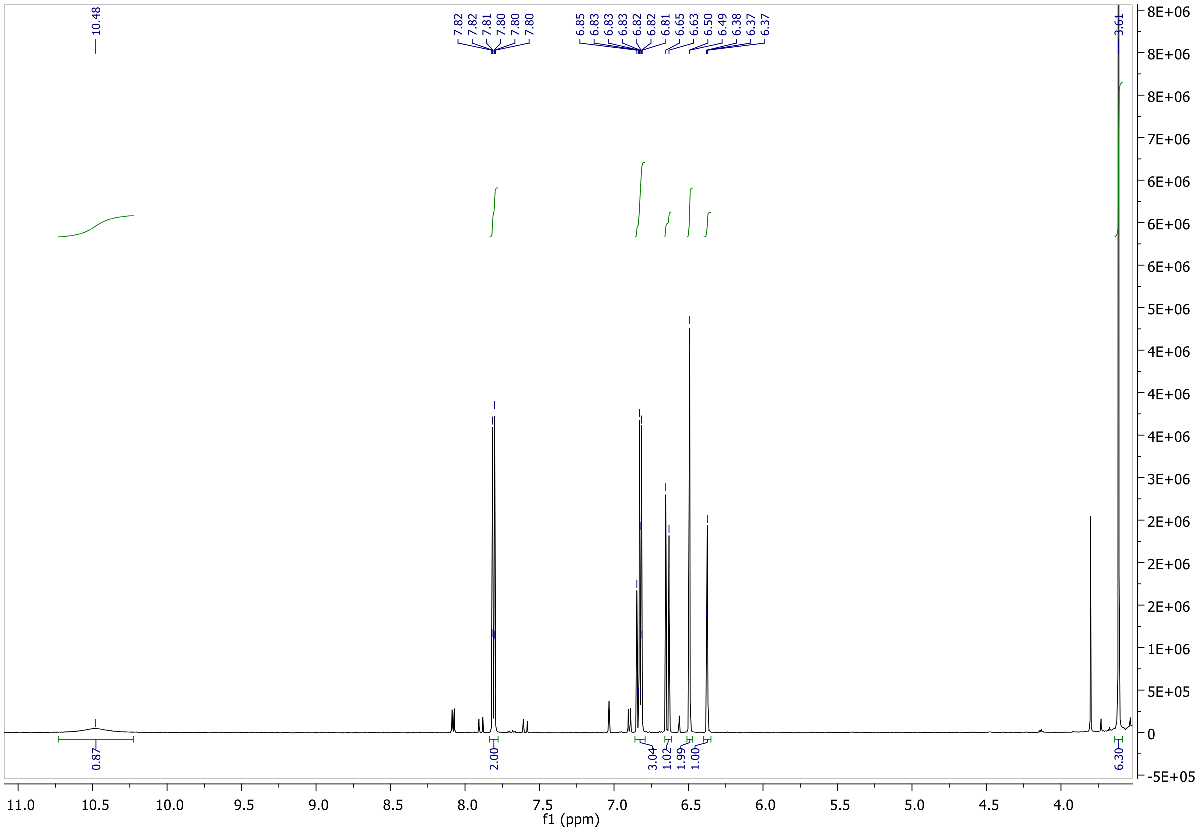

Figure S87. ^{1}H NMR spectrum of *cis*-4′-hydroxy-3,5-dimethoxychalcone (*cis*-**7**)

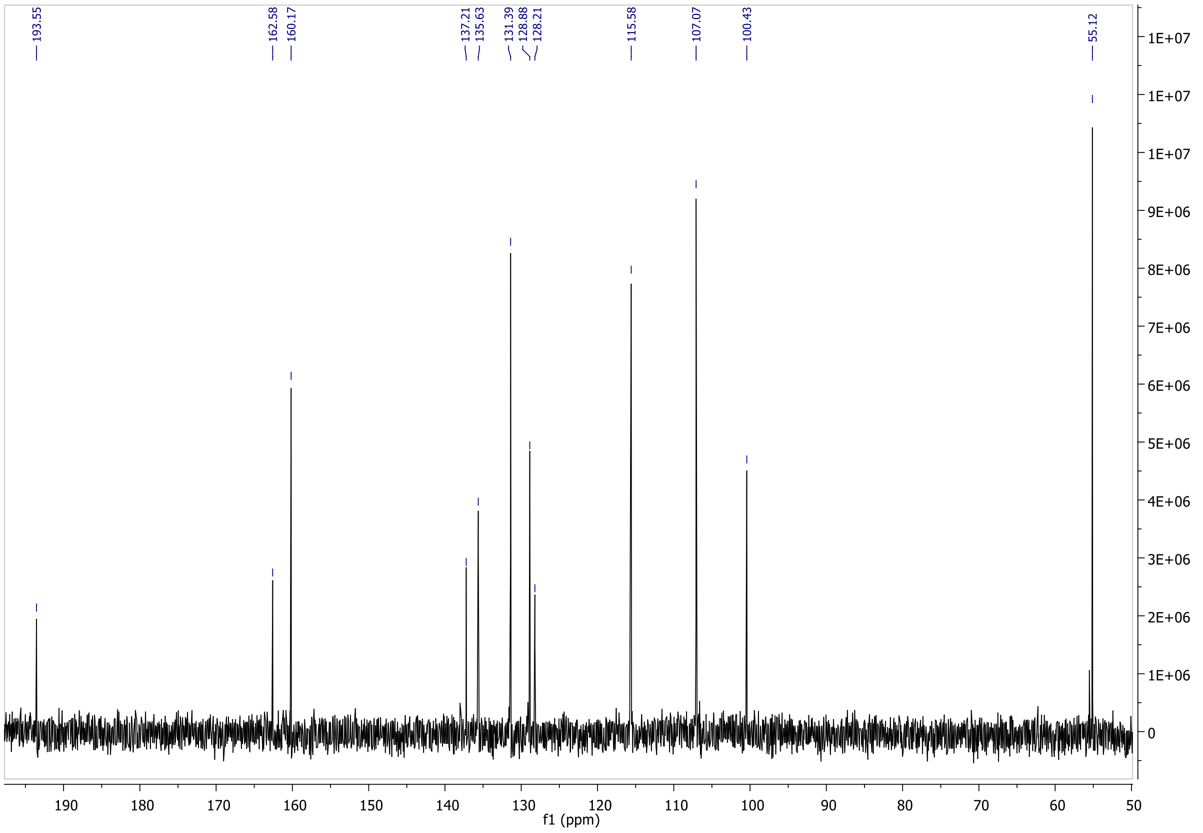

Figure S88. ^{13}C NMR spectrum of *cis*-4′-hydroxy-3,5-dimethoxychalcone (*cis*-**7**)

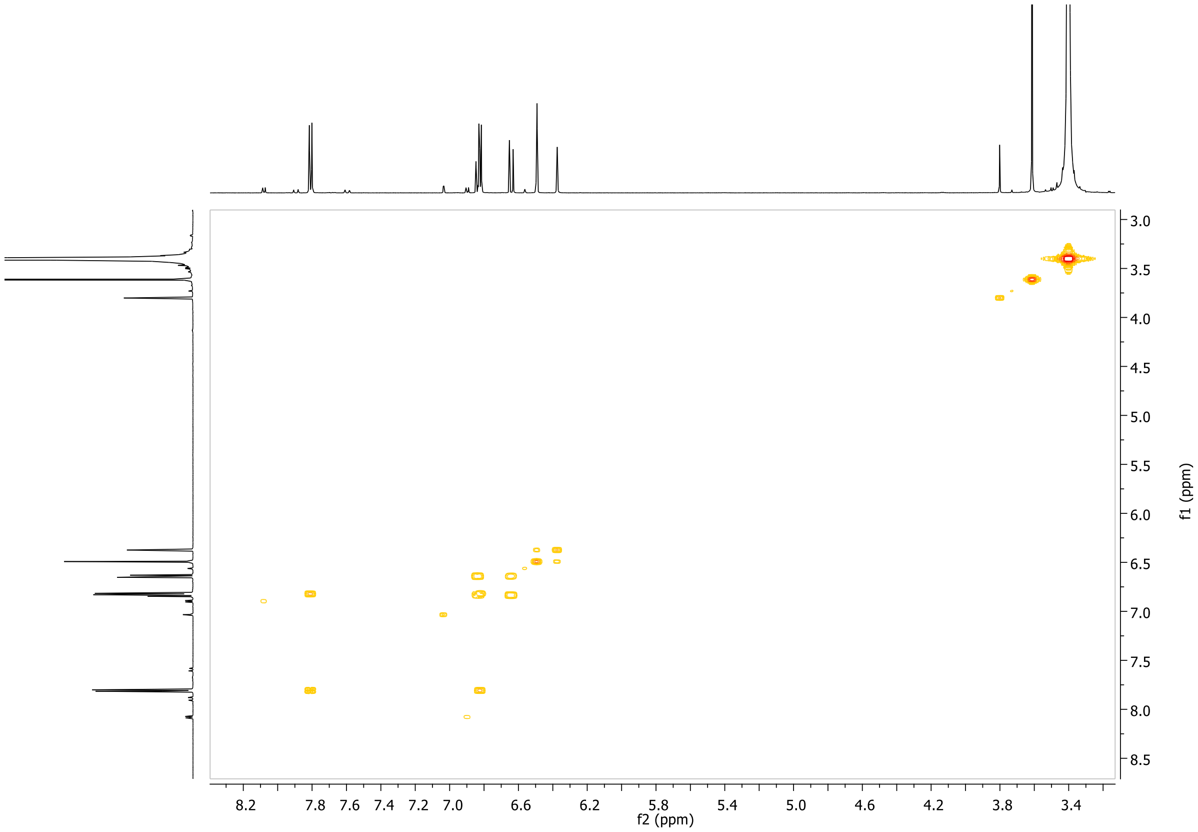

Figure S89. COSY NMR spectrum of *cis*-4′-hydroxy-3,5-dimethoxychalcone (*cis*-**7**)

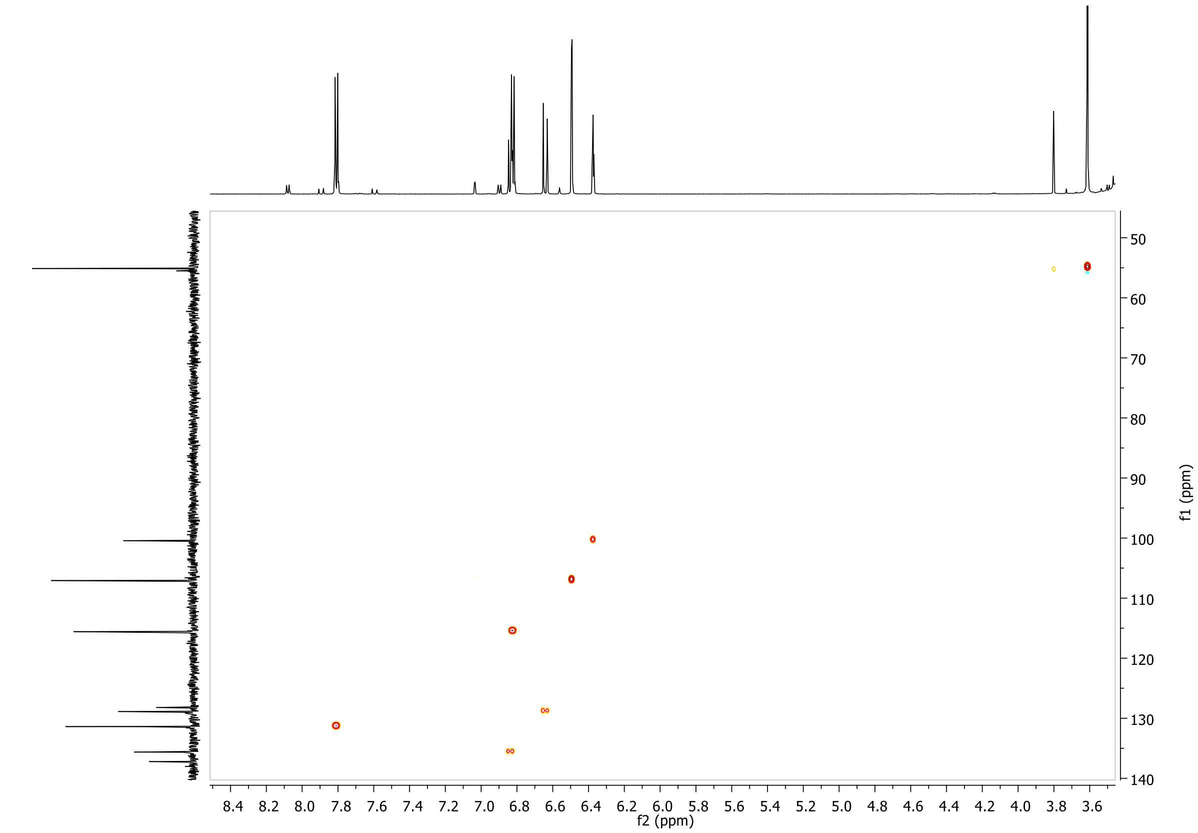

Figure S90. HSQC NMR spectrum of *cis*-4′-hydroxy-3,5-dimethoxychalcone (*cis*-**7**)

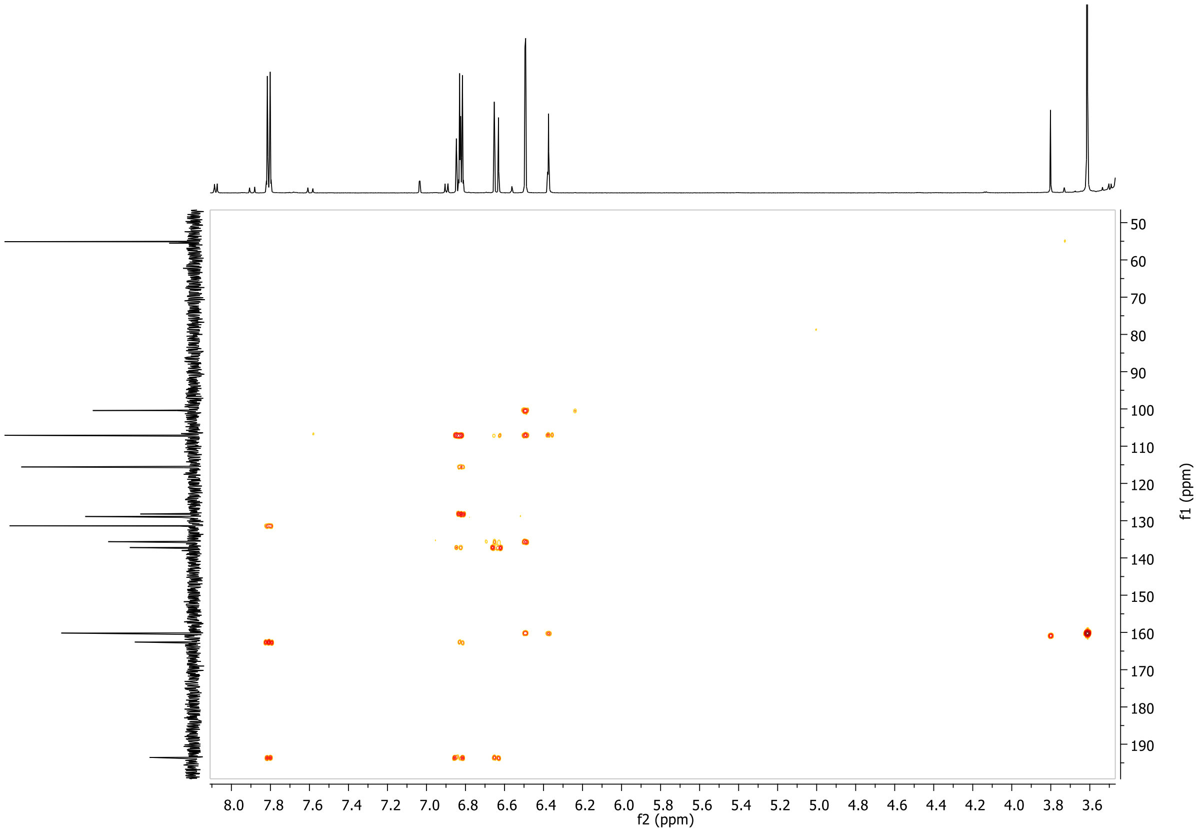

Figure S91. HMBC NMR spectrum of *cis*-4′-hydroxy-3,5-dimethoxychalcone (*cis*-**7**)

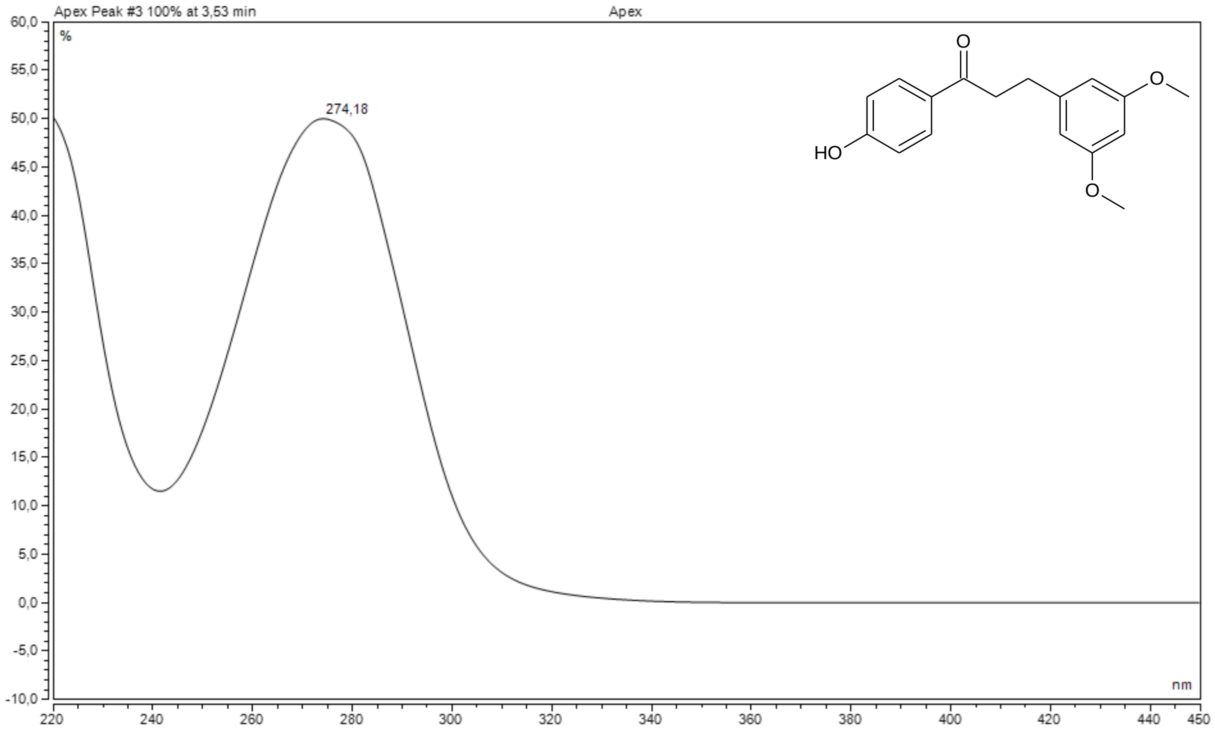

Figure S92. UV spectrum of 4′-hydroxy-3,5-dimethoxydihydrochalcone (**7a**)

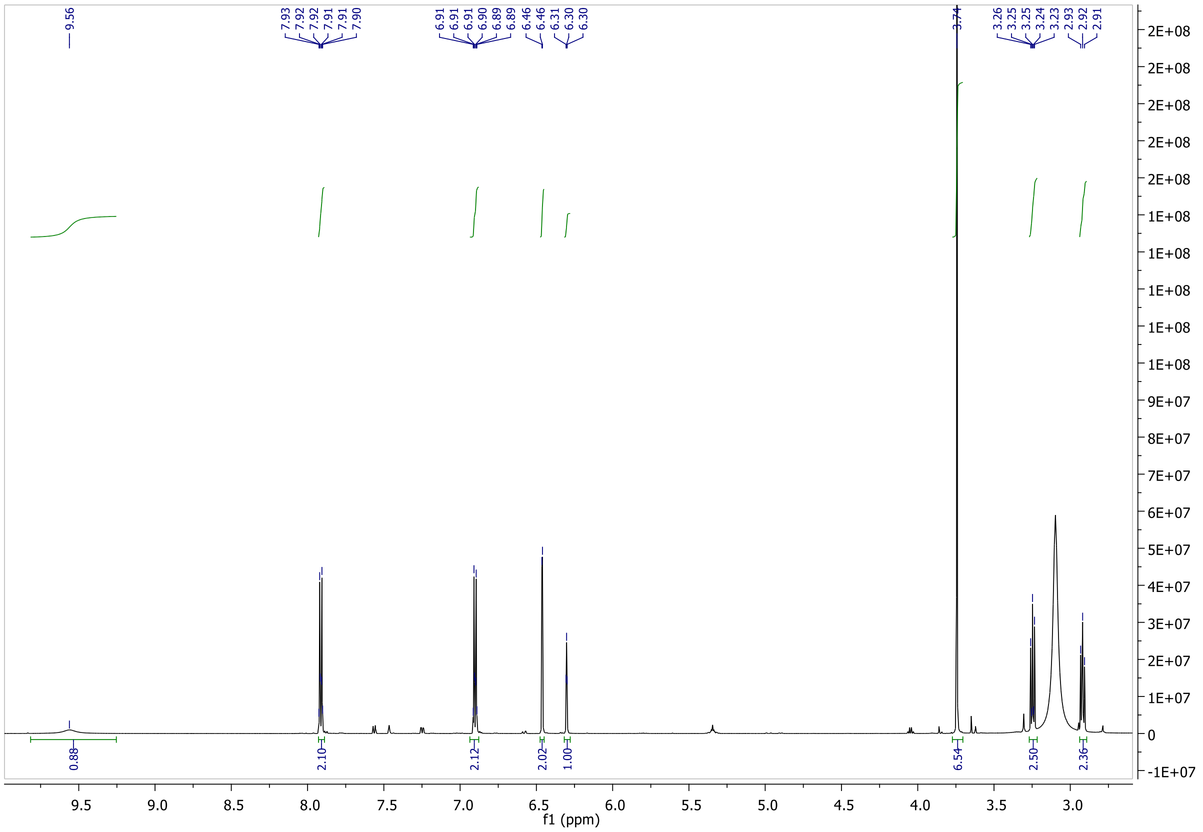

Figure S93. ^{1}H NMR spectrum of 4′-hydroxy-3,5-dimethoxydihydrochalcone (**7a**)

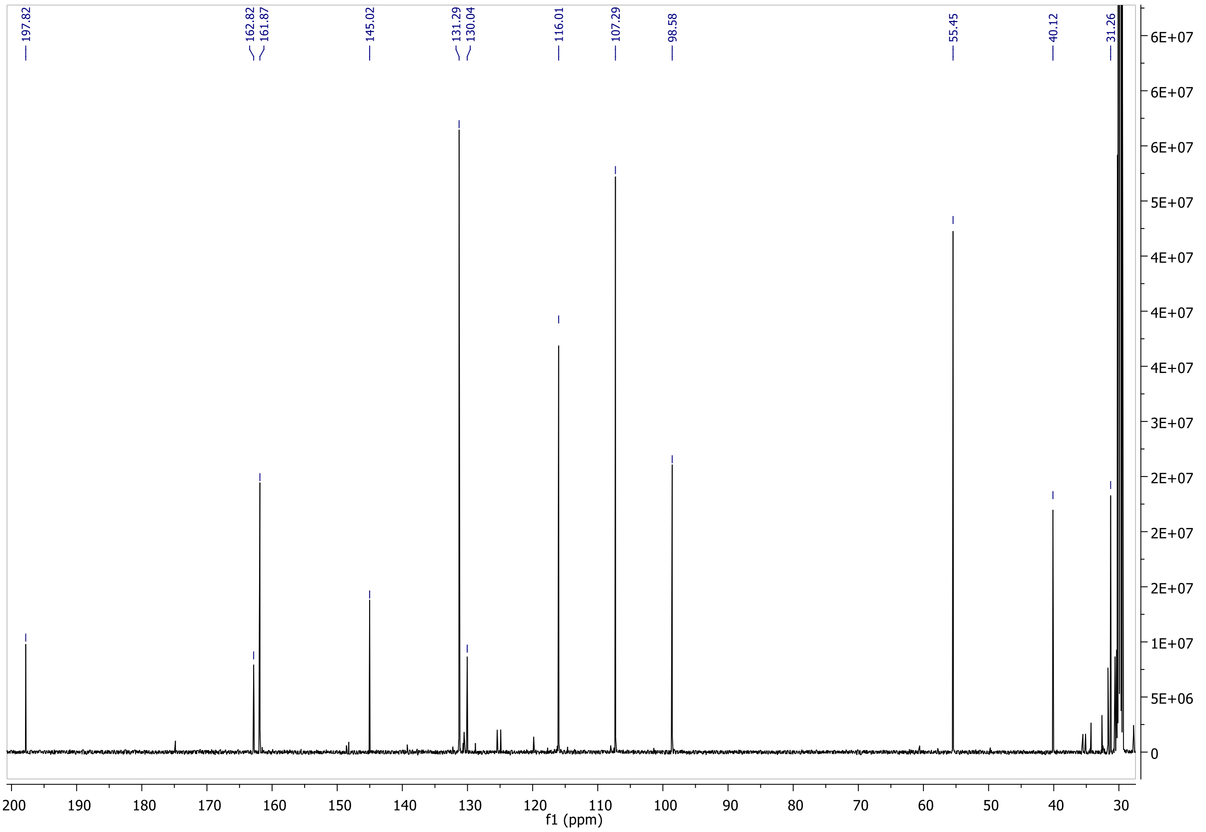

Figure S94. ^{13}C NMR spectrum of 4′-hydroxy-3,5-dimethoxydihydrochalcone (**7a**)

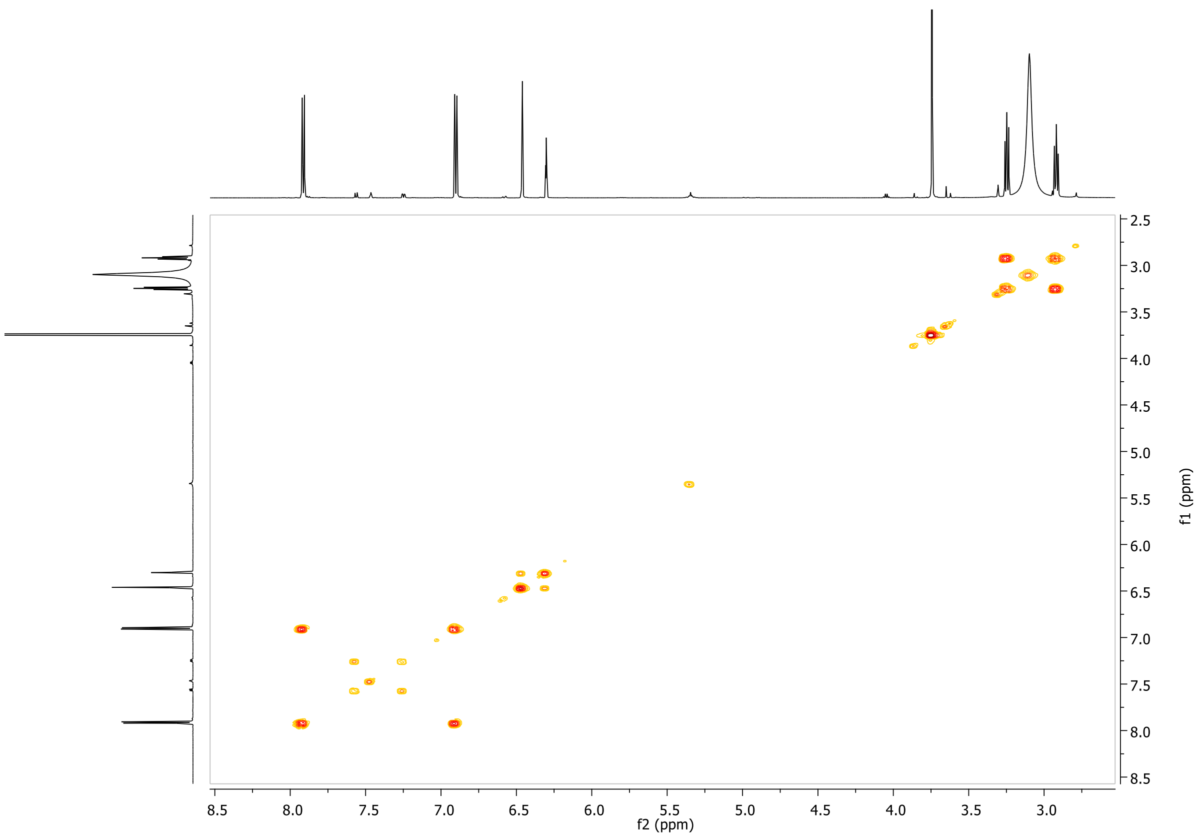

Figure S95. COSY NMR spectrum of 4′-hydroxy-3,5-dimethoxydihydrochalcone (**7a**)

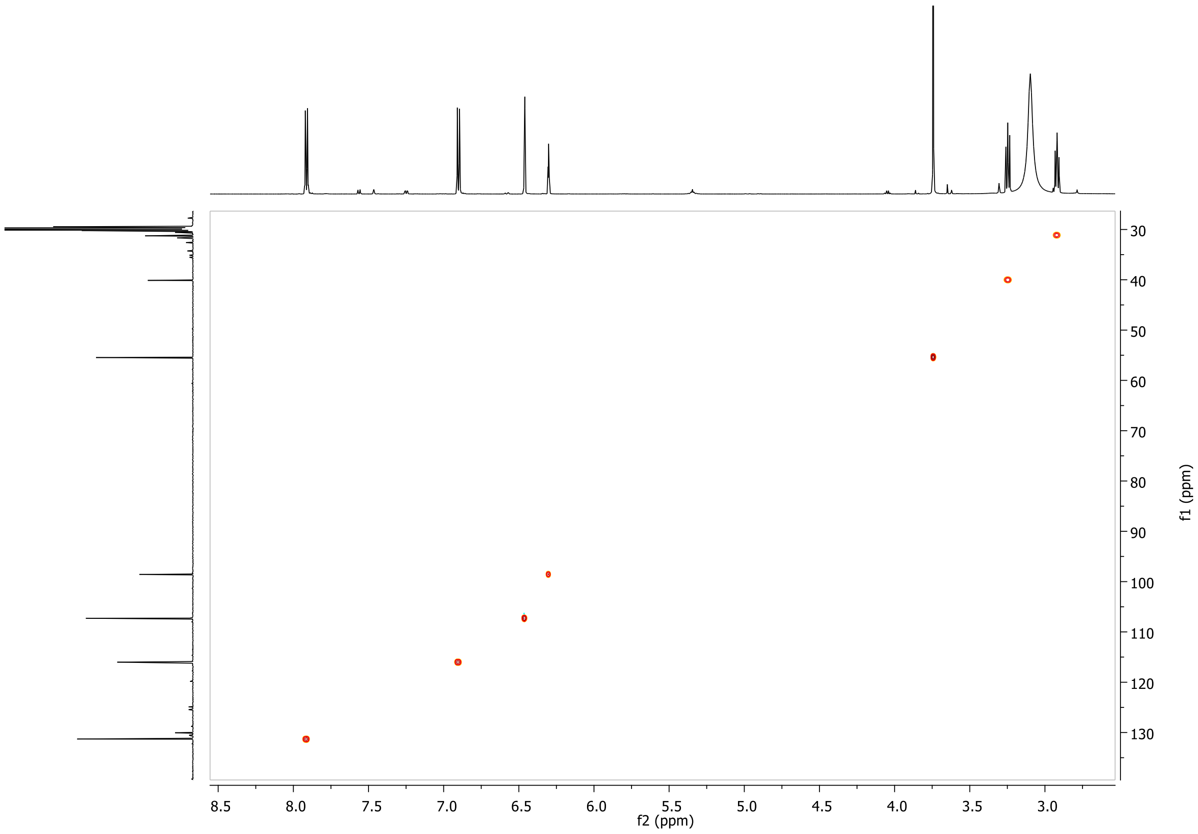

Figure S96. HSQC NMR spectrum of 4′-hydroxy-3,5-dimethoxydihydrochalcone (**7a**)

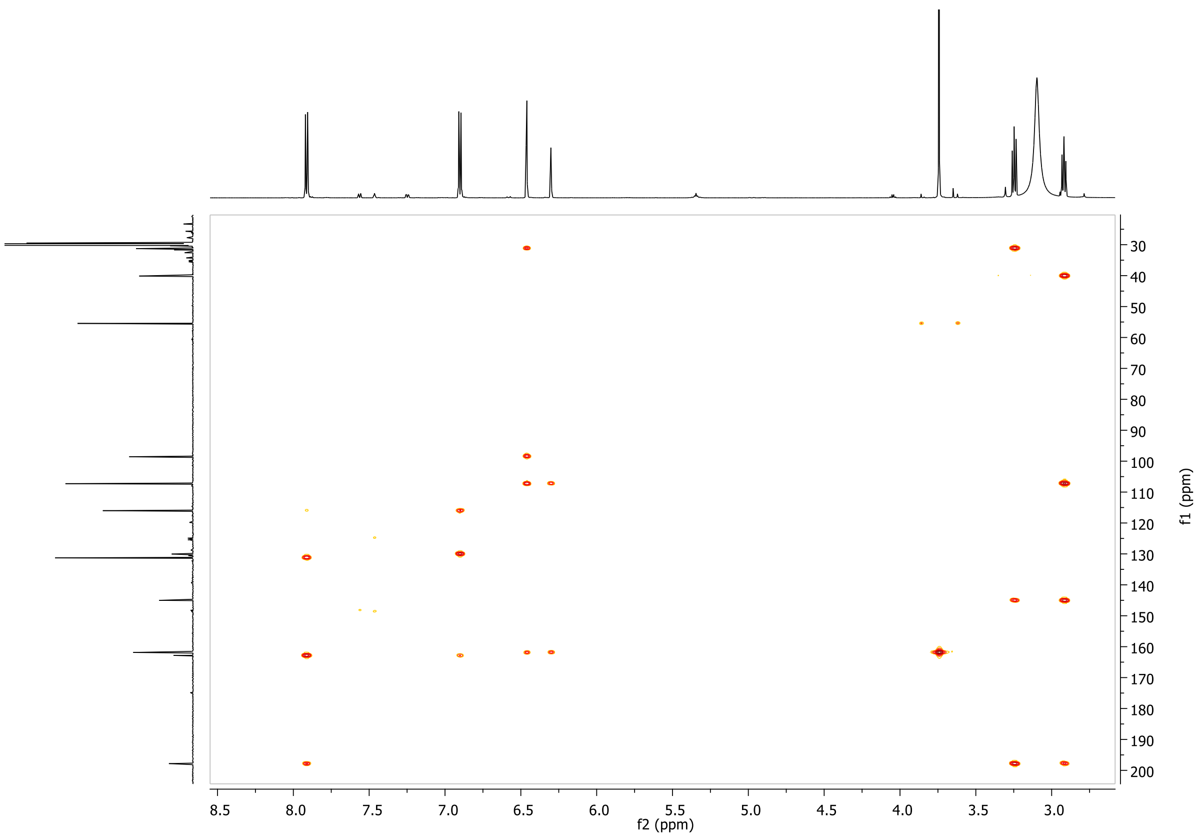

Figure S97. HMBC NMR spectrum of 4′-hydroxy-3,5-dimethoxydihydrochalcone (**7a**)
